# Synthesis and biological evaluation of 1,2-disubsubstituted 4-quinolone analogues of *Pseudonocardia* sp. natural products

**DOI:** 10.3762/bjoc.14.245

**Published:** 2018-10-19

**Authors:** Stephen M Geddis, Teodora Coroama, Suzanne Forrest, James T Hodgkinson, Martin Welch, David R Spring

**Affiliations:** 1 Department of Chemistry, University of Cambridge, Lensfield Road, Cambridge, CB2 1EW, UK; 2 Department of Biochemistry, University of Cambridge, 80 Tennis Road, Cambridge, CB2 1GA, UK; 3 Leicester Institute of Structural and Chemical Biology, and Department of Chemistry, University of Leicester, George Porter Building, University Road, Leicester, LE1 7RH, UK

**Keywords:** antibiotics, cross-coupling, heterocycles, quorum-sensing, structure–activity relationships

## Abstract

A series of analogues of *Pseudonocardia* sp. natural products were synthesized, which have been reported to possess potent antibacterial activity against *Helicobacter pylori* and induce growth defects in *Escherichia coli* and *Staphylococcus aureus*. Taking inspiration from a methodology used in our total synthesis of natural products, we applied this methodology to access analogues possessing bulky N-substituents, traditionally considered to be challenging scaffolds. Screening of the library provided valuable insights into the structure–activity relationship of the bacterial growth defects, and suggested that selectivity between bacterial species should be attainable. Furthermore, a structurally related series of analogues was observed to inhibit production of the virulence factor pyocyanin in the human pathogen *Pseudomonas aeruginosa*, which may be a result of their similarity to the *Pseudomonas* quinolone signal (PQS) quorum sensing autoinducer. This provided new insights regarding the effect of N-substitution in PQS analogues, which has been hitherto underexplored.

## Introduction

The quinolone core has long been implemented in structures possessing formidable activity in a broad range of fields, including antibiotics, bacterial signalling and iron metabolism [1]. Structural optimisation of quinolones possessing intriguing properties can lead to the discovery of important drug classes, as demonstrated by the fluoroquinolone antibiotics, which were inspired by the observation of an antibacterial quinolone side-product generated during the synthesis of the antimalarial chloroquine [2].

**Figure 1:**
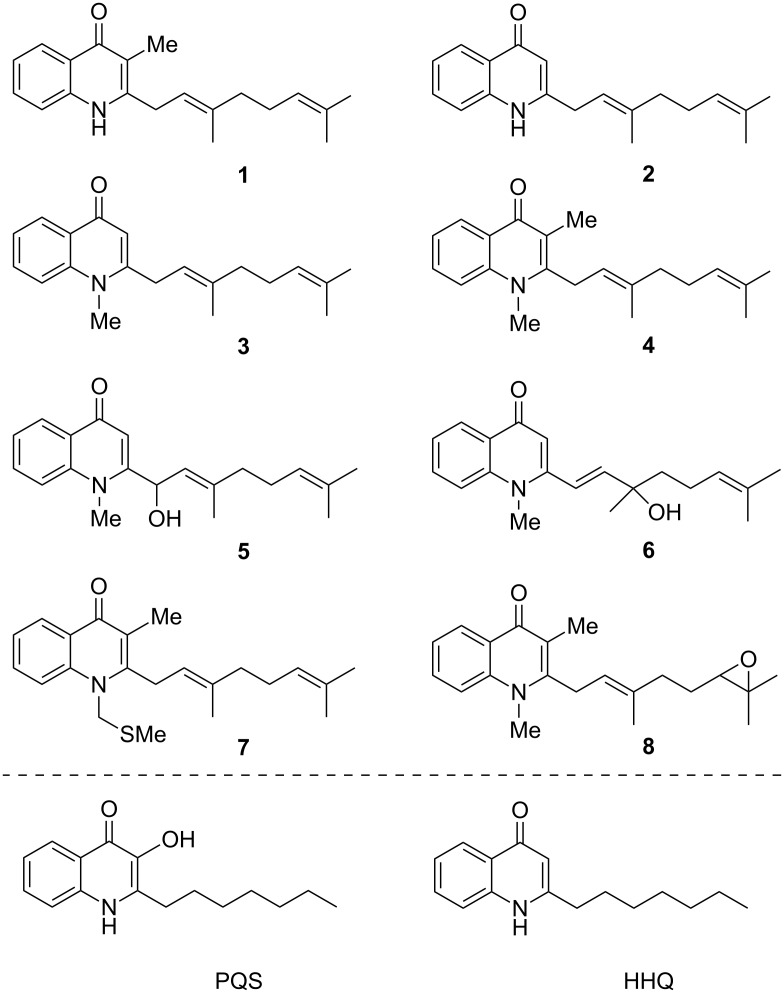
A family of quinolone natural products **1**–**8**, which were first isolated from *Pseudonocardia* sp. CL38489 by Dekker et al. [3], shown alongside the structure of the *Pseudomonas* quinolone signal (PQS), a key autoinducer used in quorum sensing signalling by the human pathogen *Pseudomonas aeruginosa*, and 2-heptyl-4(1*H*)-quinolone (HHQ), the biosynthetic precursor of PQS.

Given this high potential for the discovery of useful chemical entities, we have recently been engaged in research regarding a family of quinolone natural products which are produced by the actinomycete *Pseudonocardia* sp. CL38489, and were first isolated by Dekker et al. (**1**–**8**, Figure 1) [3]. The authors noted the potent antibacterial activity of these compounds against *Helicobacter pylori*, which is responsible for the generation of numerous digestive disorders [4]. Furthermore, with the presence of a lipophilic chain in the 2-position, there is a structural resemblance to the *Pseudomonas* quinolone signal (PQS), and its biosynthetic precursor 2-heptyl-4(1*H*)-quinolone (HHQ), which are vital to the cooperative behaviour of the human pathogen *Pseudomonas aeruginosa* via quorum sensing (QS). This is a means by which bacteria alter their phenotype in response to changes in population density, regulating virulence and biofilm formation when most impactful to the host organism [5]. This process is mediated by signalling molecules such as PQS, and natural product structures **1**–**8** analogous to PQS may provide interspecies QS-modulator chemical probes. It has been proposed that such a strategy may perturb bacterial virulence and pathogenicity associated with QS, thus conferring a therapeutic benefit, without applying a selection pressure for resistance [6]. Whilst recent experiments suggest that resistance may still emerge, it has been proposed that this development should be limited under certain conditions [7].

We wished to investigate the potential of **1**–**8** to modulate QS in *P. aeruginosa*, however, the compounds are available in only trace amounts from natural sources [3], and so we embarked on the total synthesis of the compounds. We first developed a strategy which constructed natural products **1**–**4** by uniting the quinolone cores with the side chain by means of an sp^2^–sp^3^ Suzuki–Miyaura coupling reaction [8]. Whilst these compounds unfortunately provided no modulation of PQS quorum screening (as determined using a heterologous *Escherichia coli* reporter system [9]), an intriguing effect upon the growth of *E. coli* and *Staphylococcus aureus* was noted, which showed an extended lag phase in response to the compounds (except **4**, which was inactive towards *E. coli*. It should be noted that in this previous publication, the graphical data for compounds **3** and **4** was erroneously switched). It is tentatively proposed that this is as a result of disruption of electron transport, as the compounds bear resemblance to the menaquinones which are used by bacteria for this purpose [10]. Following on from this, we recently reported a divergent strategy which granted access to remainder of the natural products **5**–**8**, alongside offering more efficient synthesis of **1** and **4** [11]. Allylic alcohols **5** and **6** were accessed from a mutual precursor (constructed using methodology adapted from that reported by Bernini et al. [12]) using an acid-catalysed transposition, whilst **4**, **7** and **8** were derivatised from **1**.

**Scheme 1:**
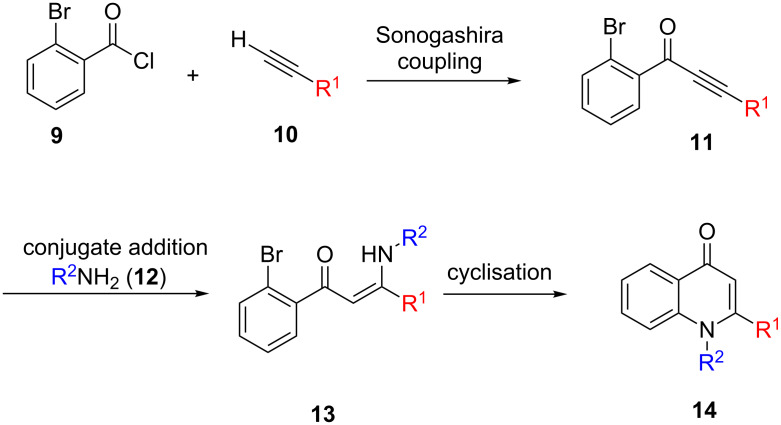
Proposed use of the chemistry developed towards the total synthesis of **5** and **6** for generation of natural product analogues. Modular coupling of alkynes **10** and amines **12** with commercially available acid chloride **9** was proposed to give 1,2-disubstituted quinolones **14**.

In this current work, we turn to the further elucidation of the biological activity of this class of compounds. In order to gain additional insight into the associated structure–activity relationships (SAR), it was desired to generate analogues of the natural products. The chemistry developed towards the allylic alcohols **5** and **6**, outlined in Scheme 1, seemed ideal to this end. A range of alkynes **10** could undergo Sonogashira coupling with the commercially available acid chloride **9**. The resultant ynones **11** could then undergo conjugate addition with primary amines **12**, which following metal-catalysed cyclisation would give 1,2-disubstituted quinolones **14**.

Upon the successful synthesis of analogues of the form **14**, biological evaluation of these and the natural products **1**, **4** and **5**–**7** (synthesised during our previous study [11]) would then be possible. In particular, it was desired to further probe the intriguing growth defects which had been observed for natural products **1**–**4** in *E. coli* and *S. aureus*. Furthermore, exploration of any effect on QS of the analogues **14** would be valuable, as to our knowledge studies on the SAR of PQS analogues have not yet thoroughly assessed substitution at the 1-position of the quinolone system [13]. This is perhaps as a result of direct alkylation at this position being very challenging, with low yields and poor O- vs N-selectivity being encountered, particularly with a sterically demanding substituent present in the 2-position [14–15].

As a measure of modulation of QS in *P. aeruginosa*, it was desired to measure the amount of pyocyanin produced by bacterial cultures after treatment with the compounds. This virulence factor is known to be under the regulation of PQS signalling system, and is capable of disrupting many important biochemical processes [16]. This leads to numerous deleterious effects on human cells, including inhibited respiration and ciliary action [17]. These effects allow pyocyanin to play a critical role in infection; indeed, mutant *P. aeruginosa* strains which are unable to produce pyocyanin have been shown to be unsuccessful in infecting the lungs of mice [18]. Being able to prevent the production of pyocyanin could therefore be of great therapeutic benefit.

## Results and Discussion

**Scheme 2:**
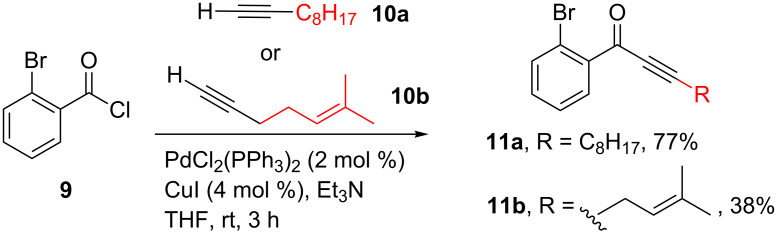
Sonagashira coupling of alkynes **10a** and **10b** with commercially available acid chloride **9** to give ynones **11a** and **11b**.

In the implementation of the strategy outlined in Scheme 1, alkynes **10a** and **10b** were first subjected to Sonogashira coupling with commercially available acid chloride **9** according to the previously reported conditions (Scheme 2) [12]. These alkynes were chosen so as to allow access to valuable SAR data regarding the side chain of the natural products **1**–**8**. Commercially available alkyne **10a** would ultimately lead to a simple saturated side chain of the same length as that observed naturally, whilst **10b** (itself synthesised according to a literature procedure [19]) would provide analogues possessing a truncated prenyl-type substituent. In the event, ynone **11a** was obtained with good yield, however, a poorer yield resulted for **11b**, which was attributed to difficulties in obtaining its precursor **10b** with high purity which stemmed from its volatility.

**Scheme 3:**
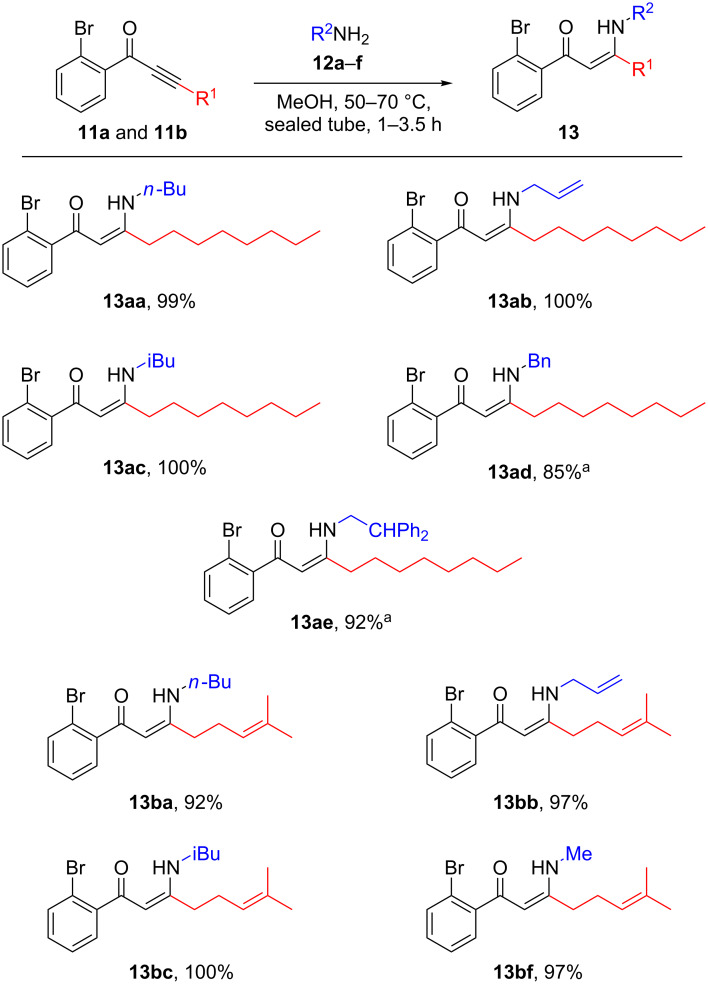
Conjugate addition of primary amines **12a**–**f** with ynones **11a** and **11b**. ^a^Following concentration in vacuo, further purification using silica gel flash chromatography was required.

These ynones were then subjected to a conjugate addition with an assortment of primary amines **12a**–**f** (Scheme 3). The reactions proceeded with excellent yield in all cases, with aliphatic and aromatic moieties well tolerated. Given the high volatility of most of the amine starting materials, the products **13** were in general analytically pure following concentration in vacuo of the reaction mixture. However, use of higher boiling-point amines necessitated purification by flash column chromatography, which may account for the slightly lower yields in these cases (**13ad** and **13ae**).

With the compounds **13** now in hand, their cyclisation to the desired analogues **14** was explored. However, whilst the conditions which had proved successful in the total synthesis of natural products **5** and **6** proved satisfactory in most cases, some optimisation was required for substrates possessing unsaturated functionality attached to the amine (Table 1). When the palladium-catalysed conditions were employed [20], a complex mixture resulted, from which no product could be obtained (Table 1, entry 1). Meanwhile, use of base-induced S_N_Ar-type conditions allowed a small amount of product to be isolated (Table 1, entry 2) [21], but copper-catalysed conditions offered a higher yield (Table 1, entry 3) [12]. This behaviour stands in contrast to that noted for substrates bearing an alkyl substituent in our previous study, for which these copper-catalysed conditions resulted in dimerization [11].

**Table 1:** Optimisation of conditions for the cyclisation of **13ad** to natural product analogue **14ad**.

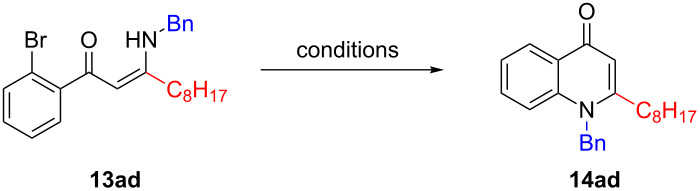		

entry	conditions	result

1	Cs_2_CO_3_, Pd_2_dba_3_, P(2-furyl)_3_, toluene, 100 °C, 24 h [20]	complex mixture^a^
2	KO*t*-Bu, dioxane, 90 °C, 24 h [21]	13%^b^
3	CuI, DMEDA, NaO*t*-Bu, DMSO, 80 °C, 2 h [12]	22%^b^

^a^As determined by LCMS and ^1^H NMR analysis of the crude reaction product. ^b^Isolated yield.

**Scheme 4:**
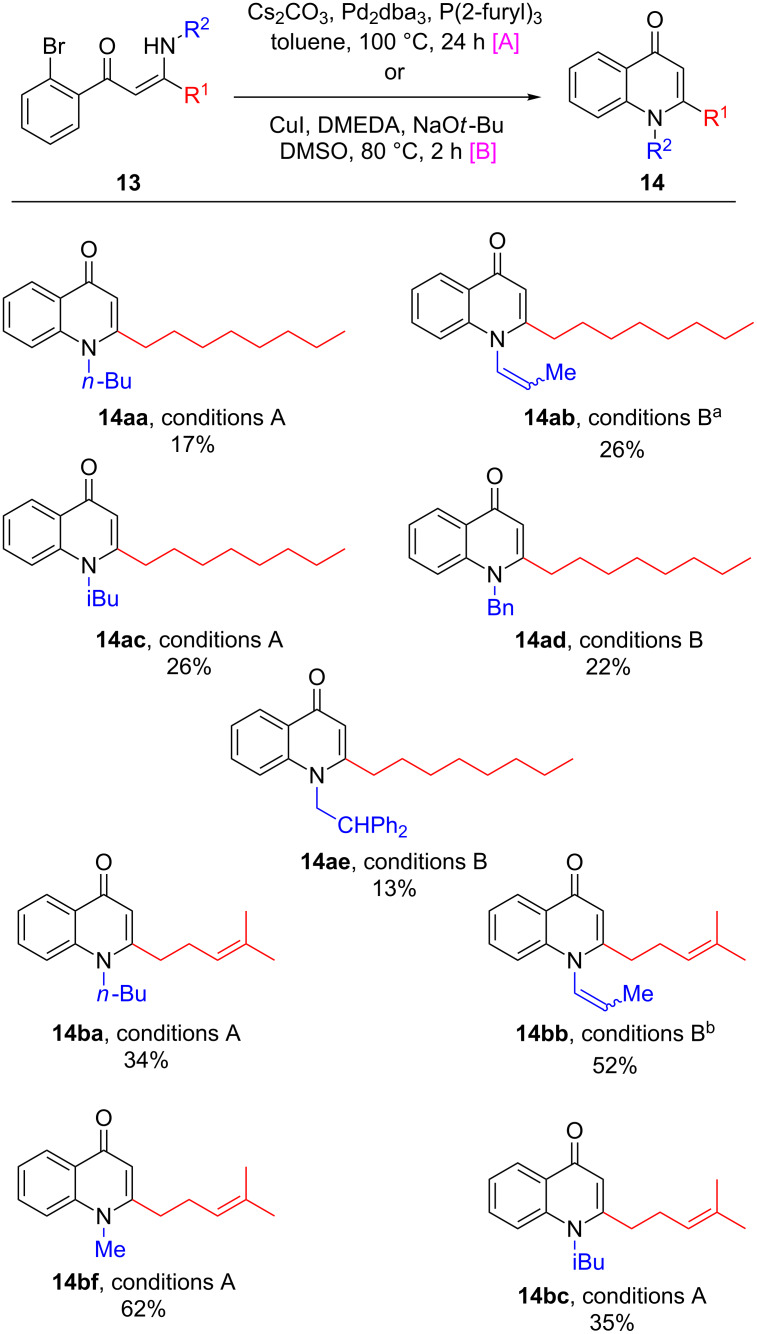
Cyclisation of precursors **13** to natural product analogues **14** using palladium- or copper-catalysed conditions. Yields quoted are isolated. ^a^**13ab** used as starting material, *E*/*Z* ratio = 29:71 based on ^1^H NMR data. ^b^**13bb** used as starting material, *E*/*Z* ratio = 15:85 based on ^1^H NMR data.

Following the discovery of this substrate-dependent dichotomy with respect to optimal reaction conditions, the entire library of compounds was successfully cyclised. Whilst the yields ranged from low to moderate (Scheme 4), sufficient quantities were obtained to facilitate biological screening. It appeared that bulkier N-substituents (e.g., **14ae**) resulted in lower yields than less bulky derivatives (e.g., **14bf**), underlining the importance of steric factors during cyclisation. Interestingly, the allyl-substituted substrates **13ab** and **13bb** underwent an isomerisation under the reaction conditions, with the double bond moving into conjugation with the amine to give inseparable mixtures of enamine-type products **14ab** and **14bb**. Given the likely hydrolytic instability of synthetic precursors possessing an enamine moiety, these compounds would likely be highly challenging to synthesise by other means. However, it is proposed that the involvement of the nitrogen lone-pair in the aromaticity of the quinolone system attenuates the susceptibility of **14ab** and **14bb** towards hydrolysis.

**Scheme 5:**
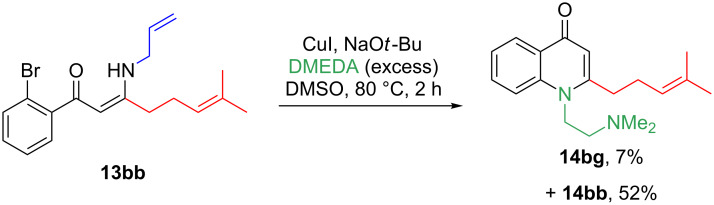
Use of an excess of DMEDA in the Cu-catalysed cyclisation of **13bb** resulted in the generation of **14bg** as a side-product, alongside **14bb**.

Intriguingly, the employment of an excess of DMEDA in the Cu-catalysed cyclisation of **13bb** generated **14bg** as a side-product, which represents another interesting analogue for biological study (Scheme 5). This may putatively result from the displacement of the allylamine in **13bb** by the DMEDA ligand, followed by heterocyclisation with concomitant N*–>*N’ methyl transfer.

**Figure 2:**
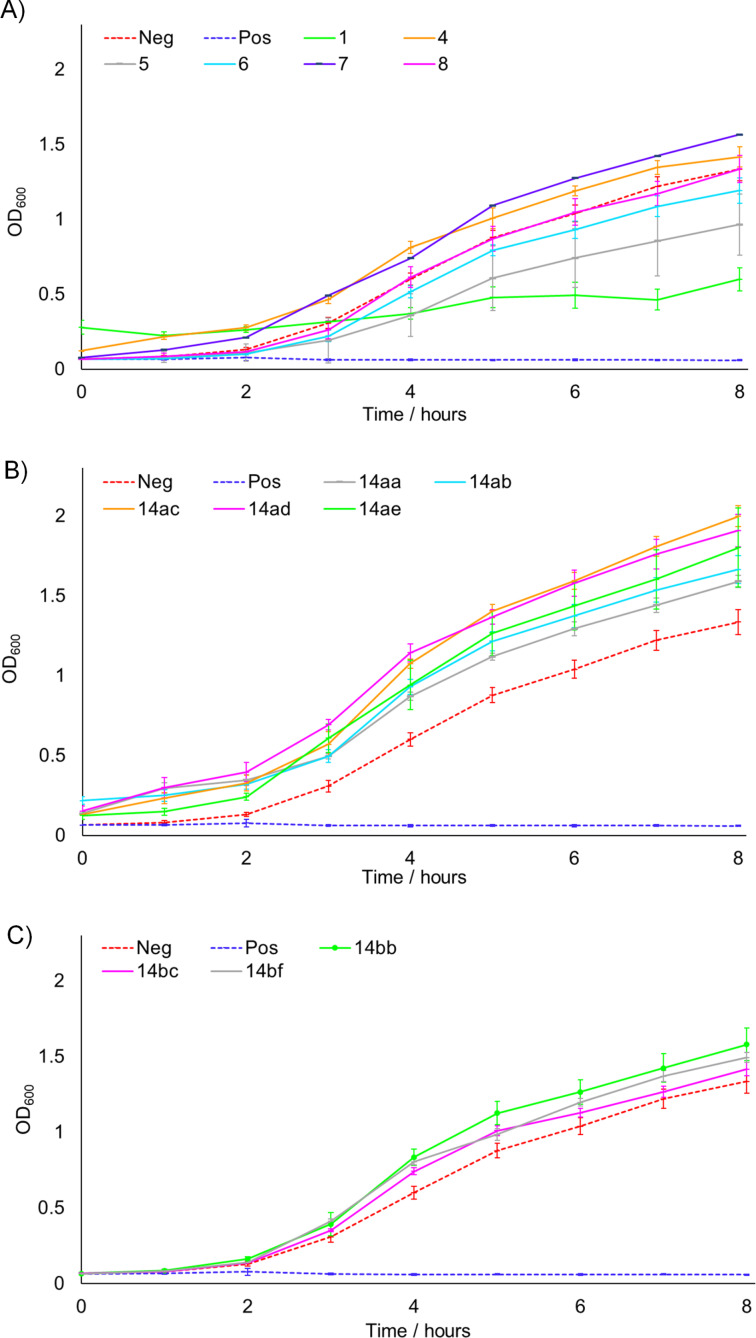
Growth of *E. coli* ESS with time in the presence of 200 μM of each compound. A) Natural product series. B) Saturated analogue series. C) Truncated analogue series. Neg = negative control (DMSO blank), Pos = positive control (gentamicin). Experiments were carried out in triplicate, with the exception of **7**, for which there was insufficient material for repeats. Error bars refer to standard deviation.

With the library of natural products and analogues in hand, our attention turned to their biological activity. It was desired to further explore the growth defects which had been previously noted for natural products **1**–**4** against *E. coli* and *S. aureus*, and so these species were grown in the presence of the compounds. The results for *E. coli* ESS are shown in Figure 2, split into the natural product series, the series of analogues with a saturated side chain, and the truncated series of analogues. As can be seen in Figure 2A, natural product **1** resulted in slowed bacterial growth whilst **4** elicited no such effect, consistent with our previous observations (although the later recovery in population in the presence of **4** was less pronounced in the present case) [8]. Meanwhile, **5** appeared to show a moderate growth-slowing effect, which when compared to the stronger effect previously observed for **3**, demonstrates that oxidation of the geranyl side chain is deleterious to the biological effect under investigation. The regioisomeric **6** showed a very small effect, further showing the lack of tolerance of the effect towards side-chain oxidation. Finally, neither replacement of the N-Me of **4** with the methylthiomethylene substituent of **7**, nor epoxidation of **4**’s side chain to give **8**, offered any improvement in the biological activity. The result for **8** is particularly intriguing, as this natural product was noted to have the strongest effect upon the growth of *H. pylori* in the study by Dekker et al., which may imply that these compounds are acting through different mechanisms upon each bacterial species. Next, considering Figure 2B and C, we see that none of the analogues were capable of affecting the growth of *E. coli*, further demonstrating the importance of the geranyl side for biological activity against this species. This observation is particularly striking for analogue **14bf**, which possesses an identical quinolone core structure to natural product **3**, which was previously shown to be active.

**Figure 3:**
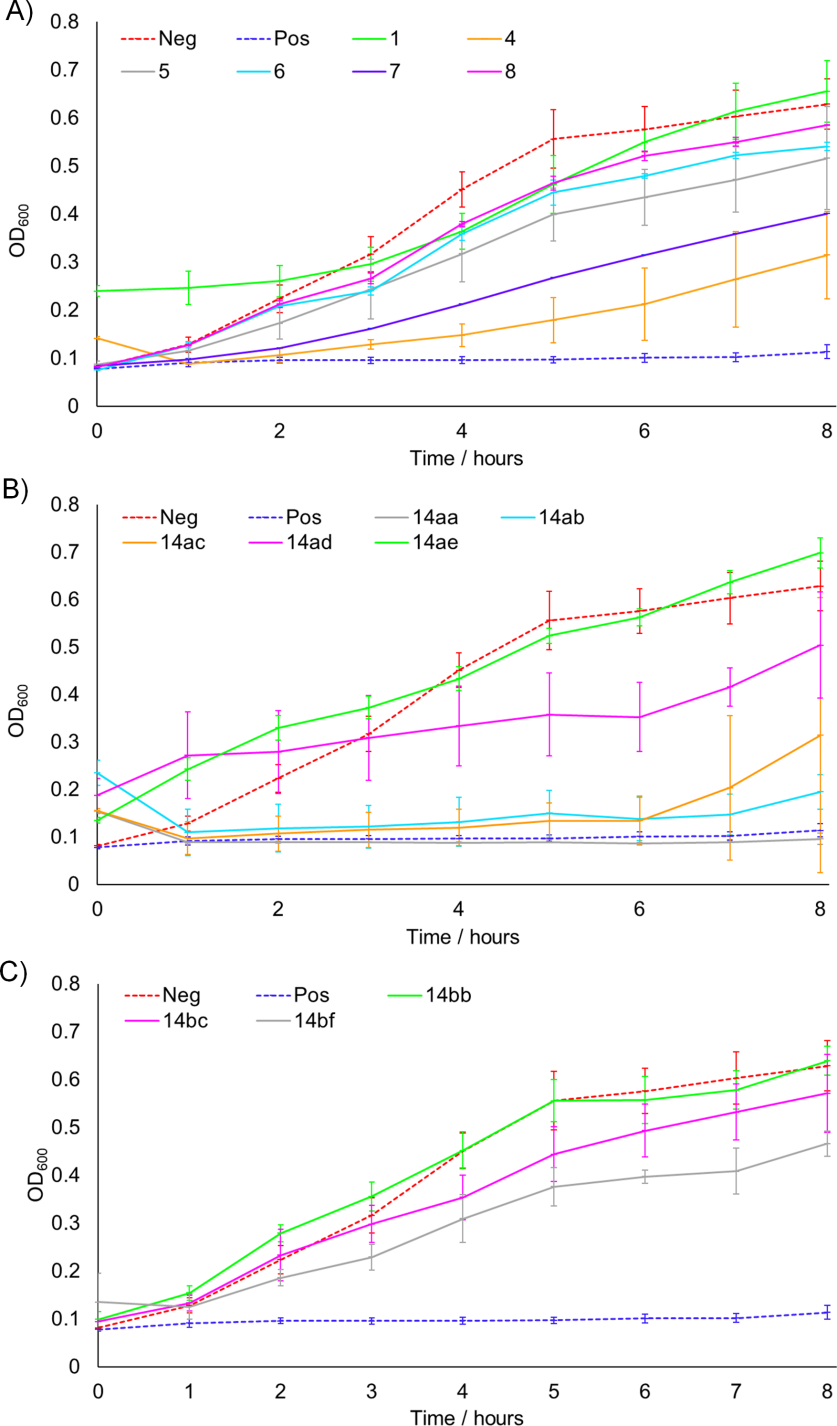
Growth of *S. aureus* 25923 with time in the presence of 200 μM of each compound. A) Natural product series. B) Saturated analogue series. C) Truncated analogue series. Neg = negative control (DMSO blank), Pos = positive control (gentamicin). Experiments were carried out in triplicate, with the exception of **7**, for which there was insufficient material for repeats. Error bars refer to standard deviation.

Meanwhile, Figure 3 shows the data for *S. aureus* 25923, which is split into the same three series as before. Considering the natural products (Figure 3A), we see that both **1** and **4** resulted in a slowing of growth consistent with that reported previously, although the effect for **1** was less pronounced in the present case, operating for only the first three hours [8]. Whilst most of the other natural products appeared to show only slight effects, moderate activity was observed for **7**, which stands in an interesting contrast to the inactivity of this compound against *E. coli*. This implies that the structural requirements for optimal activity differ between the species, a conclusion which is further bolstered by the results for the saturated series of analogues shown in Figure 3B. Whilst these compounds were completely inactive against *E. coli*, in this case a strong effect was observed, which for **14aa**, **14ab** and **14ac** was comparable to the positive gentamicin control over the timescale of concern. These results show the high efficacy of the saturated side chain against *S. aureus*, however, considering the data for **14ad** and **14ae**, we can observe that adding bulky aromatic moieties to the N-substituent results in reduced activity, with smaller alkyl groups in this position instead being optimal. Finally, the truncated series of analogues appeared to show only small effects upon the growth of *S. aureus* (Figure 3C).

**Figure 4:**
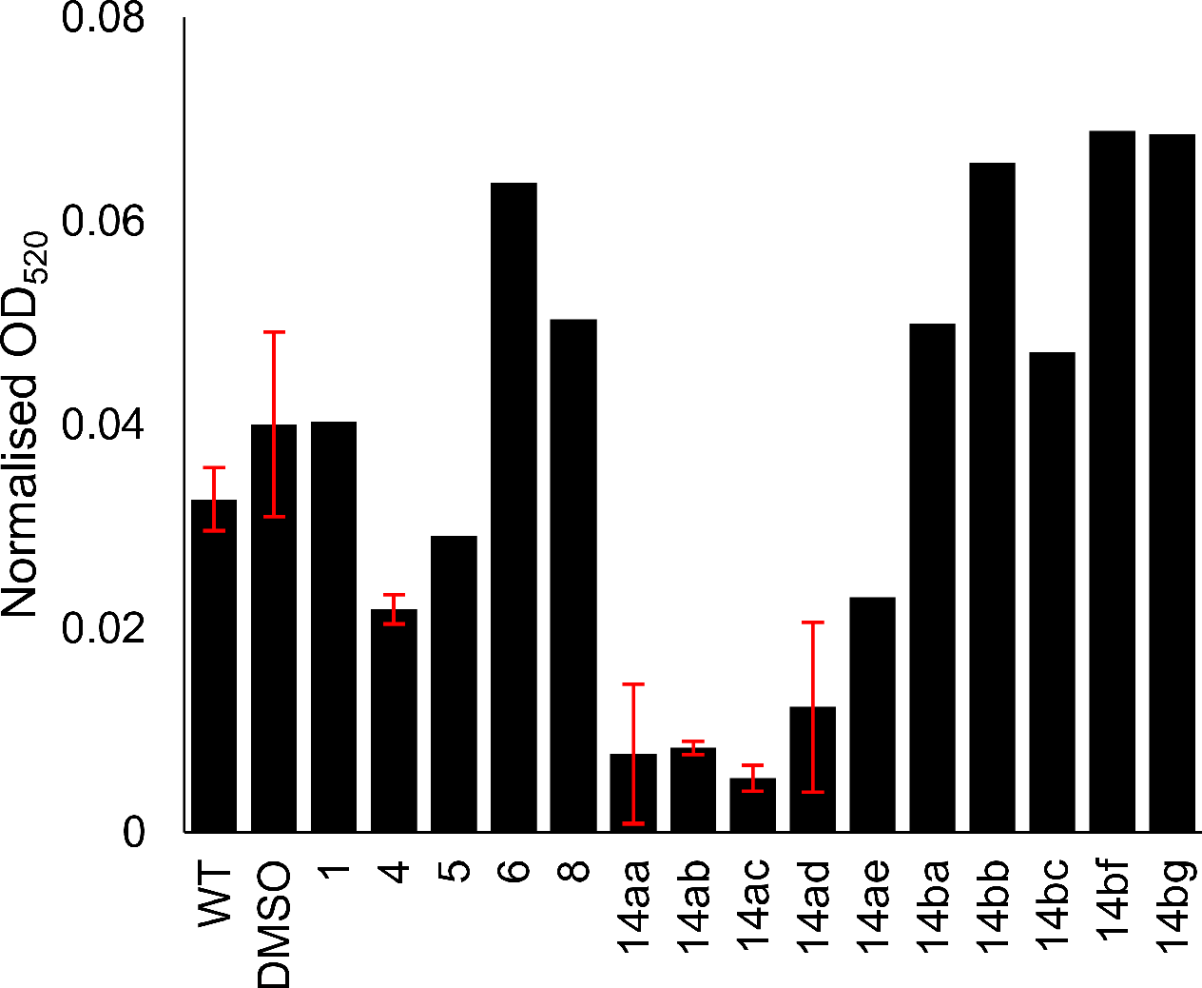
OD_520_ (absorption corresponding to pyocyanin) normalised by the culture population (measured by OD_600_) for cultures of *P. aeruginosa* PA01 grown in the presence of concentrations of 200 μM of natural products and analogues after 8 hours. WT = wild type, no treatment added. DMSO = treated with DMSO blank. The experiment for **7** was not performed due to lack of material. The most promising results were replicated once to ensure validity, as shown by error bars.

Attention then turned to the ability of the compounds to modulate *P. aeruginosa* PA01 QS, as measured by the production of the virulence factor pyocyanin. Bacterial cultures were grown for eight hours in the presence of each compound, followed by extraction of the pyocyanin under acidic conditions [22]. This was then quantified by measurement of the OD_520_, which corresponds to absorption by the toxin. The results are shown in Figure 4, normalised by the bacterial density as measured by OD_600_ (no significant effect on the overall growth of the bacteria was observed for any of the compounds, see Supporting Information File 1 for details). The most promising results were replicated to ensure validity (due to the large amount of chemicals required for the assay, it was not practical to perform this for every compound). Of the natural products, only **4** seemed to show attenuation of pyocyanin production relative to the negative control. Meanwhile, whilst the truncated series of analogues appeared to lack activity, a marked reduction in pyocyanin production was noted for the compounds possessing a linear octanyl side chain. We speculate that this is due to the similarity to the heptanyl chain present in the native PQS ligand. This observation is highly intriguing, as HHQ (Figure 1), which differs from these analogues only by the lack of an N-substituent and a slightly shortened side chain, is known to activate *P. aeruginosa* QS (although it is incapable of inducing full pyocyanin expression) [23]. It would therefore appear that this inhibitory activity is likely due to the N-substitution, an avenue which has been to our knowledge underexplored in the SAR analysis of PQS and HHQ. In particular, analogues possessing smaller N-substituents (**14aa**, **14ab** and **14ac**) appeared to elicit a stronger effect than those possessing larger aromatic moieties (**14ad** and **14ae**).

## Conclusion

We have reported the synthesis of structural analogues of a family of 4-quinolone *Pseudonocardia* sp. natural products, which encompassed variation of both the side chain and N-substituent. This represented an extension of the chemistry which we employed towards the natural products, utilising sequential Sonogashira coupling, high-yielding conjugate addition, and metal-catalysed cyclisation. A dichotomy in the optimal conditions for the final step was discovered, depending on the nature of the N-substituent. These analogues were then tested, together with a number of previously synthesised natural products, for their ability to bring about an intriguing “growth-slowing” effect towards certain species of bacteria. Whilst the presence of a geranyl side chain was shown to be vital for strong activity in *E. coli*, analogues possessing a saturated side chain exhibited marked inhibition of *S. aureus* growth. This intriguing result implies that slightly different mechanisms may be at work in each case, and suggests that it may be possible to attain selective therapeutic treatment of a specific species. Furthermore, the saturated series of analogues were demonstrated to inhibit the production of pyocyanin by *P. aeruginosa*, a virulence factor known to be under QS regulation, providing valuable new SAR insights regarding N-substitution of PQS and HHQ analogues.

## Supporting Information

File 1 Experimental procedures and analytical data.
