# Supplementary material for: Synthesis and biological evaluation of 1,2-disubsubstituted 4-quinolone analogues of Pseudonocardia sp. natural products
Source: Beilstein J Org Chem. 2018 Oct 19;14:2680–8. doi: 10.3762/bjoc.14.245 (PMC6204808; doi:10.3762/bjoc.14.245)
Supplement: File 1 — Experimental procedures and analytical data. [file Beilstein_J_Org_Chem-14-2680-s001.pdf]

Supporting Information

for

Synthesis and biological evaluation of 1,2-disubsubstituted 4-quinolone analogues of *Pseudonocardia* sp. natural products

Stephen M. Geddis[1], Teodora Coroama[1], Suzanne Forrest[2], James T. Hodgkinson*[3], Martin Welch*[2] and David R. Spring*[1]

Address: [1]Department of Chemistry, University of Cambridge, Lensfield Road, Cambridge, CB2 1EW, UK, [2]Department of Biochemistry, University of Cambridge 80 Tennis Road, Cambridge, CB2 1GA, UK and [3]Leicester Institute of Structural and Chemical Biology, and Department of Chemistry, University of Leicester, George Porter Building, University Road, Leicester, LE1 7RH, UK

Email: James T. Hodgkinson - jthodgkinson@leicester.ac.uk; M. Welch - mw240@cam.ac.uk; David Spring - spring@ch.cam.ac.uk

* Corresponding author

Experimental procedures and analytical data

General experimental

All non-aqueous reactions were performed under argon using glassware that had been oven-dried overnight. Standard practices were employed when handling moisture- and air-sensitive materials.

Room temperature (rt) refers to ambient temperature. All temperatures below 0 °C are that of the external bath. Temperatures of 0 °C were maintained using an ice-water bath. Temperatures of −78 °C were maintained using an acetone-cardice bath. Temperatures of reactions performed in sealed tubes refer to the temperature of the external silicone oil bath.

All reagents and solvents were used as received unless otherwise stated. CH_2Cl_2, EtOAc, MeOH, MeCN and toluene were distilled from CaH_2. Tetrahydrofuran (THF) was dried over Na wire and distilled from a mixture of $LiAlH_4$ and CaH_2 with triphenylmethane as the indicator. Et_2O was distilled from a mixture of $LiAlH_4$ and CaH_2. Petroleum ether was distilled before use, with pet. ether 40–60 referring to the fraction between 40–60 °C, and pet. ether 30–40 referring to the fraction between 30–40 °C. The ratios of all solvent mixtures are expressed as volume concentrations (v/v). *n*-Butyllithium (*n*-BuLi) in hexanes was titrated with *N*-benzylbenzamide by the method of Chong et al. before use [1].

Yields refer to chromatographically and spectroscopically pure compounds unless otherwise stated. Where possible, reactions were monitored by thin layer chromatography (TLC) and/or liquid chromatography–mass spectrometry (LCMS). TLC analysis was performed on commercially prepared glass plates precoated with Merck silica gel F_{254}. Visualisation was by the quenching of ultraviolet (UV) fluorescence (λ_{max} = 254 nm) or by staining with potassium permanganate or vanillin. Retention factors (R_f) are quoted to the nearest 0.01. LCMS analysis was performed on a Waters ACQUITY H-Class UPLC with an ESCi Multi-Mode Ionisation Waters SQ Detector 2 spectrometer using MassLynx 4.1 software; LC system: solvent A: 2 mM NH_4OAc in H_2O/MeCN 95:5; solvent B: MeCN; solvent C: 2% HCO_2H; gradient: A/B/C, 90:5:5–0:95:5 over 1 min at a flow rate of 0.6 $mL\cdot min^{-1}$.

The naming of compounds and numbering of atoms does not follow IUPAC conventions.

Flash column chromatography (FCC) was carried out using either slurry-packed Merck 938 Keiselgel 60 SiO_2 (230–400 mesh) under a positive pressure of dry nitrogen.

Infrared (IR) spectra were recorded neat on a Perkin-Elmer Spectrum One (FTIR) spectrometer with internal referencing. Selected absorption maxima (ν_{max}) are reported in wavenumbers (cm^{-1}) with the following abbreviations: w, weak; m, medium; s, strong.

Melting points (mp) were obtained on a Büchi B-545 melting point apparatus and are uncorrected.

Magnetic resonance spectra were processed using ACD/NMR Processor Academic Edition v. 12.01 or TopSpin v. 3.5 (Bruker). An aryl, quaternary, or two or more possible assignments were given when signals could not be distinguished by any means.

Proton magnetic resonance spectra were recorded using an internal deuterium lock (at 298 K unless stated otherwise) on Bruker DPX (400 MHz; 1H-13C DUL probe), Bruker Avance III HD (400 MHz; Smart probe), Bruker Avance III HD (500 MHz; Smart probe) and Bruker Avance III HD (500 MHz; DCH Cryoprobe) spectrometers. Proton assignments are supported by ^{1}H,^{1}H COSY, ^{1}H,^{13}C HSQC or ^{1}H,^{13}C HMBC spectra, or by analogy. Chemical shifts (δ) are quoted in ppm to the nearest 0.01 ppm and are referenced to the residual non-deuterated solvent peak. Discernible coupling constants for coupled protons are reported as measured values in Hertz, rounded to the nearest 0.1 Hz. Data are reported as: chemical shift, number of nuclei, multiplicity (br, broad; s, singlet; d, doublet; t, triplet; q, quartet; m, multiplet; or a combination thereof), coupling constants and assignment. Diastereotopic protons are assigned as X and X', where X' designates the lower-field proton.

Carbon magnetic resonance spectra were recorded using an internal deuterium lock (at 298 K unless stated otherwise) on Bruker DPX (101 MHz), Bruker Avance III HD (101 MHz) and Bruker Avance III HD (126 MHz) spectrometers with broadband proton decoupling. Carbon spectra assignments are supported by DEPT editing, ^{1}H,^{13}C HSQC or ^{1}H,^{13}C HMBC spectra, or by analogy. Chemical shifts (δ) are quoted in ppm to the nearest 0.1 ppm and are referenced to the deuterated solvent peak. Data are reported as: chemical shift, multiplicity (if not a singlet), coupling constants and assignment.

High resolution mass spectrometry (HRMS) measurements were recorded on a Micromass QTOF mass spectrometer or a Waters LCT Premier Time of Flight mass spectrometer. Mass values are quoted within the error limits of ±5 ppm mass units. ESI refers to the electrospray ionisation technique.

1-(2-Bromophenyl)undec-2-yn-1-one (11a)

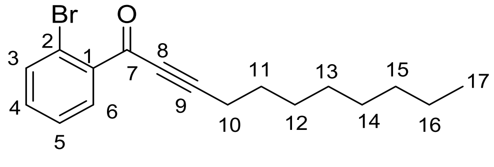

A mixture of 2-bromobenzoyl chloride (0.45 mL, 3.44 mmol), $PdCl_2(PPh_3)_2$ (48 mg, 0.068 mmol) and triethylamine (0.45 mL, 3.23 mmol) in THF (18 mL) was stirred for 15 min. CuI

(21 mg, 0.110 mmol) was added and the mixture stirred 10 more min, then 1-decyne (0.48 mL, 2.66 mmol) was added followed by stirring for 2 h at room temperature. The mixture was then diluted with EtOAc (45 mL), washed with sat. aq. NH_4Cl (40 mL), dried ($MgSO_4$) and concentrated in vacuo. The crude product was purified using silica gel flash chromatography, eluting with 2% ether/pet ether 40–60 to give the product as a dark orange oil (657 mg, 2.04 mmol, 77%).

R*f* (20% Et_2O/petroleum ether 40-60) = 0.67; **^{1}H NMR** (400 MHz, $CDCl_3$): δ = 0.91 (3H, t, *J* = 6.8 Hz, H17), 1.25-1.38 (6H, m, H14, H15, H16), 1.42-1.50 (4H, m, H12, H13)), 1.67 (2H, quin, *J* = 7.2 Hz, H11), 2.48 (2H, t, *J* = 7.2 Hz, H10), 7.37 (1H, td, *J* = 7.5, 2.0 Hz, H4), 7.44 (1H, td, *J* = 7.8, 1.0 Hz, H5), 7.69 (1H, dd, *J* = 7.8, 1.0 Hz, H3), 8.02 (1H, dd, *J* = 7.5, 2.0 Hz, H6); **^{13}C NMR** (100 MHz, $CDCl_3$): δ = 14.1 (C17), 19.3 (C10), 22.7 (C16), 27.6 (C11), 29.0 (C12), 29.0 (C13), 29.1 (C14), 31.7(C15), 80.7 (C8), 98.0 (C9), 121.0 (C1), 127.2 (C5), 132.8 (C4), 133.1 (C6), 134.9 (C3), 137.6 (C2), 177.7 (C7); **υ** (cm^{-1}) (neat) = 2925 (m, C-H), 2854 (m, C-H), 2204 (m, C≡C), 1652 (s, C=O), 1586 (w, C=C Ar), 1564 (w, C=C Ar).

Analytical data consistent with the literature [2].

General Procedure 1

To a solution of ynone (1.0 equiv) in MeOH (0.25 M) was added amine (2 equiv) followed by stirring and heating at 70 °C in a sealed tube. The mixture was then cooled and concentrated in vacuo. The crude product was purified using silica gel flash chromatography.

(*Z*)-3-(Benzylamino)-1-(2-bromophenyl)undec-2-en-1-one (13ad)

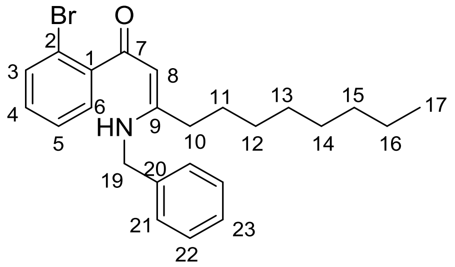

Prepared according to General Procedure 1 for 3 h, using **11a** (0.160 g, 0.50 mmol) and benzyl amine (0.11 mL, 1.01 mmol) as the starting material. The chromatography column was eluted with 20% Et_2O/pet ether 40–60 to give the title compound as a pale yellow viscous oil (181 mg, 0.42 mmol, 85%).

R*f* (20% Et_2O/petroleum ether 40-60) = 0.18; **^{1}H NMR** (400 MHz, $CDCl_3$): δ = 0.90 (3H, t, *J* = 6.8 Hz, H17), 1.23- 1.42 (8H, m, H12-16), 1.55-1.65 (2H, *J* = 7.8 Hz, H11), 2.33 (2H, t, *J* =

7.8 Hz, H10), 4.59 (2H, d, *J* = 6.5 Hz, H19), 5.36 (1H, s, H8), 7.20 (1H, td, *J* = 7.8, 1.7 Hz, H4), 7.30-7.42 (6H, m, H5, H21, H22, H23), 7.46 (1H, dd, *J* = 7.5, 1.7 Hz, H6), 7.59 (1H, dd, *J* = 8.2, 1.0 Hz, H3), 11.57-11.60 (1H, m, N-H); **^{13}C NMR** (100 MHz, $CDCl_3$): δ = 14.1 (C17), 22.6 (C16), 27.9 (C11), 29.1 (C12 or C13 or C14), 29.3 (C12 or C13 or C14), 29.4 (C12 or C13 or C14), 31.8 (C15), 32.2 (C10), 46.8 (C19), 95.5 (C8), 119.5 (C2), 126.9 (C21), 127.1 (C22 or C23), 127.6 (C5) 128.9 (C22 or C23), 129.1 (C6), 129.8 (C4), 133.2 (C3), 137.6 (C20), 143.6(C1), 169.2 (C9), 190.0 (C7); **υ** (cm^{-1}) (neat) = 2923 (m, C-H), 2853 (m, C-H), 1594 (s, C=O), 1567 (s, C=C Ar); **HRMS:** *m*/*z* (ES) calculated for $C_{24}H_{31}BrNO$ $[M+H]^+$: 428.1584, found 428.1585.

(*Z*)-1-(2-Bromophenyl)-3-((2,2-diphenylethyl)amino)undec-2-en-1-one (13ae)

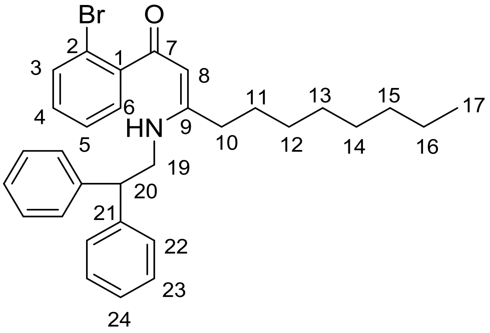

Prepared according to General Procedure 1 for 2 h, using **11a** (0.150 g, 0.47 mmol) and 2,2-diphenylethyl amine (184 mg, 0.94 mmol) as the starting material. The chromatography column was eluted with 15% Et_2O/pet ether 40-60 to give the title compound as white solid (234 mg, 0.43 mmol, 92%).

R_f (20% Et_2O/petroleum ether 40-60) = 0.26; **^{1}H NMR** (400 MHz, $CDCl_3$): δ = 0.88 (3H, t, *J* = 7.0 Hz, H17), 1.22-1.32 (10H, m, H12-H16), 1.44-1.50 (2H, m, H11), 2.09-2.14 (2H, m, H10), 3.95 (2H, dd, *J* = 7.3, 6.1 Hz, H19), 4.26 (1H, t, *J* = 7.6 Hz, H20), 5.16 (1H, s, H8), 7.14 (1H, td, *J* = 7.9, 1.8 Hz, H4), 7.24-7.30 (8H, m, Ar-H), 7.31-7.36 (4H, m, Ar-H), 7.53 (1H, dd, *J* = 7.9, 0.9 Hz, H3), 11.30-11.34 (1H, m, N-H); **^{13}C NMR** (100 MHz, $CDCl_3$): δ = 14.1 (C17), 22.6 (C16), 27.6 (C11), 29.1 (C12, C13 or C14), 29.2 (C12, C13 or C14), 29.4 (C12, C13 or C14), 31.8 (C10), 32.0 (C15), 48.1 (C19), 51.8 (C20), 95.1 (C8), 119.4 (C2), 127.0 (C23), 127.0 (C24), 128.1 (C22), 128.8 (C5), 128.9 (C6), 129.7 (C4), 133.1 (C3), 141.5 (C21), 143.8 (C1), 168.6 (C9), 189.7 (C7); **mp** (15% Et_2O/pet ether 40-60): = 57-58 ^{0} C; **υ** (cm^{-1}) (neat) = 2923 (m, C-H), 2853 (m, C-H), 1591 (s, C=O), 1568 (s, C=C Ar), 1548 (m, C=C Ar); **HRMS:** *m*/*z* (ES) calculated for $C_{31}H_{37}BrNO$ $[M+H]^+$: 518.2059, found 518.2073.

General Procedure 2

To a solution of ynone (1.0 equiv) in MeOH (0.15 M) was added amine (2 equiv) followed by stirring and heating in a sealed tube. The mixture was then cooled and concentrated in vacuo, followed by drying overnight under a stream of nitrogen.

(*Z*)-1-(2-Bromophenyl)-3-(butylamino)undec-2-en-1-one (13aa)

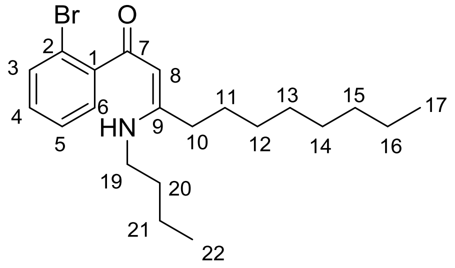

Prepared according to General Procedure 2 for 2 h, using **11a** (0100 mg, 0.311 mmol) and *n*-butyl amine (0.06 mL, 0.62 mmol) as the starting material and stirred at 70 °C for 2 h to give title compound as yellow viscous liquid (121 mg, 0.31 mmol, 99%).

R*f* (20% EtOAc/petroleum ether 40-60) = 0.71; **^{1}H NMR** (400 MHz, $CDCl_3$) δ = 0.90 (3H, t, *J* = 7.0 Hz, H17), 0.99 (3H, t, *J* = 7.3 Hz, H22), 1.26-1.36 (8H, m, H13, H14, H15, H16), 1.37-1.44 (2H, m, H12), 1.49 (2H, sextet, *J* = 7.6 Hz, H21), 1.60 (2H, qn, *J* = 7.6 Hz, H11), 1.68 (2H, qn, *J* = 7.0 Hz, H20), 2.28-2.31 (2H, m, H10), 3.35 (2H, q, *J* = 7.0 Hz, H19), 5.25 (1H, s, H8), 7.18 (1H, td, *J* = 7.9, 1.8 Hz, H5), 7.31 (1H, td, *J* = 7.6, 1.2 Hz, H4), 7.43 (1H, dd, *J* = 7.6, 1.8 Hz, H3), 7.57 (1H, dd, *J* = 7.9, 1.2 Hz, H6), 11.26-11.29 (1H, m, N-H); **^{13}C NMR** (100 MHz, $CDCl_3$): δ = 13.8 (C22), 14.1 (C17), 20.1 (C21), 22.6 (C16), 27.9 (C11), 29.2 (C15), 29.3 (C14), 29.5 (C12), 31.8 (C13), 32.3 (20), 32.2 (C10), 42.8 (C19), 94.7 (C8), 119.5 (C2), 127.0 (C4), 129.1 (C3), 129.7 (C5), 133.2 (C6), 143.8 (C1), 169.1 (C9), 189.4 (C7); **υ** (cm^{-1}) (neat) = 2926 (m, C-H), 2854 (m, C-H), 1595 (s, C=O), 1568 (s, C=C Ar), 1516 (m, C=C Ar); **HRMS:** *m*/*z* (ES) calculated for $C_{21}H_{33}BrNO$ $[M+H]^+$: 394.1746, found 394.1733.

(*Z*)-3-(Allylamino)-1-(2-bromophenyl)undec-2-en-1-one (13ab)

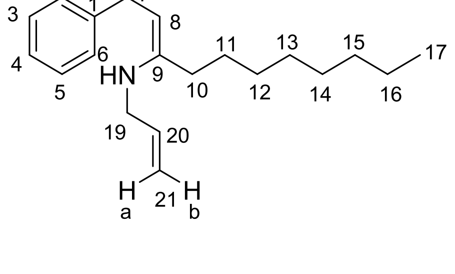

Prepared according to General Procedure 2 for 3.5 h, using **11a** (100 g, 0.31 mmol) and allyl amine (0.05 mL, 0.62 mmol) as the starting material and stirred at 60 °C for 3 h to give title compound as dark orange viscous liquid (118 mg, 0.31 mmol, 100%).

R*f* (20% Et_2O/petroleum ether 40-60) = 0.12; ^{1}**H NMR** (400 MHz, $CDCl_3$)**:** δ = 0.90 (3H, t, *J* = 7.2 Hz, H17), 1.25-1.44 (10H, m, H12, H13, H14, H15, H16), 1.60 (2H, qn, *J* = 7.8 Hz, H11), 2.18-2.32 (2H, m, H10), 3.98-4.02 (2H, m, H19), 5.25 (1H, dd, *J* = 10.6, 1.0 Hz, H21b), 5.31 (1H, s, H8), 5.34 (1H, dd, *J* = 17.7, 1.0 Hz, H21a), 5.90-6.00 (1H, m, H20), 7.20 (1H, td, *J* = 7.8, 1.7 Hz, H5), 7.32 (1H, td, *J* =7.5, 1.0 Hz, H4), 7.45 (1H, dd, *J* = 7.5, 1.7 Hz, H3), 7.58 (1H, dd, *J* = 7.8, 1.0 Hz, H6), 11.30 (1H, m, N-H); ^{13}**C NMR** (100 MHz, $CDCl_3$): δ = 14.1 (C17), 22.6 (C16), 27.9 (C11), 29.2 (C13), 29.3 (C14), 29.45 (C12), 31.8 (C15), 32.0 (C10), 45.2 (C19), 95.3 (C8), 116.8 (C21), 119.5 (C2), 127.1 (C4), 129.0 (C3), 129.8 (C5), 133.2 (C6), 133.7 (C20), 143.7 (C1), 169.2 (C9), 189.9 (C7); **υ** (cm^{-1}) (neat) = 2923 (m, C-H), 2853 (m, C-H), 1592 (s, C=O), 1568 (s, C=C Ar), 1515 (m, C=C Ar); **HRMS:** *m*/*z* (ES) calculated for $C_{20}H_{28}BrNO$ $[M+H]^+$: 377.1354, found 377.1339.

(*Z*)-1-(2-Bromophenyl)-3-(isobutylamino)undec-2-en-1-one (13ac)

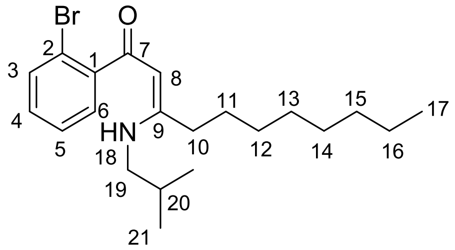

Prepared according to General Procedure 2, using **11a** (0.100 g, 0.311 mmol) and isobutyl amine (0.07 m, 0.622 mmol) as the starting material and stirred at 70 °C for 1 h and 20 min to give title compound as yellow viscous liquid (0.127 g, 0.322 mmol, 100%).

R*f* (20% Et_2O/petroleum ether 40-60) = 0.33; ^{1}**H NMR** (400 MHz, $CDCl_3$)**:** δ = 0.91 (3H, t, *J* = 7.02 Hz, H17), 1.06 (6H, d, *J* = 6.71 Hz, H21), 1.25-1.36 (8H, m, H13, H14, H15, H16), 1.38-1.44 (2H, m, H12), 1.60 (2H, qn, *J* = 7.93 Hz, H11), 1.94 (1H, nonet, *J* = 6.71 Hz, H20), 2.27-2.32 (2H, m, H10), 3.17 (2H, t, *J* = 6.71 Hz, H19), 5.27 (1H, s, H8), 7.19 (1H, td, *J* = 7.93, 1.83 Hz, H5), 7.31 (1H, td, *J* = 7.63, 1.22 Hz, H4), 7.44 (1H, dd, *J* = 7.73, 1.83 Hz, H3), 7.58 (1H, dd, *J* = 7.93, 1.22 Hz, H6), 11.40 (1H, m, H18); ^{13}**C NMR** (100 MHz, $CDCl_3$): δ = 14.10 (C17), 20.27(C21), 22.65 (C16), 27.83 (C11), 29.16 (C14 & C20), 29.29 (C13), 29.47 (C12), 31.81 (C15), 32.18 (C10), 50.63 (C19), 94.81 (C8), 119.49 (C1), 127.01 (C4), 129.08 (C3), 129.67 (C5), 133.15 (C6), 143.81 (C2), 169.21 (C9), 189.36 (C7); **υ** (cm^{-1}) (neat) = 2924 (m,

C-H), 2854 (m, C-H), 1596 (s, C=O), 1569 (s, C=C Ar), 1515 (m, C=C Ar); **HRMS:** *m*/*z* (ES) calculated for $C_{21}H_{33}BrNO$ $[M+H]^+$: 394.1746, found 394.1733.

General procedure 3 (Pd-catalysed cyclisation):

A mixture of secondary amine (1 equiv), Cs_2CO_3 (2 equiv), Pd_2dba_3 (0.043 equiv), $P(2\text{-furyl})_3$ (0.195 equiv) in toluene (0.1 M with respect to amine) were stirred in a sealed tube at 100 °C overnight. The mixture was then filtered through celite, rinsed with EtOAc and concentrated in vacuo. The crude product was purified using silica gel flash chromatography eluting with 70% EtOAc/pet ether 40–60.

1-Butyl-2-octylquinolin-4(1*H*)-one (14aa)

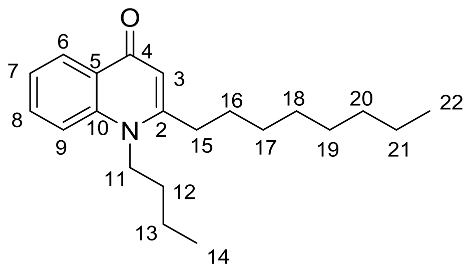

Prepared according to General Procedure 3, using **13aa** (093 mg, 0.24 mmol) as the starting material to give the title compound as a yellow oil (12.7 mg, 0.04 mmol, 17%).

R*f* (50% EtOAc/petroleum ether 40-60) = 0.10; ^{1}**H NMR** (400 MHz, $CDCl_3$): δ = 0.90 (3H, t, *J* = 6.8 Hz, H22), 1.05 (3H, t, *J* = 7.5 Hz, H14), 1.27-1.40 (8H, m, H18, H19, H20, H21), 1.43-1.60 (4H, m, H13, H17), 1.72 (2H, qn, *J* = 7.8 Hz, H16), 1.77-1.85 (2H, m, H12), 2.67-2.73 (2H, m, H15), 4.12-4.17 (2H, m, H11), 6.27 (1H, s, H3), 7.34 (1H, td, *J* = 7.8, 1.0 Hz, H7), 7.47 (1H, d, *J* = 8.5 Hz, H9), 7.64 (1H, td, *J* = 7.2, 1.7 Hz, H8), 8.46 (1H, dd, *J* = 7.8, 1.7 Hz, H6); ^{13}**C NMR** (100 MHz, $CDCl_3$): δ = 13.7 (C14), 14.1 (C22), 20.1 (C13), 22.6 (C21), 29.0 (C16), 29.1 (C17 or C18 or C19), 29.3 (C17 or C18 or C19), 29.4 (C17 or C18 or C19), 31.0 (C12), 31.8 (C20), 34.0 (C15), 46.0 (C11), 111.1 (C3), 115.5 (C9), 123.1 (C7), 126.9 (C5), 127.0 (C6),131.9 (C8), 140.9 (C10), 154.4 (C2), 177.7 (C4); **υ** (cm^{-1}) (neat) = 2925 (m, C-H), 2853 (m, C-H), 1621 (s, C=O quinolone), 1596 (s, C=C Ar), 1551 (m, C=C Ar); **HRMS:** *m*/*z* (ES) calculated for $C_{21}H_{31}NO$ $[M+H]^+$: 313.2406, found 313.2393.

General Procedure 4 (Cu-catalysed cyclisation):

A mixture of secondary amine (1 equiv), CuI (5 mol %), DMEDA (5 mol %) and *t*-BuONa (2 equiv) in DMSO (0.1 M) were stirred at 80 °C in a sealed tube for 2 h. The mixture was cooled to room temperature, diluted with Et_2O and washed with sat. NaCl. The organic

phase was separated, dried ($MgSO_4$) and concentrated in vacuo. The crude product was purified using silica gel chromatography.

1-Benzyl-2-octylquinolin-4(1*H*)-one (14ad)

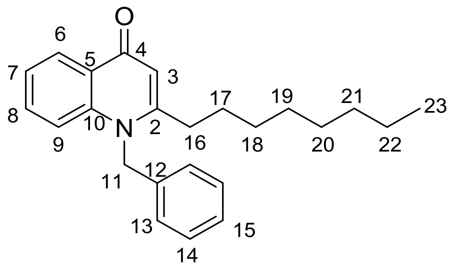

Prepared according to General Procedure 4, using **13ad** (52 mg, 0.117 mmol) as the starting material. The chromatography column was eluted with 70% EtOAc/pet ether 40–60 to give the title compound as a yellow oil (9 mg, 0.026 mmol, 22%).

R*f* (60% EtOAc/petroleum ether 40-60) = 0.13; ^{1}**H NMR** (400 MHz, $CDCl_3$): δ = 0.87 (3H, t, *J* = 6.8 Hz, H23), 1.21-1.31 (6H, m, H20, H21, H22), 1.34-1.42 (2H, m, H19), 1.63-1.73 (4H, m, H17, H18), 2.63-2.68 (2H, m, H16), 5.42 (2H, s, H11), 6.34 (1H, s, H3), 7.04 (2H, d, *J* = 6.8 Hz, H13), 7.27-7.37 (5H, m, H7, H9, H14, H15), 7.49 (1H, td, *J* = 8.5, 1.7 Hz, H8), 8.47 (1H, dd, *J* =8.2, 1.7 Hz, H6); ^{13}**C NMR** (100 MHz, $CDCl_3$): δ = 14.1 (C23), 22.6 (C22), 28.8 (C17, C18), 29.1 (C19), 29.2 (C20), 31.7 (C21), 34.0 (C16), 49.7 (C11), 110.2 (C3), 116.3 (C9), 123.4 (C7), 125.3 (C13), 126.7 (C6), 126.8 (C5), 127.9 (C15), 129.2 (C14), 132.1 (C8), 135.7 (C12), 141.5 (C10), 154.9 (C2), 178.0 (C4); **υ** (cm^{-1}) (neat) = 2924 (m, C-H), 2853 (m, C-H), 1621 (s, C=O quinolone), 1600 (s, C=C Ar), 1552 (m, C=C Ar); **HRMS:** *m*/*z* (ES) calculated for $C_{24}H_{29}NO$ $[M+H]^+$: 347.2249, found 347.2235.

1-(2,2-Diphenylethyl)-2-octylquinolin-4(1*H*)-one (14ae)

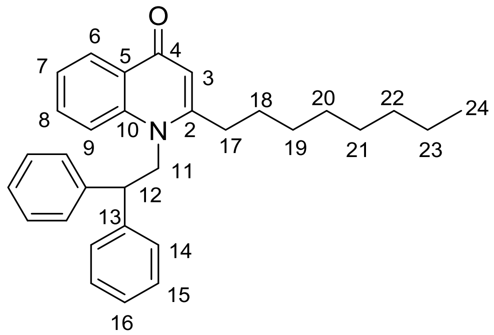

Prepared according to General Procedure 4, using **13ae** (52 mg, 0.096 mmol) as the starting material. The chromatography column was eluted with 50% EtOAc/pet ether 40–60 to give the title compound as a pale yellow oil (5.6 mg, 0.013 mmol, 13%).

R$_f$ (50% EtOAc/petroleum ether 40-60) = 0.19; ^{1}**H NMR** (400 MHz, $CDCl_3$): δ = 0.89 (3H, t, *J* = 7.3 Hz, H24), 1.14-1.51 (10H, m, H19, H20, H21, H22, H23), 1.71-1.83 (2H, m, H18), 4.49 (1H, t, *J* = 7.0 Hz, H12), 4.67-4.85 (2H, m, H11), 6.06 (1H, s, H3), 6.75-6.85 (2H, m, Ar-H), 7.13-7.45 (10H, m, H7, H9, Ar-H), 7.61 (1H, td, *J* = 8.9, 1.8 Hz, H8), 8.52 (1H, dd, *J* = 8.2, 1.8 Hz, H6); ^{13}**C NMR** (100 MHz, $CDCl_3$): δ = 14.1 (C24), 22.6 (C23), 28.1 (C18), 29.1 (C19 or C20 or C21), 29.1 (C19 or C20 or C21), 29.7 (C19 or C20 or C21), 31.8 (C22), 33.4 (C17), 49.0 (C12), 50.7 (C11), 110.8 (C3), 115.9 (C9), 123.3 (C7), 127.2 (C5), 127.3 (C6), 127.5 (C14 or C15 or C16), 128.2 (C14 or C15 or C16), 128.9 (C14 or C15 or C16), 132.1 (C8), 140.7 (C10), 155.6 (C2), 177.7 (C4), weak C13 lost in baseline; **υ** (cm^{-1}) (neat) = 2922 (m, C-H), 2851 (m, C-H), 1622 (s, C=O quinolone), 1597 (s, C=C Ar), 1552 (m, C=C Ar); **HRMS:** *m*/*z* (ES) calculated for $C_{31}H_{36}NO$ $[M+H]^+$: 438.2797, found 438.2804.

2-Octyl-1-(prop-1-en-1-yl)quinolin-4(1*H*)-one (14ab)

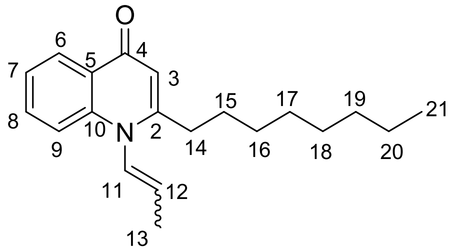

Prepared according to General Procedure 4, using **13ab** (50 mg, 0.132 mmol) as the starting material. The chromatography column was eluted with 70% EtOAc/pet ether 40–60 to give the title compound as an inseparable mixture of *E* and *Z* isomers in ratio of 0.4:1 (calculated by using the ratio of NMR integrals) as a white-yellow solid (10 mg, 0.033 mmol, 26%).

R$_f$ (70% EtOAc/petroleum ether 40-60) = 0.13; ^{1}**H NMR** (400 MHz, $CDCl_3$): δ = 0.90 (3H, t, *J* = 6.8 Hz, H21), 1.23-1.44 (10H, m, H16, H17, H18, H19, H20), 1.50 (3H, dd, *J* = 6.8, 2.0 Hz, H13*Z*), 1.59-1.68 (2H, m, H15), 2.04 (3H, dd, *J* = 6.8, 2.0 Hz, H13*E*), 2.56-2.67 (2H, m, H14), 5.96 (1H, dq, *J* = 6.8, 13.6 Hz, H12*E*), 6.20 (1H, qn, H12*Z*), 6.28 (1H, s, H3*E*), 6.29 (1H, 2, H3*Z*), 6.41 (1H, dq, *J* = 13.9, 1.7 Hz, H11*E*), 6.45 (1H, dq, *J* = 7.2, 1.7 Hz, H11*Z*), 7.37 (1H, t, *J* = 7.2, 1.0 Hz, H7), 7.48 (1H, d, *J* = 8.2 Hz, H9*Z*), 7.53 (1H, d, *J* = 7.8 Hz, H9*E*), 7.57 (1H, td, *J* = 6.8, 1.7 Hz, H8), 8.42 (1H, dd, *J* = 8.2, 1.7 Hz, H6); ^{13}**C NMR** (100 MHz, $CDCl_3$): δ = 12.3 (C13*Z*), 14.1 (C21), 15.1 (13*E*), 22.6 (C20), 28.1 (C15), 29.1 (C17 or C18 or C19), 29.1 (C17 or C18 or C19), 29.7 (C17 or C18 or C19), 31.8 (C16), 33.7 (C14*E*), 34.1 (C14*Z*), 110.2 (C3), 116.7 (C9*Z*), 117.2 (C9*E*), 123.4 (C7), 125.5 (C11*Z*), 125.8 (C11*E*), 125.9 (C5), 126.3 (C6*E* or *Z*), 126.5 (C6*E* or *Z*), 131.5 (C8*E*), 131.8 (C8*Z*), 131.9 (C12*Z*), 134.0 (C12*E*), 140.8 (C10), 154.5 (C2*Z*), 154.8 (C2*E*), 178.1 (C4); **mp** (70% EtOAc/pet ether 40-60) = 51-52 °C; **υ** (cm^{-1}) (neat) = 2915 (m, C-H), 2849 (m, C-H), 1621 (s, C=O quinolone),

1597 (s, C=C Ar), 1575 (m, C=C), 1551 (m, C=C Ar); **HRMS:** *m*/*z* (ES) calculated for $C_{20}H_{27}NO$ $[M+H]^+$: 297.2093, found 297.2094 .

1-Isobutyl-2-octylquinolin-4(1*H*)-one (14ac)

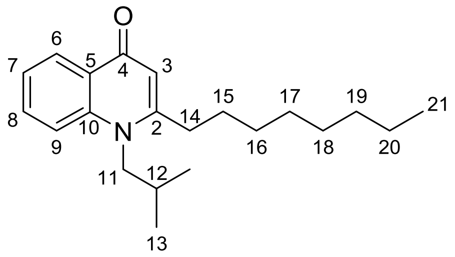

Prepared according to General Procedure 3, using **13ac** (0.095 g, 0.24 mmol) as the starting material to give the title compound as a yellow oil (0.02 g, 0.064 mmol, 26%).

^{1}**H NMR** (500 MHz, $CDCl_3$): δ = 0.83-1.11 (9H, m, H13 & H21), 1.23-1.37 (8H, m. H17, H18, H19 & H20), 1.37-1.44 (2H, m, H16), 1.60-1.71 (2H, br s, H15), 2.28 (1H, app sept, *J* = 6.9 Hz, H12), 2.55-2.86 (2H, m, H14), 3.79-4.21 (2H, m, H11), 6.25 (1H, s, H3), 7.34 (1H, ddd, *J* = 0.6, 7.3, 7.9 Hz, H7), 7.46 (1H, d, *J* = 8.7 Hz, H9), 7.62 (1H, ddd, *J* = 1.8, 7.0, 8.8 Hz, H8), 8.46 (1H, dd, *J* = 1.7, 8.1 Hz, H6); ^{13}**C NMR** (125 MHz, $CDCl_3$): δ = 14.1 (C21), 19.9 (C13), 22.6 (C20), 28.1 (C12), 28.8 (C15), 29.1 (C17/C18/C19), 29.3 (C17/C18/C19), 29.3 (C16), 31.8 (C14), 34.7 (C11), 111.3 (C3), 116.2 (C9), 123.1 (C7), 127.0, (C6), 127.1 (C10), 131.6 (C8), 141.2 (C5), 155.2 (C2); **ʋ** (cm^{-1}) (neat) = 2925 (m, C-H), 2853 (m, C-H), 1623 (s, C=O quinolone), 1597 (s, C=C Ar), 1552 (m, C=C Ar); **HRMS:** *m*/*z* (ES) calculated for $C_{21}H_{31}NO$ $[M+H]^+$: 313.2406, found 313.2394.

5-Bromo-2-methylpent-2-ene

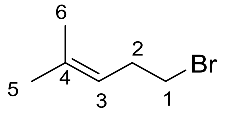

To a solution of methylmagnesium bromide (3.0 M in Et_2O, 31 mL, 97 mmol) in THF (30 mL) at 0 °C was added dropwise a solution of cyclopropyl methyl ketone (6.87 g, 80 mmol) in THF (12 mL). The mixture was heated under reflux for 20 min, cooled to room temperature then slowly added to a cooled solution of concentrated H_2SO_4 in water (60 mL, 1:2) at a steady rate so that the temperature did not rise above 10 °C. The resulting mixture was stirred for 30 min at 0 °C then the organic layer was separated. The aqueous phase was extracted with Et_2O (3 × 50 mL) and the organic phases combined, washed with sat.

NaHCO$_3$ (100 mL) and brine (100 mL) and dried (MgSO$_4$). The solvent was removed in vacuo to give the product as a yellow liquid (9.115 g, 55.89 mmol, 70%).

^{1}**H NMR** (400 MHz, CDCl$_3$): δ = 1.62 (3H, 2, H6), 1.70 (3H, s, H5), 2.55 (2H, q, *J* = 7.2 Hz, H2), 3.34 (2H, t, *J* = 7.5 Hz, H1), 5.13 (1H, t, *J* = 7.2 Hz, H3); ^{13}**C NMR** (100 MHz, CDCl$_3$): δ =17.9 (H6), 25.7 (H5), 31.8 (H1), 32.9 (H2), 121.0 (H3), 135.0 (H4); **υ** (cm^{-1}) (neat) = 2968 (m, C-H), 2916 (m, C-H), 1671 (w, C=C).

Analytical data consistent with the literature [3].

6-Methylhept-5-en-1-yne (10b)

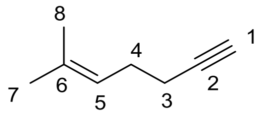

To a cold (10 °C) suspension of lithium acetylide ethylenediamine complex (4.54 g, 51.4 mmol) in DMSO (24 mL) was added 5-bromo-2-methylpent-2-ene dropwise with vigorous stirring over 30 m. The reaction mixture was then allowed to warm to room temperature over a period of 1 h, maintained at room temperature for 1 more h, then cooled to 10 °C. The mixture was quenched by slow addition of water (20 mL), extracted with pet ether 30–40 (4 × 40 mL) and dried (MgSO$_4$). Some of the solvent was removed in vacuo to give the product as a colorless solution of alkyne in pet ether 30–40 of ratio alkyne/pet ether of 1:2.8, calculated using NMR integrals (7.11 g, 23 mmol, 47%).

^{1}**H NMR** (400 MHz, CDCl$_3$): δ = 1.65 (3H, s, H8), 1.73 (3H, s, H7), 1.96 (1H, t, *J* = 2.5 Hz, H1), 2.19-2.27 (4H, m, H3, H4), 5.17-5.22 (1H, m, H5); **υ** (cm^{-1}) (neat) = 3309 (m, C≡C-**H**), 2916 (m, C-H), 2859 (m, C-H), 1643 (m, C=C).

Analytical data consistent with the literature [3].

1-(2-Bromophenyl)-7-methyloct-6-en-2-yn-1-one (11b)

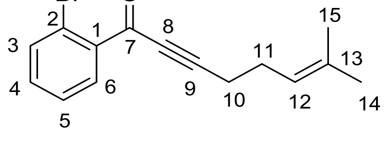

A mixture of 2-bromobenzoyl chloride (3.8 mL, 27.9 mmol), PdCl$_2$(PPh$_3$)$_2$ (0.40 g, 0.55 mmol) and triethylamine (3.80 mL, 27.9 mmol) in THF (155 mL) was stirred for 15 min. CuI

(0.11 g, 1 mmol) was added and the mixture stirred 10 more min, then **10b** in pet ether 30–40 (7.11 g, 23.2 mmol – calculated using the concentration of the solution estimated from the ratio of the NMR integrals of alkyne:pet ether peaks) was added followed by stirring for 2 h at room temperature. The mixture was then diluted with EtOAc (45 mL), washed with sat. aq. NH_4Cl (100 mL), dried ($MgSO_4$) and concentrated in vacuo. The crude product was purified using silica gel flash chromatography, eluting with 5% ether/pet ether 40–60 to give the product as a red/orange liquid (2.54 g, 8.73 mmol, 38%).

R*f* (5% Et_2O/petroleum ether 40-60) = 0.19; **^{1}H NMR** (400 MHz, $CDCl_3$): δ = 1.66 (3H, s, H15), 1.74 (3H, s, H14), 2.35 (2H, q, *J* = 7.0 Hz, H11), 2.51 (2H, t, *J* = 7 Hz, H10), 5.19-5.23 (1H, m, H12), 7.37 (1H, td, *J* = 7.9, 1.8 Hz, H5), 7.43 (1H, td, *J* = 7.6, 1.2 Hz, H4), 7.69 (1H, dd, *J* = 7.9, 1.2 Hz, H6), 8.03 (1H, dd, *J* = 7.6, 1.8 Hz, H3); **^{13}C NMR** (100 MHz, $CDCl_3$): δ = 17.8 (C15), 19.9 (C10), 25.7 (C14), 26.3 (C11), 80.6 (C8), 97.8 (C9), 121.0 (C2), 121.9 (C12), 127.2 (C4), 132.9 (C3), 133.1 (C5), 134.0 (C13), 134.9 (C6), 137.4 (C1), 177.5 (C7); **υ** (cm^{-1}) (neat) = 2916 (w, C-H), 2203 (m, C≡C), 1651 (s, C=O), 1585 (m, C=C Ar), 1563 (w, C=C Ar); **HRMS:** *m*/*z* (ES) calculated for $C_{15}H_{16}BrO$ $[M+H]^+$: 291.0379, found 291.0368.

(*Z*)-1-(2-Bromophenyl)-7-methyl-3-(methylamino)octa-2,6-dien-1-one (13bf)

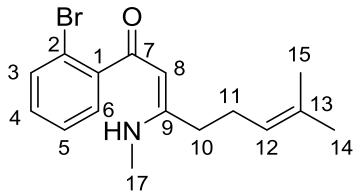

Prepared according to General Procedure 2 for 2 h, using **11b** (100 g, 0.35 mmol) and methylamine in 2 M methanol (0.35 mL, 0.7 mmol) as the starting material and stirred at 50 °C for 2 h to give title compound as yellow a viscous oil (110 mg, 0.34 mmol, 97%).

R*f* (70% EtOAc/petroleum ether 40-60) = 0.59; **^{1}H NMR** (400 MHz, $CDCl_3$): δ = 1.65 (3H, s, H15), 1.73 (3H, s, H14), 2.27-2.37 (4H, m, H10, H11), 3.07 (3H, d, *J* = 5.5 Hz, H17), 5.16-5.20 (1H, m, H12), 5.29 (1H, s, H8), 7.20 (1H, td, *J* = 7.8, 1.7 Hz, H4), 7.32 (1H, td, *J* = 7.5, 1.0 Hz, H5), 7.41 (1H, dd, *J* = 7.5, 1.7 Hz, H6), 7.58 (1H, dd, *J* = 7.8, 1.0 Hz, H3), 11.21 (1H, s, N-H); **^{13}C NMR** (100 MHz, $CDCl_3$): δ = 17.8 (C15), 25.7 (C14), 26.1 (C11), 29.5 (C17), 32.0 (C10), 94.9 (C8), 119.5 (C2), 122.13 (C12), 127.1 (C5), 129.0 (C6), 129.7 (C4), 133.2 (C3), 133.5 (C13), 143.8 (C1), 169.7 (C9), 189.7 (C7); **υ (cm^{-1}) (neat) =** 2917 (w, C-H), 1598 (s, C=O), 1568 (s, C=C Ar), 1521 (s, C=C Ar); **HRMS:** *m*/*z* (ES) calculated for $C_{16}H_{21}BrNO$ $[M+H]^+$: 322.0801, found 322.0794.

(*Z*)-1-(2-Bromophenyl)-3-(butylamino)-7-methylocta-2,6-dien-1-one (13ba)

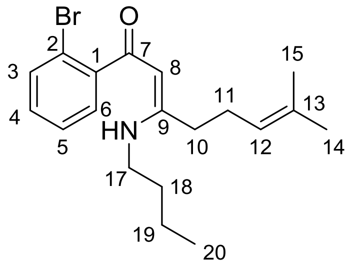

Prepared according to General Procedure 2 for 2 h, using **11b** (100 mg, 0.35 mmol) and *n*-butylamine (0.07 mL, 0.70 mmol) as the starting material and stirred at 50 °C for 2 h to give title compound as a yellow viscous oil (117 mg, 0.32 mmol, 92%).

R_f (70% EtOAc/petroleum ether 40-60) = 0.64; **^{1}H NMR** (400 MHz, $CDCl_3$): δ = 0.99 (3H, t, *J* =7.3 Hz, H20), 1.49 (2H, sextet, *J* = 7.3 Hz, H19), 1.65 (3H, s, H15), 1.69 (2H, qn *J* = 7.3 Hz, H18), 1.73 (3H, s, H14), 2.27-2.36 (4H, m, H10, H11), 3.36 (2H, q, *J* = 7.0 Hz, H17), 5.16-5.20 (1H, m, H12), 5.27 (1H, s, H8), 7.19 (1H, td, *J* = 7.9, 1.8 Hz, H4), 7.31 (1H, td, *J* = 7.6, 1.2 Hz, H5), 7.43 (1H, dd, *J* = 7.6, 1.8 Hz, H6), 7.58 (1H, dd, *J* = 7.9, 0.9 Hz, H3), 11.28 (1H, s, N-H); **^{13}C NMR** (100 MHz, $CDCl_3$): δ = 13.8 (C20), 17.7 (C15), 20.1 (C19), 25.7 (C14), 26.5 (C11), 32.1 (C10 or C18), 32.2 (C10 or C18), 42.8 (C17), 94.7 (C8), 119.5 (C2), 122.2 (C12), 127.0 (C5), 129.0 (C6), 129.7 (C4), 133.2 (C3), 133.4 (C13), 143.8 (C1), 168.6 (C9), 189.4 (C7); **υ** (cm^{-1}) (neat) = 2927 (m, C-H), 1595 (s, C=O), 1567 (s, C=C Ar), 1516 (m, C=C Ar); **HRMS:** *m/z* (ES) calculated for $C_{19}H_{27}BrNO$ $[M+H]^+$: 364.1276, found 364.1258

(*Z*)-1-(2-Bromophenyl)-3-(isobutylamino)-7-methylocta-2,6-dien-1-one (13bc)

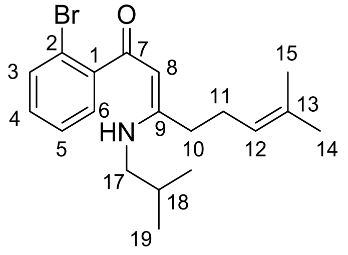

Prepared according to General Procedure 2 for 2 h, using **11b** (100 g, 0.35 mmol) and isobutylamine (0.07 mL, 0.70 mmol) as the starting material and stirred at 50 °C for 2 h to give title compound as a yellow viscous oil (122 mg, 0.35 mmol, 100%).

R_f (70% EtOAc/petroleum ether 40-60) = 0.70; **^{1}H NMR** (400 MHz, $CDCl_3$): δ = 1.06 (6H, d, *J* = 6.8 Hz, H19), 1.65 (3H, s, H15), 1.73 (3H, s, H14), 1.95 (1H, nonet, *J* = 6.8 Hz, H18), 2.26-2.36 (4H, m, H10, H11), 3.19 (2H, t, *J* = 6.5 Hz, H17), 5.15-5.20 (1H, m, H12), 5.29 (1H, s,

H8), 7.19 (1H, td, *J* = 7.8, 1.7 Hz, H4), 7.32 (1H, td, *J* = 7.5, 1.0 Hz, H5), 7.44 (1H, dd, *J* = 7.5, 1.7 Hz, H6), 7.58 (1H, d, *J* 8.2 Hz, H3), 11.41 (1H, s, N-H); **^{13}C NMR** (100 MHz, $CDCl_3$): δ = 17.8 (C15), 20.3 (C19), 25.7 (C14), 26.5 (C11), 29.1 (C18), 32.2 (C10), 50.7 (C17), 94.8 (C8), 119.5 (C2), 122.2 (C12), 127.0 (C5), 129.0 (C6), 129.7 (C4), 133.2 (C3), 133.4 (C13), 143.8 (C1), 168.7 (C9), 189.4 (C7); **υ** (cm^{-1}) (neat) = 2928 (w, C-H), 1595 (s, C=O), 1568 (s, C=C Ar), 1515 (m, C=C Ar); **HRMS:** *m*/*z* (ES) calculated for $C_{19}H_{27}BrNO$ $[M+H]^+$: 364.1276, found 364.1280.

(Z)-3-(Allylamino)-1-(2-bromophenyl)-7-methylocta-2,6-dien-1-one (13bb)

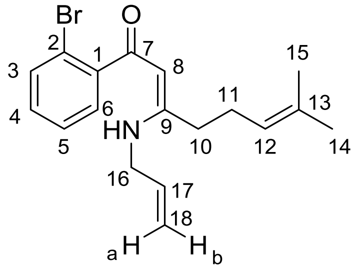

Prepared according to General Procedure 2, using **11b** (0.1 g, 0.35 mmol) and allylamine (0.057 mL, 0.7 mmol) as the starting material and stirred at 50 °C for 2 h to give title compound as orange oil (0.117 g, 0.34 mmol, 97%).

R_f (70% EtOAc/petroleum ether 40-60) = 0.63; **^{1}H NMR** (400 MHz, $CDCl_3$): δ = 1.62 (3H, s, H15), 1.70 (3H, s, H14), 2.25-2.34 (4H, m, H10 & H11), 3.97-4.01 (2H, m, H16), 5.12-5.17 (1H, m, H12), 5.24 (1H, tdd, *J* = 1.7, 1.0, 10.4 Hz, H18b), 5.31 (1H, s, H8) 5.31 (1H, tdd, *J* = 1.8, 1.1, 17.1 Hz, H18a), 5.93 (1H, dqn, *J* = 17.2, 5.11 Hz, H17), 7.16-7.20 (1H, m, H4), 7.30 (1H, td, *J* = 7.5, 1.1 Hz, H5), 7.41 (1H, dd, *J* = 1.7, 7.5 Hz, H6), 7.56 (1H, dd, *J* = 1.0, 8.0 Hz, H3), 11.28 (1H, br s, N-H); **^{13}C NMR** (100 MHz, $CDCl_3$): δ = 17.8 (C15), 25.7 (C14), 26.6 (C11), 32.0 (C10), 45.3 (C16), 95.3 (C8), 116.8 (C18), 119.5 (C2), 122.2 (C12), 127.1 (C5), 129.0 (C6), 129.8 (C4), 133.2 (C3), 133.5 (C13), 133.7 (C17), 143.6 (C1), 168.7 (C9), 189.9 (C7); **υ** (cm^{-1}) (neat) = 2916 (w, C-H), 1589 (s, C=O), 1566 (s, C=C Ar), 1520 (m, C=C Ar); **HRMS:** *m*/*z* (ES) calculated for $C_{18}H_{23}BrNO$ $[M+H]^+$: 348.0958, found 348.0953.

1-Methyl-2-(4-methylpent-3-en-1-yl)quinolin-4(1*H*)-one (14bf)

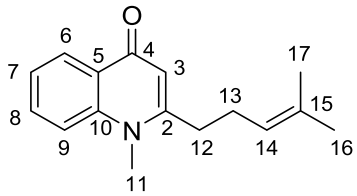

Prepared according to General Procedure 3, using **13bf** (110 mg, 0.34 mmol) as the starting material to give the title compound as a white orange solid (49 mg, 0.21 mmol, 62%).

R*f* (70% EtOAc/petroleum ether 40-60) = 0.11; ^{1}**H NMR** (400 MHz, $CDCl_3$): δ = 1.61 (3H, s, H17), 1.71 (3H, s, H16), 2.38 (2H, q, *J* = 7.8 Hz, H13), 2.71-2.76 (2H, m, H12), 3.75 (3H, s, H11), 5.15 -5.20 (1H, m, H14), 6.24 (1H, s, H3), 7.37 (1H, td, *J* = 7.2, 1.0 Hz, H7), 7.50 (1H, d, *J* = 8.5 Hz, H9), 7.65 (1H, td, *J* = 8.51, 1.7 Hz, H8), 8.44 (1H, dd, *J* = 8.2, 1.4 Hz, H6); ^{13}**C NMR** (100 MHz, $CDCl_3$): δ = 17.8 (C17), 25.7 (C16), 27.1 (C13), 34.1 (C11), 34.8 (C12), 111.2(C3), 115.3 (C9), 121.6 (C14), 123.3 (C7), 126.6 (C5), 126.7 (C6), 132.0 (C8), 134.0 (C15), 141.9 (C10), 154.2 (C2), 177.8 (C4); **mp** (70% EtOAc/pet ether 40-60) = 106-107 ^{0} C; **υ** (cm^{-1}) (neat) = 2923 (w, C-H), 1621 (s, C=O quinolone), 1594 (s, C=C Ar), 1566 (s, C=C Ar); **HRMS:** *m*/*z* (ES) calculated for $C_{16}H_{20}NO$ $[M+H]^+$: 242.1539, found 242.1535.

1-Butyl-2-(4-methylpent-3-en-1-yl)quinolin-4(1*H*)-one (14ba)

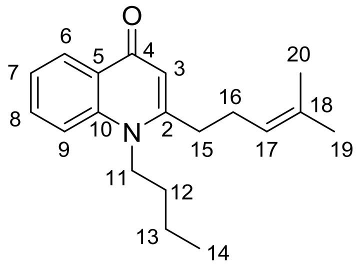

Prepared according to General Procedure 3, using **13ba** (117 mg, 0.32 mmol) as the starting material to give the title compound as a yellow oil (31 mg, 0.11 mmol, 34%).

R*f* (70% EtOAc/petroleum ether 40-60) = 0.09; ^{1}**H NMR** (400 MHz, $CDCl_3$): δ = 1.02 (3H, t, *J* = 7.2 Hz, H14), 1.49 (2H, sextet, *J* = 7.5 Hz, H3), 1.61 (3H, s, H20), 1.71 (3H, s, H19), 1.73-1.82 (2H, m, H12), 2.39 (2H, q, *J* = 7.8 Hz, H16), 2.67-2.72 (2H, m, H15), 4.11-4.16 (2H, m, H11), 5.16-5.21 (1H, m, H17), 6.25 (1H, s, H3), 7.34 (1H, td, *J* = 7.2, 1.0 Hz, H7), 7.47 (1H, d, *J* = 8.5 Hz, H9), 7.63 (1H, td, *J* = 6.8, 1.7 Hz, H8), 6.45 (1H, dd, *J* = 8.2, 1.7 Hz, H6); ^{13}**C NMR** (100 MHz, $CDCl_3$): δ = 13.7 (C14), 17.8 (C20), 20.1 (C13), 25.7 (C19), 27.5 (C16), 31.0 (C12), 34.0 (C15), 46.0 (C11), 111.1 (C3), 115.5 (C9), 121.7 (C17), 123.1 (C7), 126.9 (C5 or C6), 126.9 (C5 or C6), 131.9 (C8), 134.1 (C18), 140.9 (C10), 154.0 (C2), 177.7 (C4);

υ (cm^{-1}) (neat) = 2928 (w, C-H), 1620 (s, C=O quinolone), 1596 (s, C=C Ar), 1571 (m, C=C Ar); **HRMS:** *m*/*z* (ES) calculated for $C_{19}H_{25}NO$ [M+H]$^+$: 284.2009, found 284.2006.

1-Isobutyl-2-(4-methylpent-3-en-1-yl)quinolin-4(1*H*)-one (14bc)

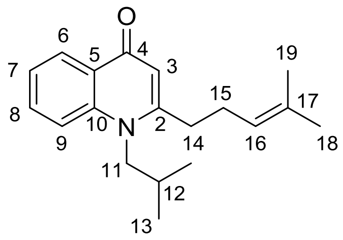

Prepared according to General Procedure 3, using **13bc** (120 g, 0.35 mmol) as the starting material, to give the title compound as a dark yellow oil (34.8 mg, 0.12 mmol, 35%).

R*f* (70% EtOAc/petroleum ether 40-60) = 0.09; ^{1}**H NMR** (400 MHz, $CDCl_3$): δ = 0.85-1.08 (6H, m, H13), 1.60 (3H, s, H19), 1.71 (3H, s, H18), 2.28 (1H, nonet, H12), 2.69-2.89 (2H, m, H14), 4.02-4.24 (2H, m, H11), 5.15-5.20 (1H, m, H16), 6.27 (1H, s, H3), 7.35 (1H, td, *J* = 7.9, 0.6 Hz, H7), 7.47 (1H, d, *J* = 8.5 Hz, H9), 7.65 (1H, td, *J* = 8.5, 1.5 Hz, H8), 8.47 (1H, dd, *J* = 8.2, 1.5 Hz, H6); ^{13}**C NMR** (100 MHz, $CDCl_3$): δ = 17.8 (H19), 25.7 (H18), 27.5 (H13), 28.1 (H12), 34.8 (H14), 52.4 (H11), 111.3 (H3), 116.2 (H9), 121.7 (H16), 123.2 (H7), 126.9 (H6), 127.0 (H5), 131.7 (H17), 141.1 (H10), 154.6 (H2), 177.7 (H4); **υ** (cm^{-1}) (neat) = 2926 (w, C-H), 1621 (s, C=O quinolone), 1596 (s, C=C Ar), 1551 (m, C=C Ar); **HRMS:** *m*/*z* (ES) calculated for $C_{19}H_{26}NO$ [M+H]$^+$: 284.2009, found 284.2007.

2-(4-Methylpent-3-en-1-yl)-1-(prop-1-en-1-yl)quinolin-4(1*H*)-one (14bb) and 1-(2-(dimethylamino)ethyl)-2-(4-methylpent-3-en-1-yl)quinolin-4(1*H*)-one (14bg)

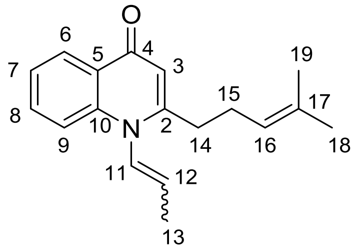

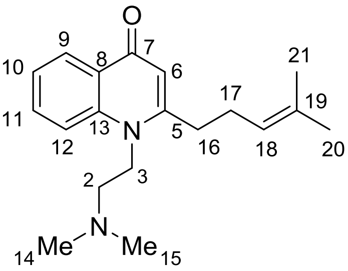

Prepared according to General Procedure 4, using **95** (0.117 g, 0.34 mmol) as the starting material, but with an excess of DMEDA used (3.65 mL, 33.9 mmol). The chromatography column was eluted with 70% EtOAc/pet ether 40-60 to **14bb** as a yellow, solid and inseparable mixture of *E* and *Z* isomers in ratio of 15:85 (calculated by using the ratio of

NMR integrals (47 mg, 0.176 mmol, 52%), and **14bg** as a yellow solid (7 mg, 0.023 mmol, 7%).

*Data for 2-(4-methylpent-3-en-1-yl)-1-(prop-1-en-1-yl)quinolin-4(1H)-one (**14bb**)*

R_f (70% EtOAc/petroleum ether 40-60) = 0.14; ^{1}**H NMR** (500 MHz, $CDCl_3$): δ = 1.47 (3H, dd, *J* = 1.9, 6.9 Hz, H13), 1.58 (3H, s, H17), 1.68 (3H, s, H18), 2.25-2.32 (2H, m H15), 2.52-2.64 (2H, m, H14), 5.12 (1H, tt, *J* = 1.2, 7.1 Hz, H16), 6.0 (1H, dq, *J* = 13.8 Hz, H12*E*), 6.20 (1H, q, *J* = 7.0 Hz, H12*Z*), 6.24 (1H, s, H3*E*), 6.28 (1H, s, H3*Z*), 6.38 (1H, dq, *J* = 13.8, 1.7 Hz, H11*E*), 6.44 (1H, dq, *J* = 7.7, 1.7 Hz, H11*Z*), 7.29-7.35 (1H, m, H7), 7.45 (1H, d, *J* = 8.0 Hz, H9*Z*), 7.49 (1H, d, *J* = 8.9 Hz, H9*E*), 7.52-7.57 (1H, m, H8), 8.38 (1H, dd, *J* = 1.4, 8.3 Hz, H6*E*), 8.41 (1H, dd, *J* = 1.4, 7.9 Hz, H6*Z*); ^{13}**C NMR** (125 MHz, $CDCl_3$): δ = 12.3 (C13), 17.8 (C17), 25.7 (C18), 26.8 (C15), 33.8 (C14), 110.2 (C3), 116.7 (C9*Z*), 117.2 (C9*E*), 122.0 (C16), 123.5 (C7), 125.5 (C11*Z*), 125.9 (C11*E* & C10), 126.4 (C6), 131.9 (C12*Z*), 131.9 (C8), 133.7 (C19), 134.2 (C12*E*), 140.7 (C2*Z*), 150.0 (C2*E*), 154.0 (C5*Z*), 154.3 (C5*E*), 178.1 (C4); **mp:** 101-102 ^{0} C; **υ** (cm^{-1}) (neat) = 2926 (w, C-H), 1621 (s, C=O quinolone), 1596 (s, C=C Ar), 1571 (s, C=C Ar), 1551 (m, C=C Ar); **HRMS:** *m*/*z* (ES) calculated for $C_{18}H_{22}NO$ $[M+H]^+$: 268.1701, found 268.1714.

*Data for 1-(2-(dimethylamino)ethyl)-2-(4-methylpent-3-en-1-yl)quinolin-4(1H)-one (**14bg**)*

^{1}**H NMR** (500 MHz, $CDCl_3$): δ = 1.62 (3H, s, H21), 1.71 (3H, s, H20), 2.35 (6H, s, H14 & H15), 2.41 (2H, q, *J* = 7.4 Hz, H17), 2.65 (2H, t, *J* = 6.9 Hz, H2), 2.75 (2H, t, *J* = 8.0 Hz, H16), 4.29 (3H, t, *J* = 7.7 Hz, H3), 5.16-5.21 (1H, m, H18), 6.27 (1H, s, H6), 7.36 (1H, ddd, *J* = 0.6, 7.3, 7.9 Hz, H10), 7.53 (1H, d, *J* = 8.7 Hz, H12), 7.66 (1H, ddd, *J* = 1.7, 6.9, 8.7 Hz, H11), 8.45 (1H, dd, *J* = 1.7, 8.1 Hz, H9); ^{13}**C NMR** (125 MHz, $CDCl_3$): δ = 17.9 (C21), 25.7 (C20), 27.5 (C17), 34.0 (C16), 44.8 (C3), 45.9 (C14 &C15), 57.3 (C2), 111.2 (C6), 115.4 (C12), 121.6 (C18), 123.3 (C10), 126.9 (C13), 127.0 (C9), 132.1 (C11), 134.1 (C19), 141.0 (C8), 154.1 (C5), 177.8 (C7); **υ** (cm^{-1}) (neat) = 2933 (w, C-H), 1620 (s, C=O), 1597 (s, C=C Ar), 1573 (m, C=C Ar), 1552 (m, C=C Ar); **HRMS:** *m*/*z* (ES) calculated for $C_{19}H_{27}N_2O$ $[M+H]^+$: 299.2123, found 299.2138.

Biological methods

Growth assay:

Each compound was added to individual wells of three 96-well plates (Nunc™ Thermo Fisher Scientific) in 2 µL aliquots in triplicate. Overnight cultures of *Pseudomonas aeruginosa* PAO1, *Escherichia coli* ESS and *Staphylococcus aureus* 25923 were grown in Luria Broth (LB) at 37 ^{o}C and were subsequently inoculated into fresh LB to an initial OD_{600}

of 0.05. The inoculated broth was then added to the 96 well plate in 200 µL aliquots per well to achieve a final concentration of 200 µM of each compound; one strain of bacteria was tested per plate. A gas permeable film was used to seal the plates (4titude) and they were then incubated (37 °C, 200 rpm) in a FLUOstar Omega Microplate Reader (BMG LABTECH) where the optical density (OD_{600}) was recorded at set intervals for 24 hours. A negative control was used which consisted of 2 µL DMSO in 200 µL bacterial medium, and the positive control used consisted of 2 µL gentamicin (10 µg/mL) in 200 µL bacterial medium. A blank control containing only 200 µL LB medium was also used; all controls were used in triplicate. Optical density readings were used to plot graphs showing the relative bacterial growth over time.

Pyocyanin assay:

Method adapted from Frank and DeMoss [4]. An overnight culture of PAO1 was grown in Luria Broth (LB) at 37 °C and subsequently inoculated into each conical flask containing 6 mL fresh LB to an initial OD_{600} of 0.05. A 60 µL aliquot of compound was added to each flask and the cultures were grown in a shaking water bath for 8 hours (37 °C, 200 rpm). A negative control consisting of 60 µL DMSO in 6 mL inoculated broth and an additional control of 6 mL inoculated broth without any additions was used. Following growth, the OD_{600} of each culture was measured using a BioSpectrometer (Eppendorf) and the samples were centrifuged to remove cells (3200*g*, 10 minutes, 20 °C) (Eppendorf centrifuge 5810 R). The supernatant was then removed and mixed with 3 mL chloroform by vortexing to extract pyocyanin. The phases were subsequently separated by centrifugation (3200*g*, 10 minutes, 20 °C) and the chloroform phase was transferred to a fresh tube. Following further extraction with 1 mL HCl (0.2 M), the phases were once again separated by centrifugation (3200*g*, 10 minutes, 20 °C) and the absorbance of the acid phase was measured at 520 nm (Eppendorf BioSpectrometer).

Growth assay data for *P. aeruginosa* PA01

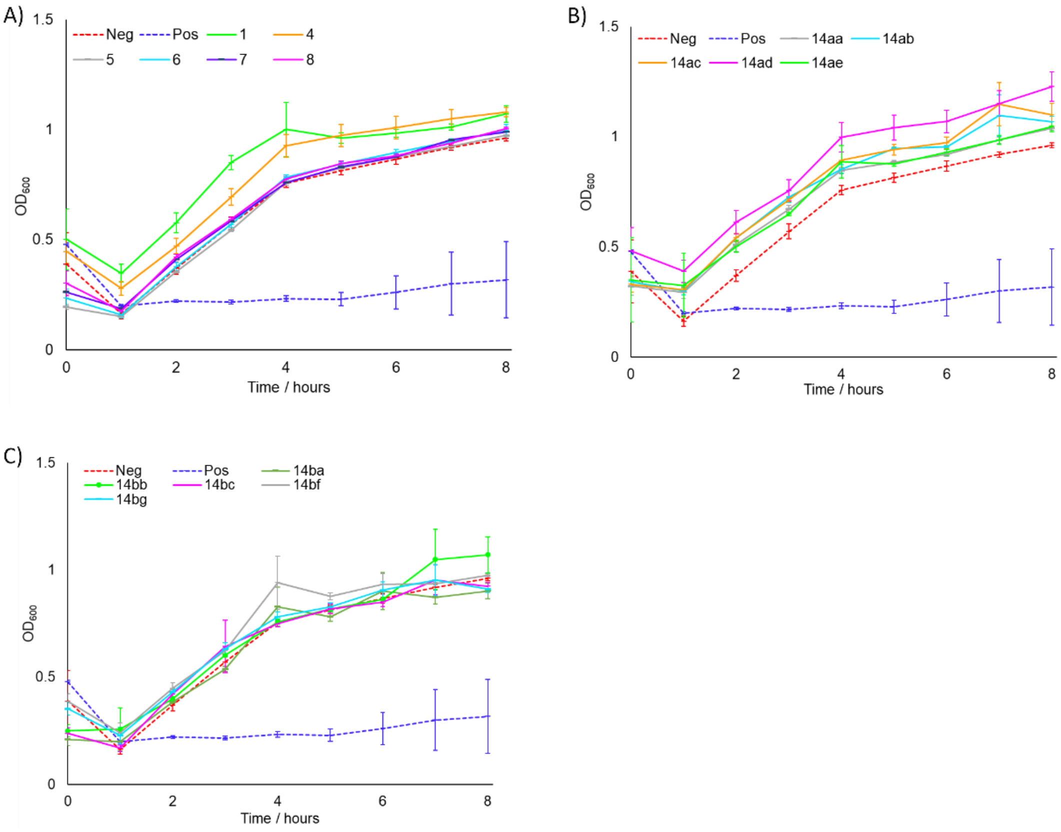

Figure S1: Growth of *P. aeruginosa* PA01 with time in the presence of 200 μM of each compound. A) Natural product series. B) Saturated analogue series. C) Truncated analogue series. Neg = negative control (DMSO blank), Pos = positive control (gentamicin).

References

[1] A. F. Burchat, J. M. Chong and N. Nielsen, *J. Organomet. Chem.*, 1997, **542**, 281–283.

[2] H. Sashida, *Synthesis*, 1998, **1998**, 745–748.

[3] F. Salvaggio, J. T. Hodgkinson, L. Carro, S. M. Geddis, W. R. J. D. Galloway, M. Welch and D. R. Spring, *European J. Org. Chem.*, 2016, **2016**, 434–437.

[4] L. H. Frank and R. D. DeMoss, *J. Bacteriol.*, 1959, **77**, 776–82.

^{1}H and ^{13}C NMR spectra

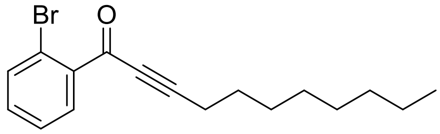

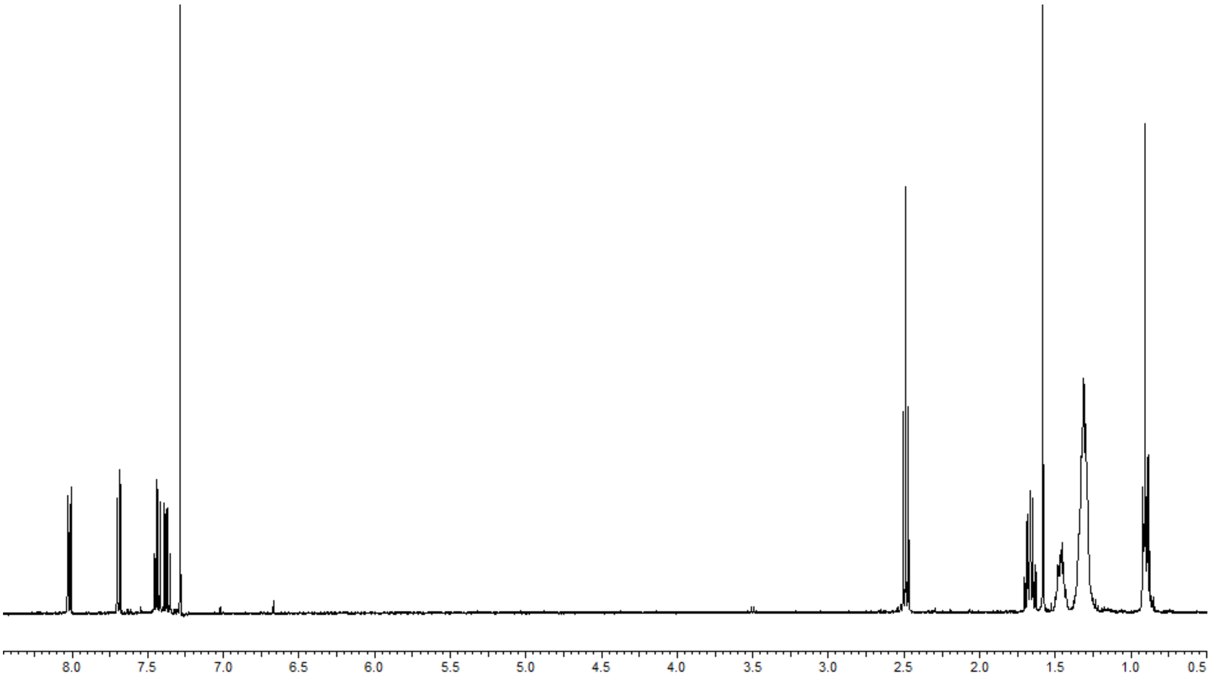

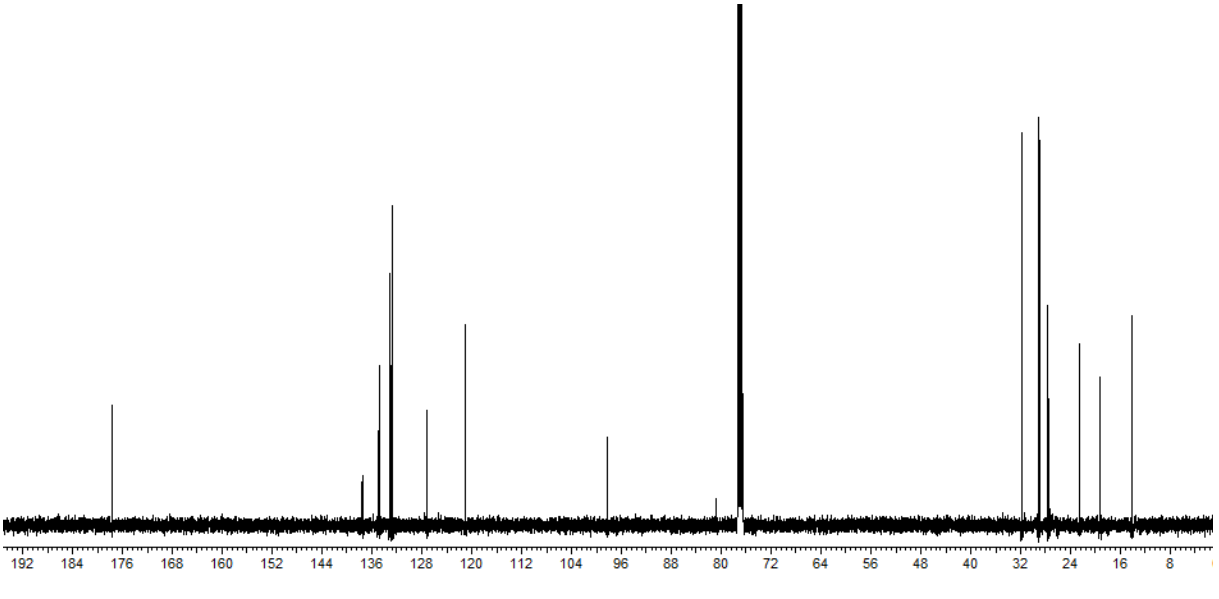

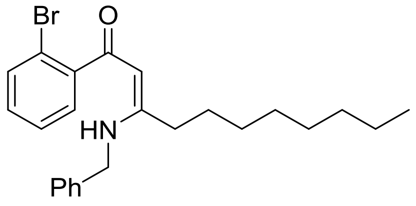

Br
O
HN
Ph

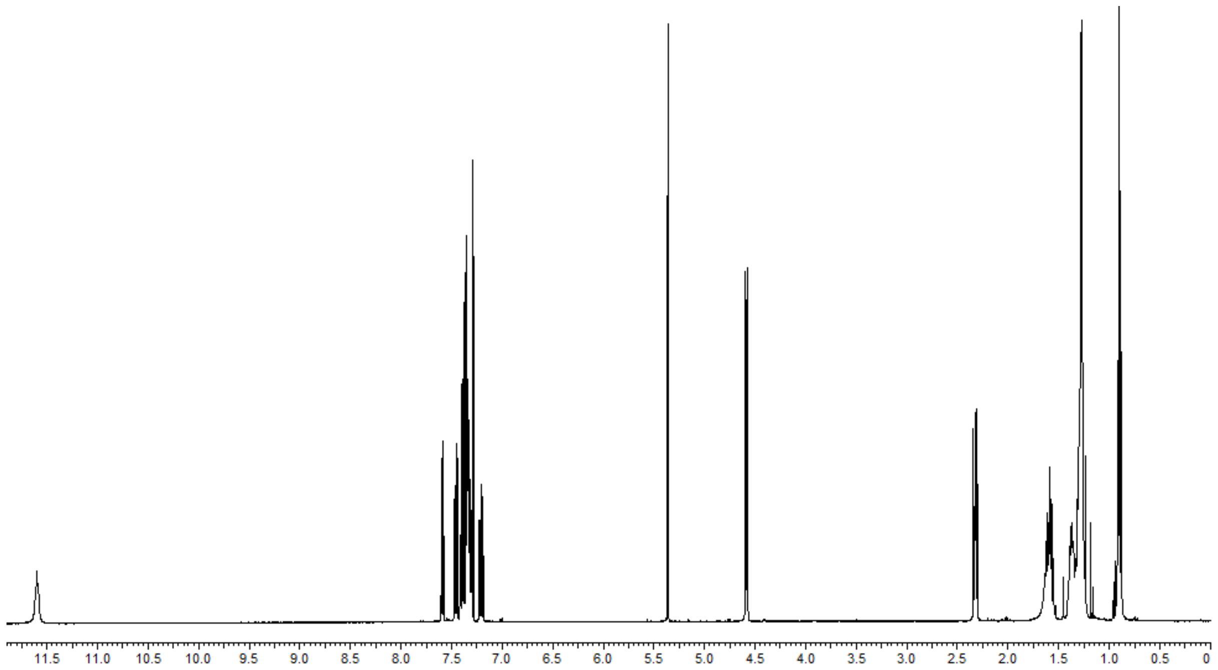

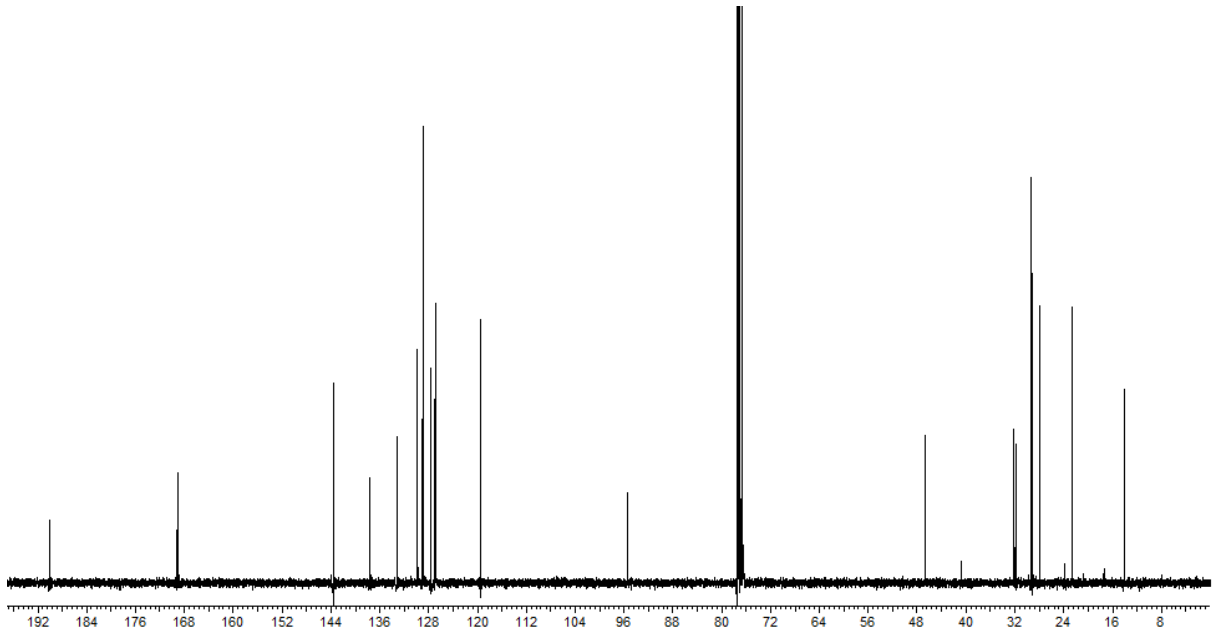

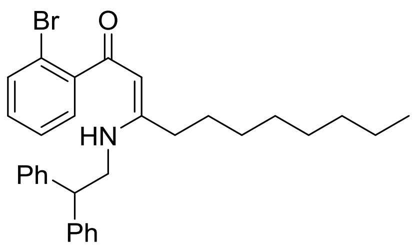
Br
O
HN
Ph
Ph

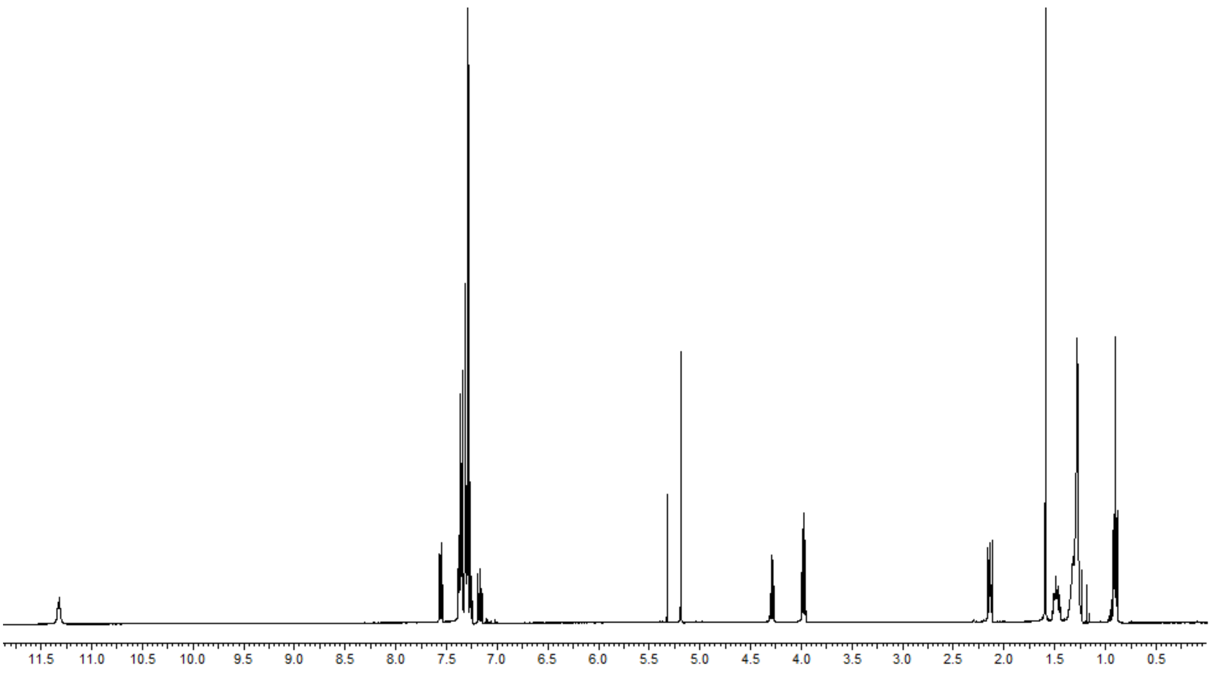
11.5 11.0 10.5 10.0 9.5 9.0 8.5 8.0 7.5 7.0 6.5 6.0 5.5 5.0 4.5 4.0 3.5 3.0 2.5 2.0 1.5 1.0 0.5

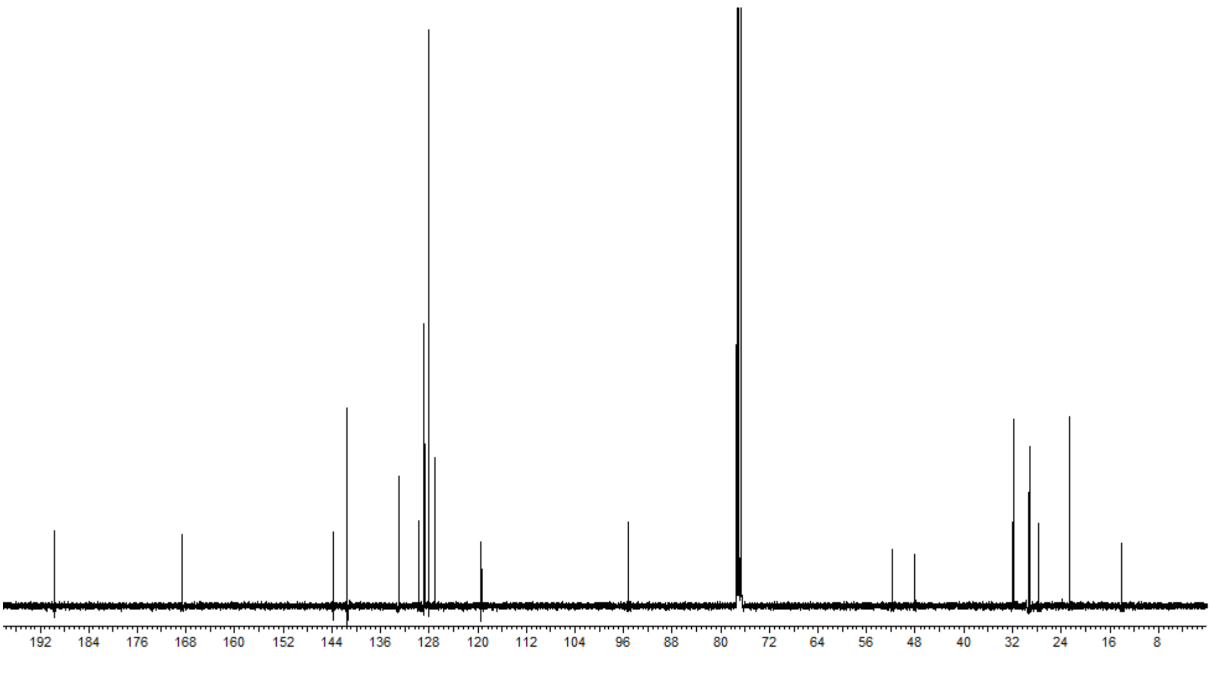
192 184 176 168 160 152 144 136 128 120 112 104 96 88 80 72 64 56 48 40 32 24 16 8

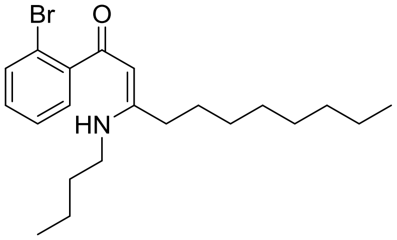

Br
O
HN

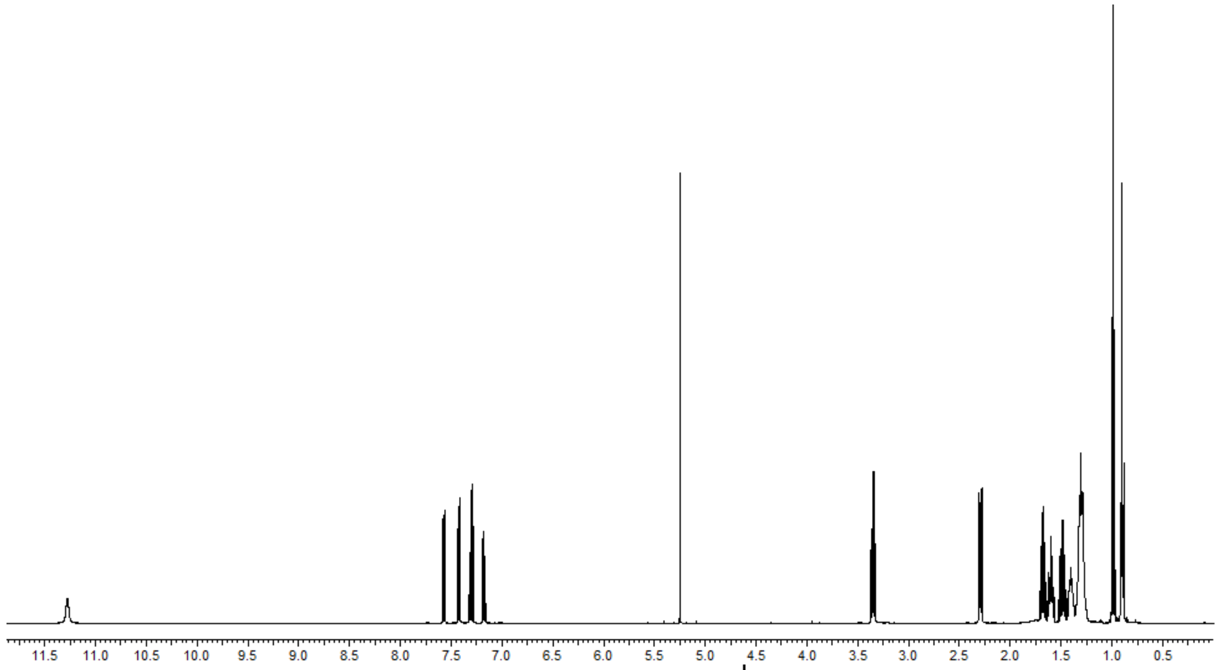

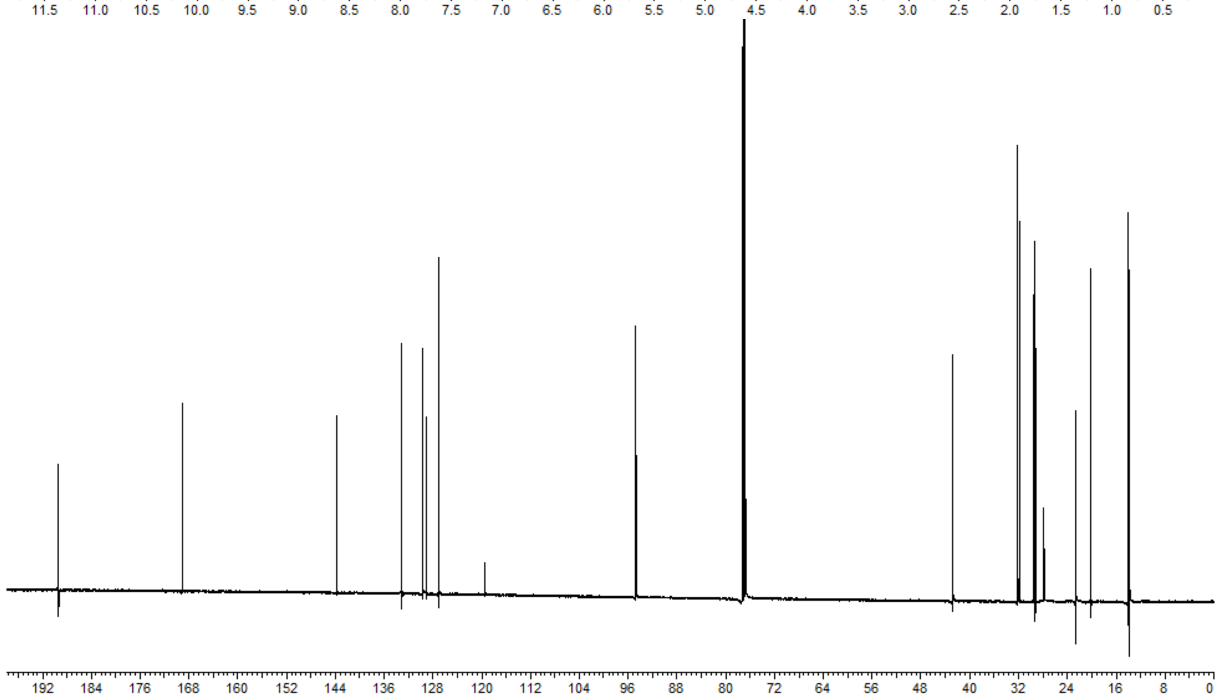

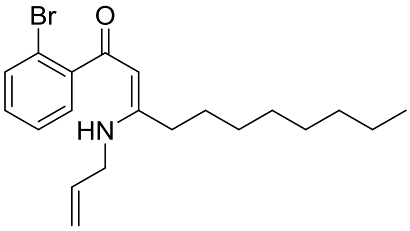
Br
O
HN

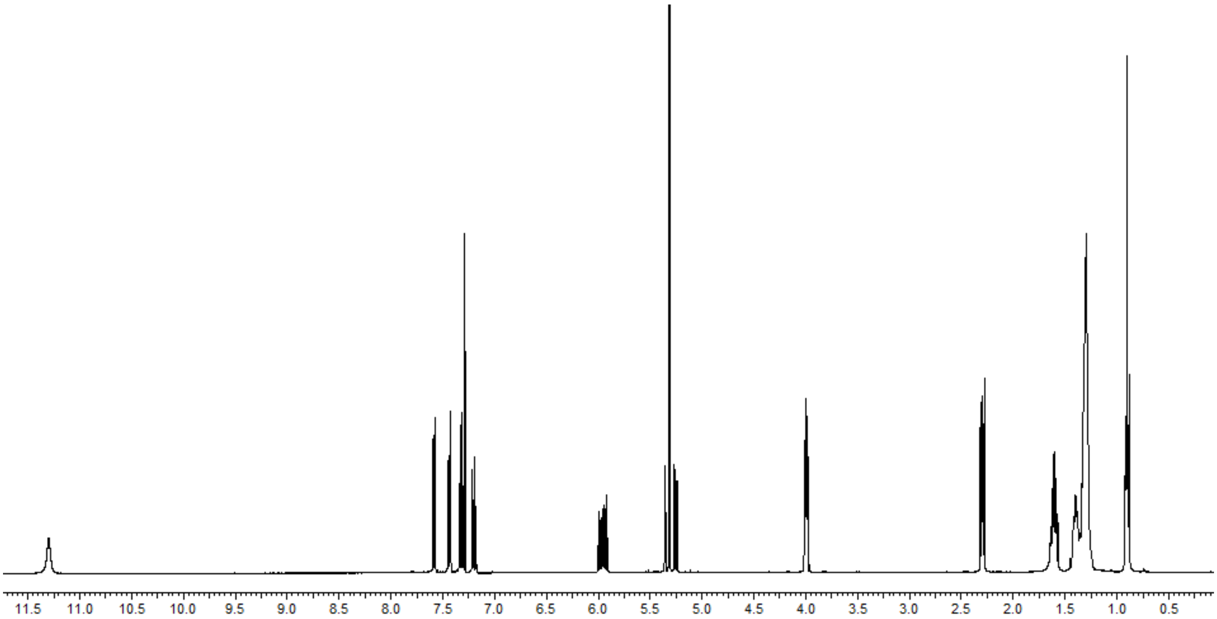
11.5 11.0 10.5 10.0 9.5 9.0 8.5 8.0 7.5 7.0 6.5 6.0 5.5 5.0 4.5 4.0 3.5 3.0 2.5 2.0 1.5 1.0 0.5

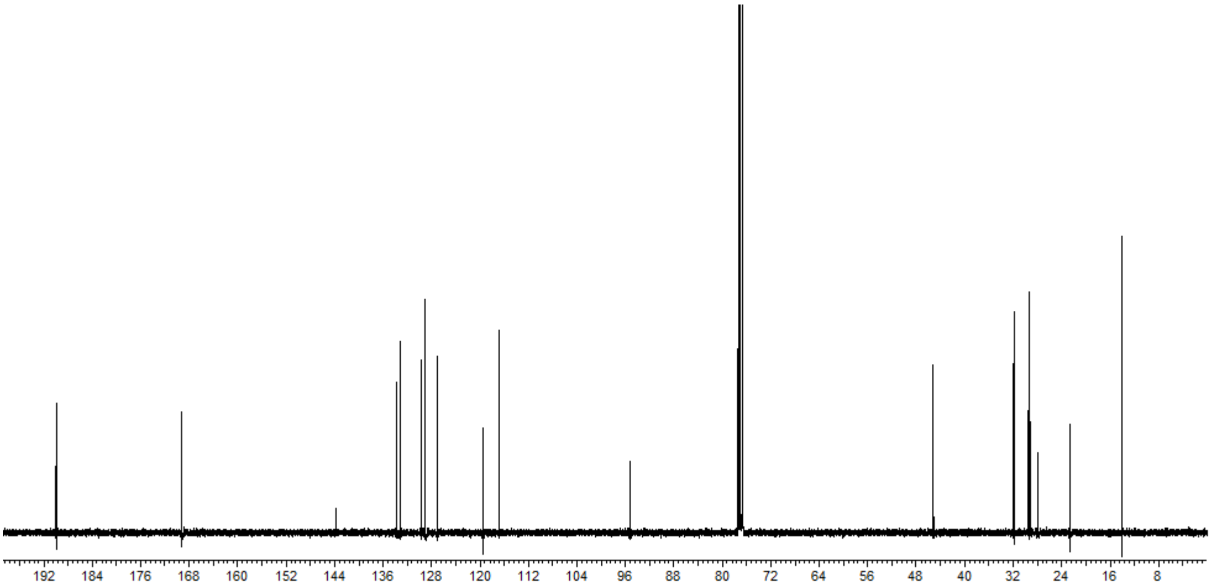
192 184 176 168 160 152 144 136 128 120 112 104 96 88 80 72 64 56 48 40 32 24 16 8

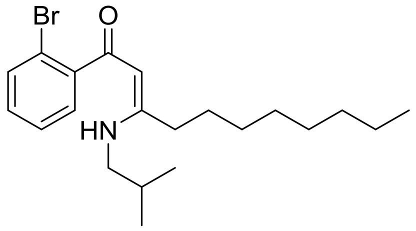
Br
O
HN

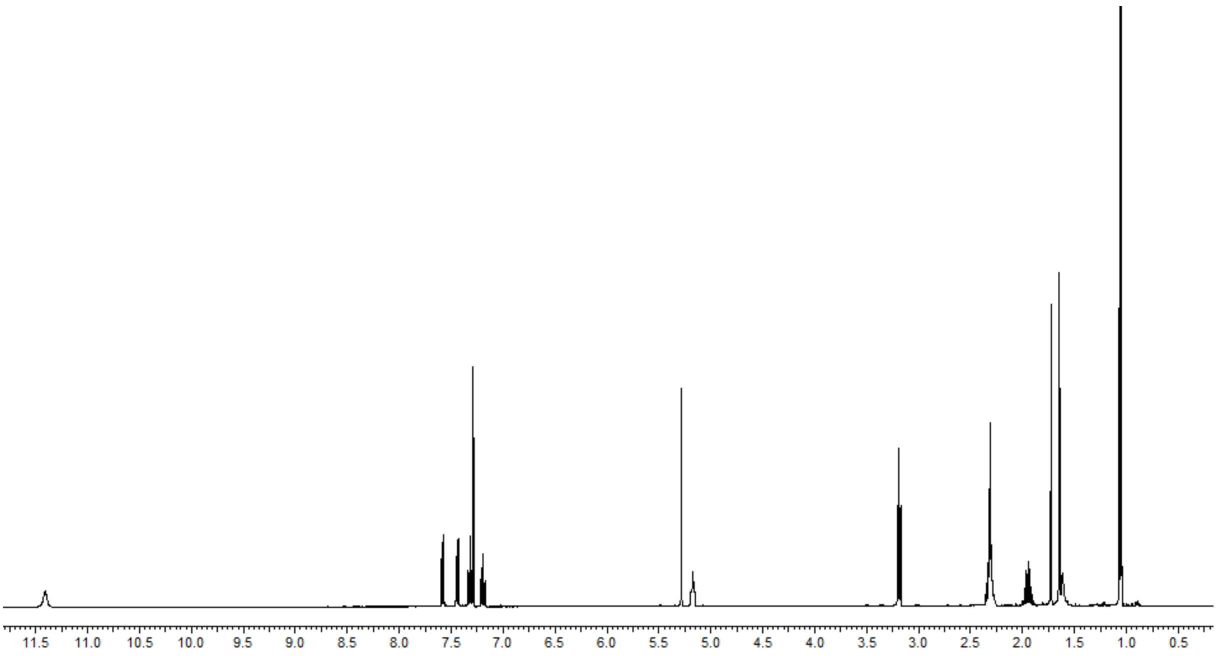
11.5 11.0 10.5 10.0 9.5 9.0 8.5 8.0 7.5 7.0 6.5 6.0 5.5 5.0 4.5 4.0 3.5 3.0 2.5 2.0 1.5 1.0 0.5

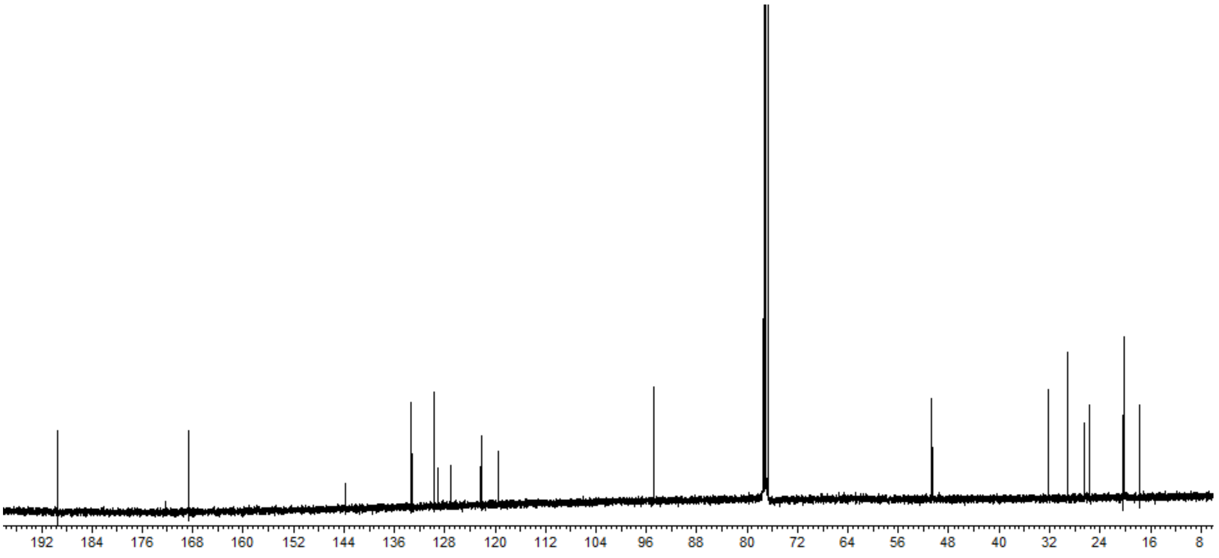
192 184 176 168 160 152 144 136 128 120 112 104 96 88 80 72 64 56 48 40 32 24 16 8

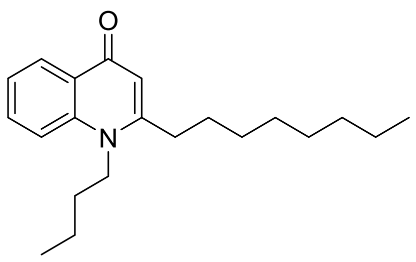
O
N

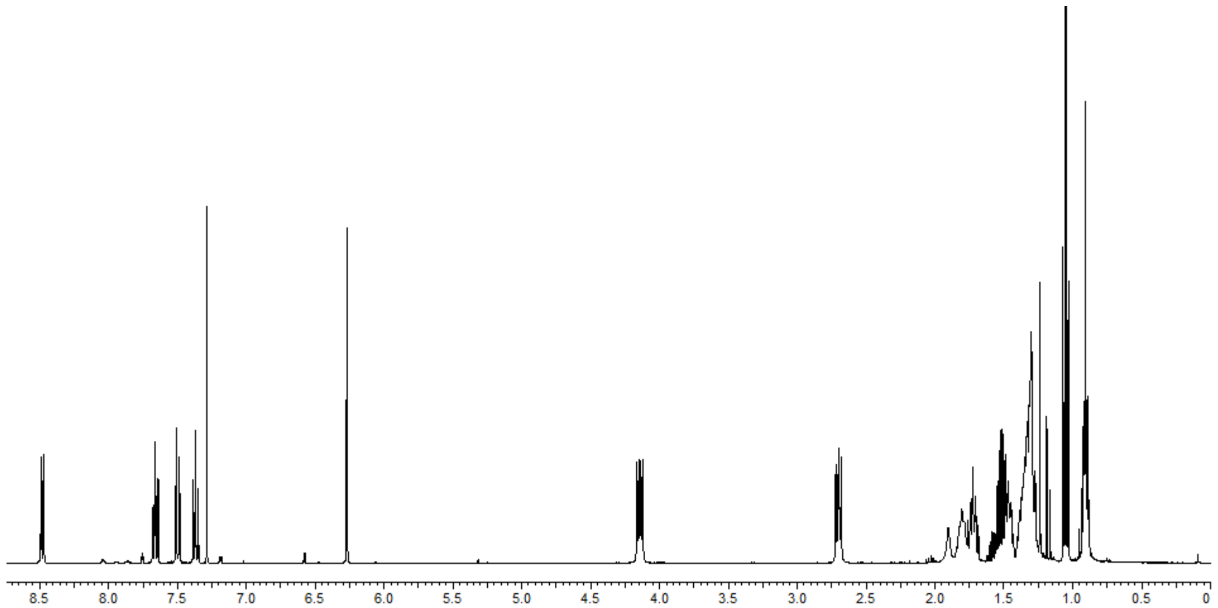
8.5 8.0 7.5 7.0 6.5 6.0 5.5 5.0 4.5 4.0 3.5 3.0 2.5 2.0 1.5 1.0 0.5 0

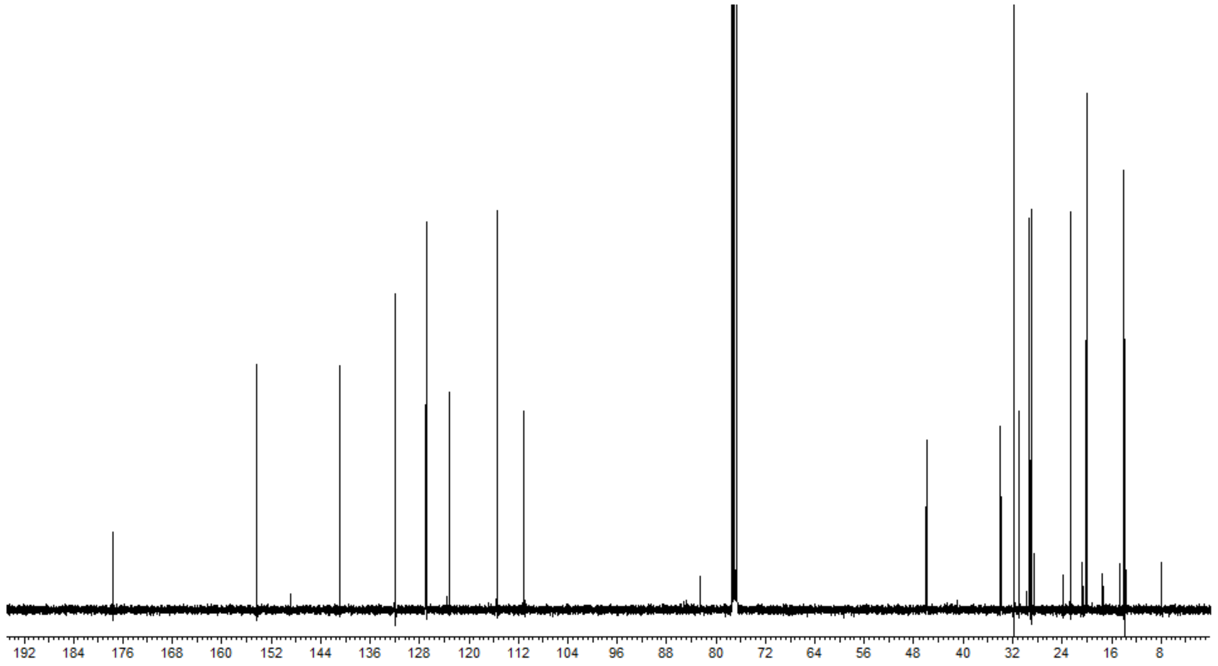
192 184 176 168 160 152 144 136 128 120 112 104 96 88 80 72 64 56 48 40 32 24 16 8

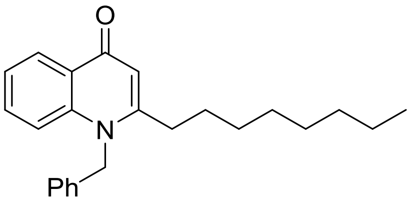
O
N
Ph

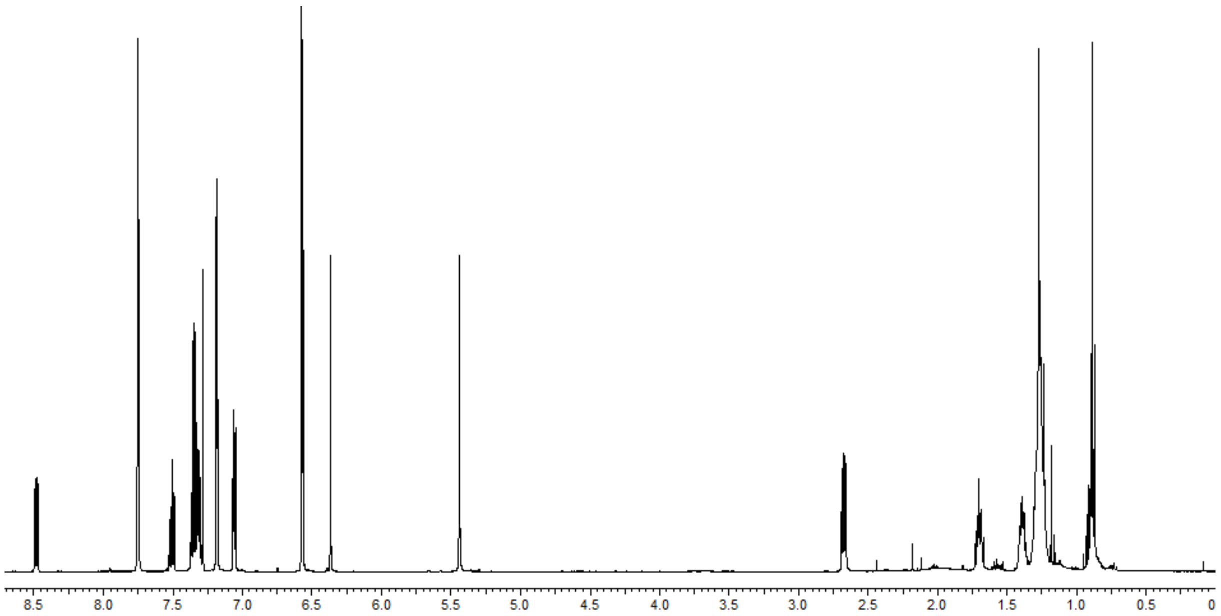
8.5 8.0 7.5 7.0 6.5 6.0 5.5 5.0 4.5 4.0 3.5 3.0 2.5 2.0 1.5 1.0 0.5 0

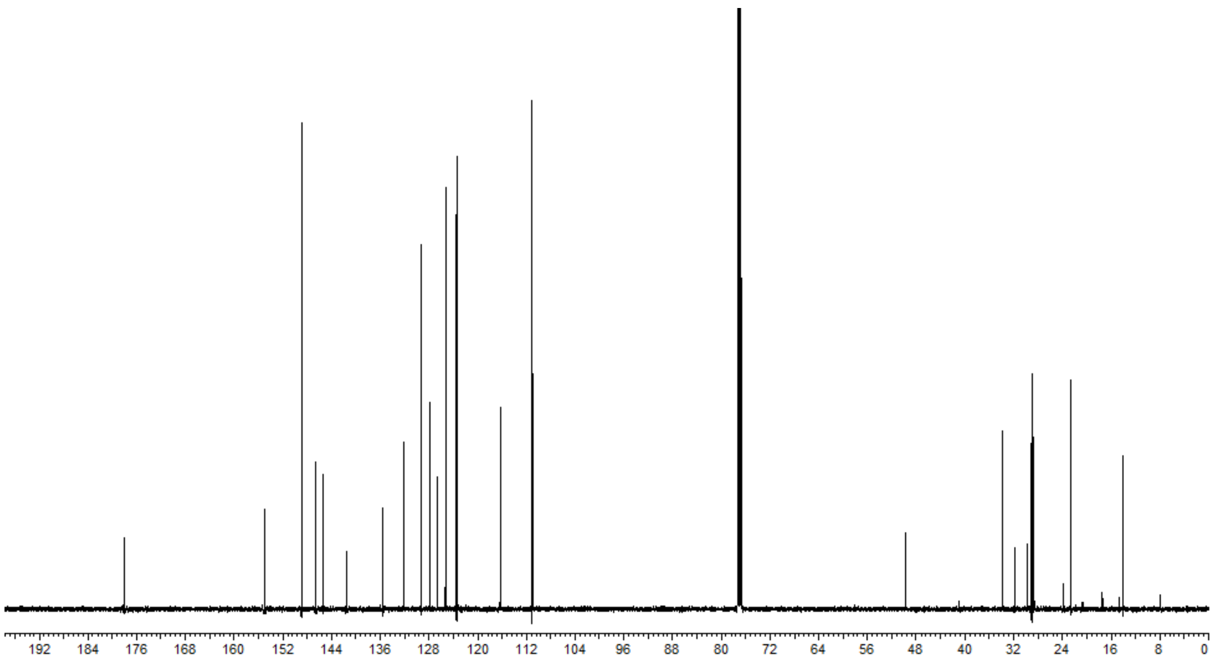
192 184 176 168 160 152 144 136 128 120 112 104 96 88 80 72 64 56 48 40 32 24 16 8 0

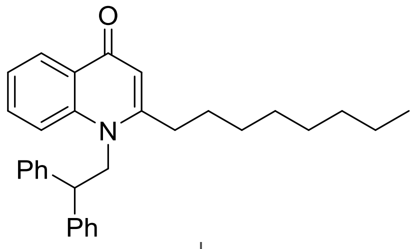
O
N
Ph
Ph

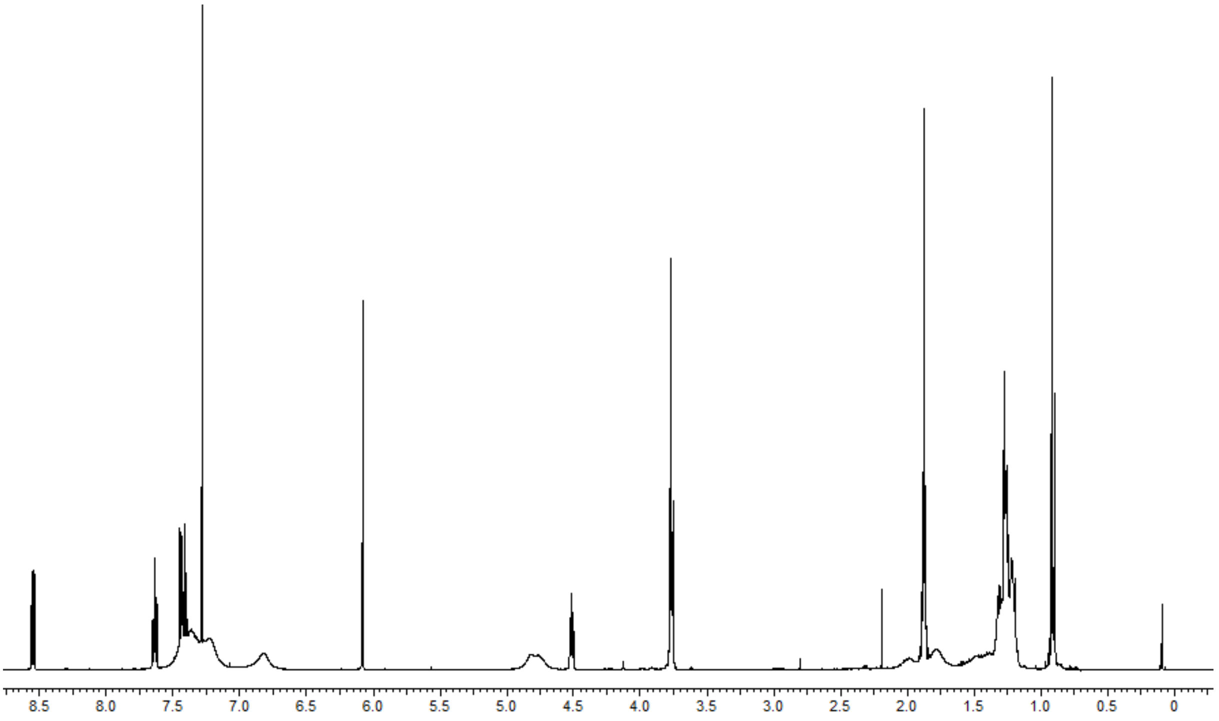
8.5 8.0 7.5 7.0 6.5 6.0 5.5 5.0 4.5 4.0 3.5 3.0 2.5 2.0 1.5 1.0 0.5 0

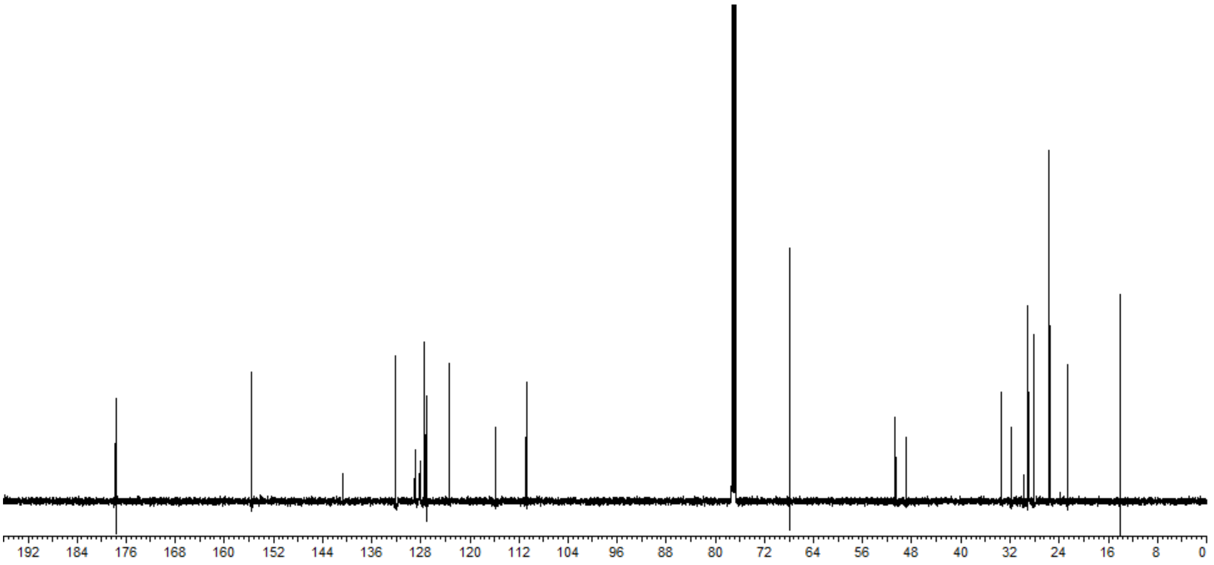
192 184 176 168 160 152 144 136 128 120 112 104 96 88 80 72 64 56 48 40 32 24 16 8 0

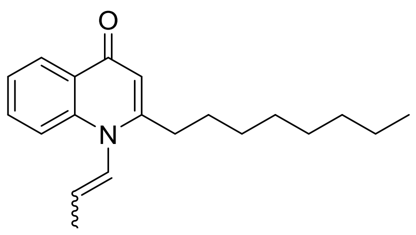
O
N

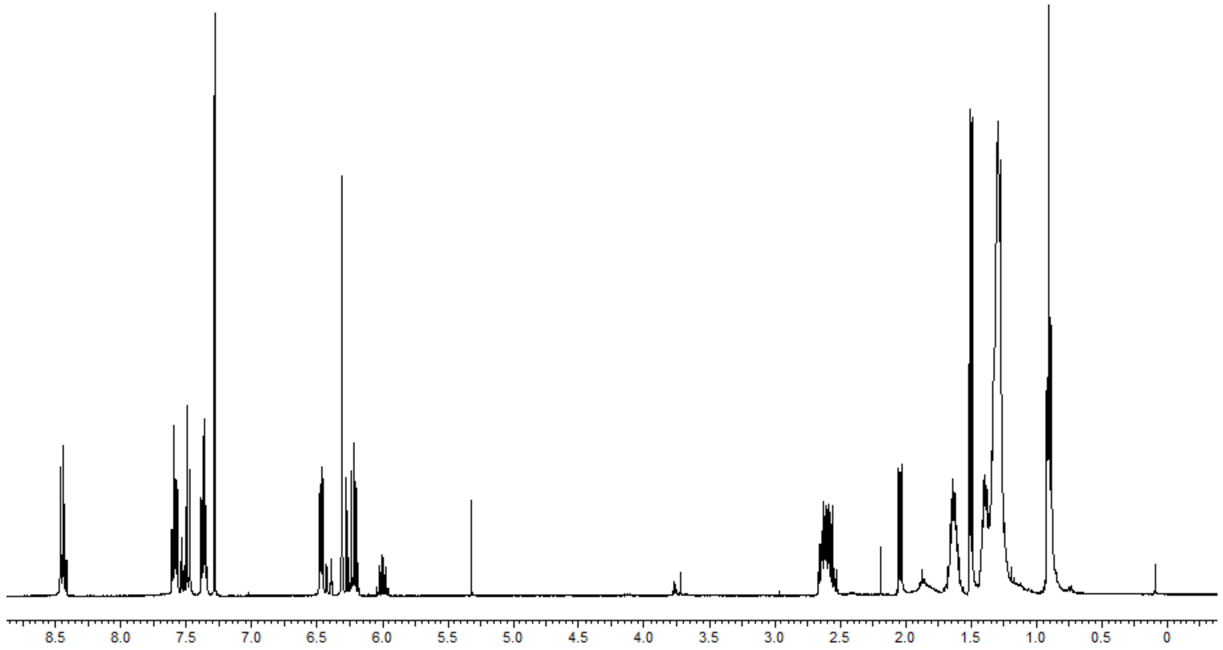
8.5 8.0 7.5 7.0 6.5 6.0 5.5 5.0 4.5 4.0 3.5 3.0 2.5 2.0 1.5 1.0 0.5 0

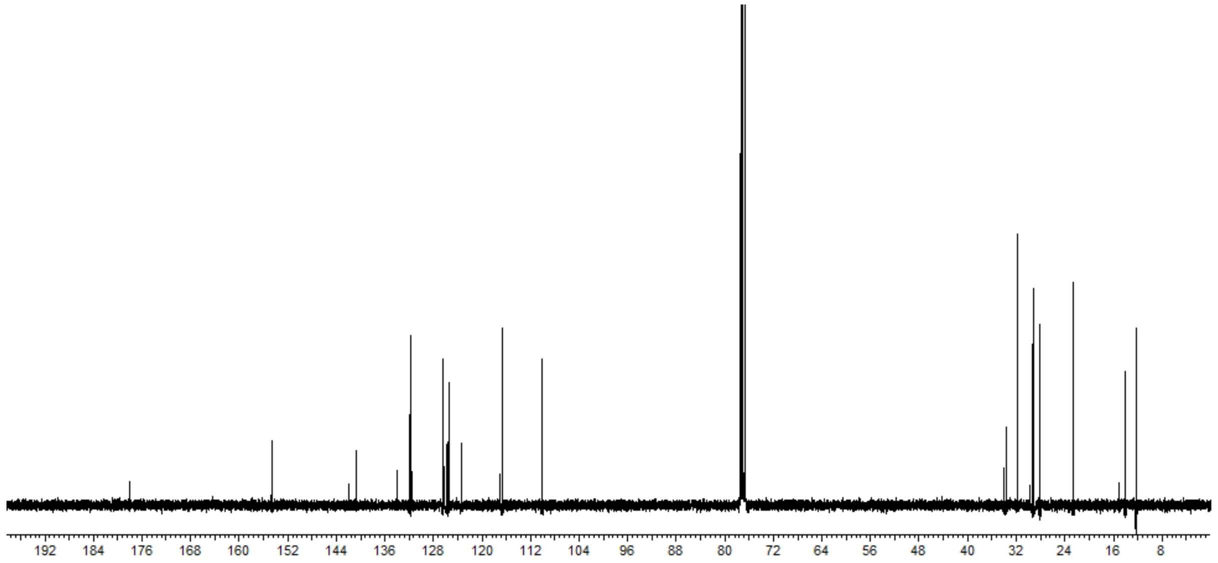
192 184 176 168 160 152 144 136 128 120 112 104 96 88 80 72 64 56 48 40 32 24 16 8

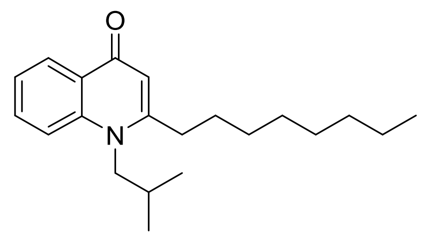
O
N

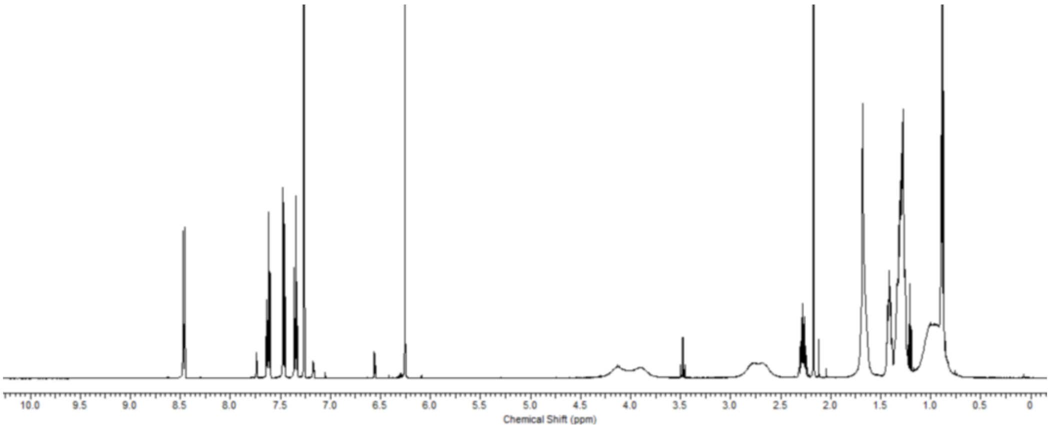
10.0 9.5 9.0 8.5 8.0 7.5 7.0 6.5 6.0 5.5 5.0 4.5 4.0 3.5 3.0 2.5 2.0 1.5 1.0 0.5 0
Chemical Shift (ppm)

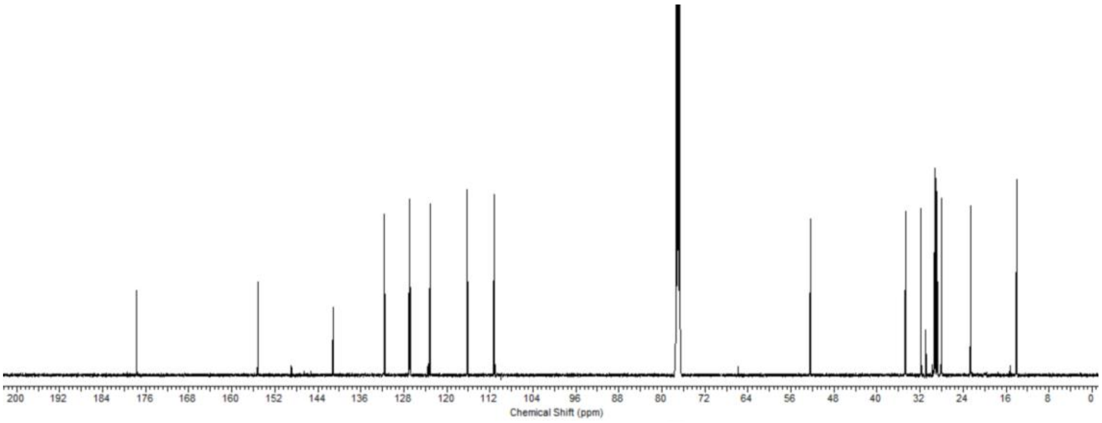
200 192 184 176 168 160 152 144 136 128 120 112 104 96 88 80 72 64 56 48 40 32 24 16 8 0
Chemical Shift (ppm)

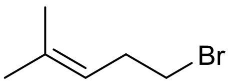
Br

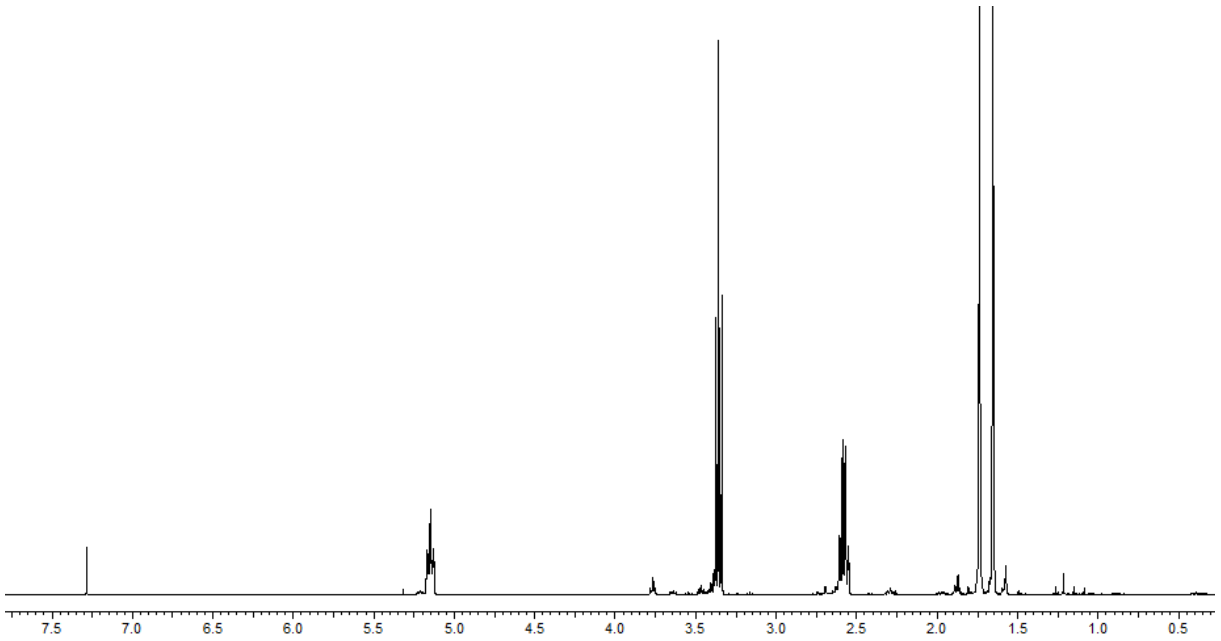
7.5 7.0 6.5 6.0 5.5 5.0 4.5 4.0 3.5 3.0 2.5 2.0 1.5 1.0 0.5

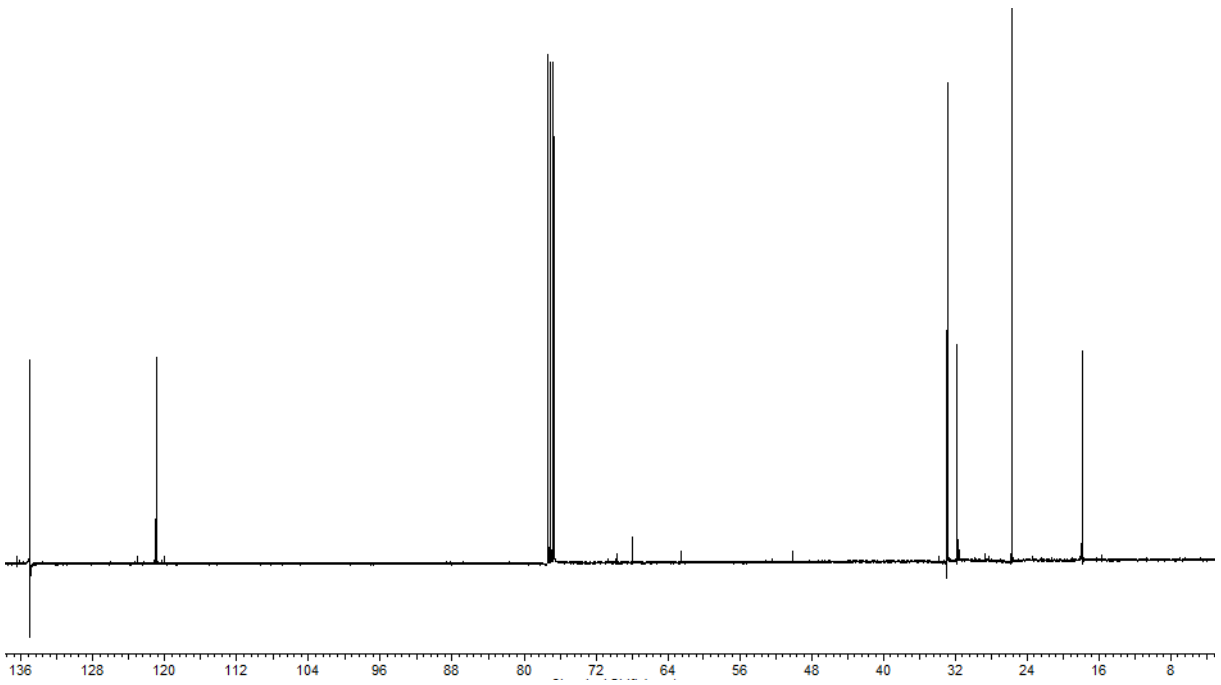
136 128 120 112 104 96 88 80 72 64 56 48 40 32 24 16 8

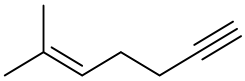

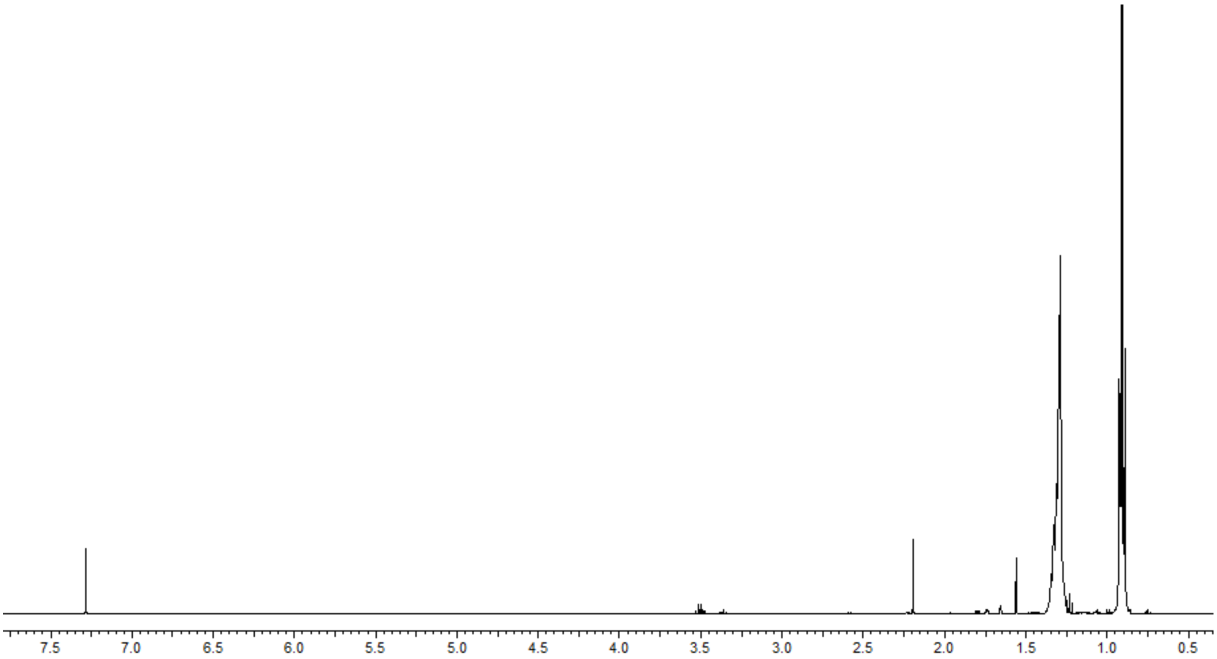

7.5
7.0
6.5
6.0
5.5
5.0
4.5
4.0
3.5
3.0
2.5
2.0
1.5
1.0
0.5

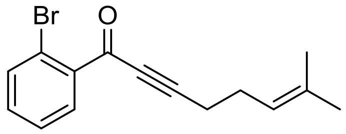
Br
O

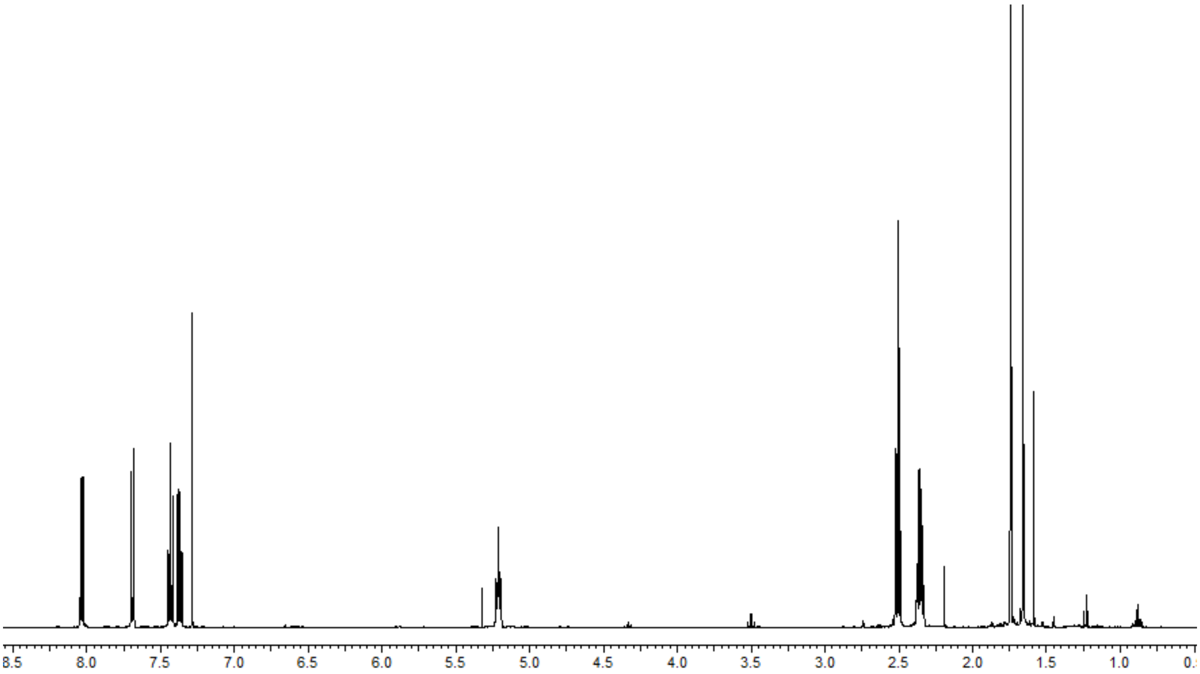
8.5 8.0 7.5 7.0 6.5 6.0 5.5 5.0 4.5 4.0 3.5 3.0 2.5 2.0 1.5 1.0

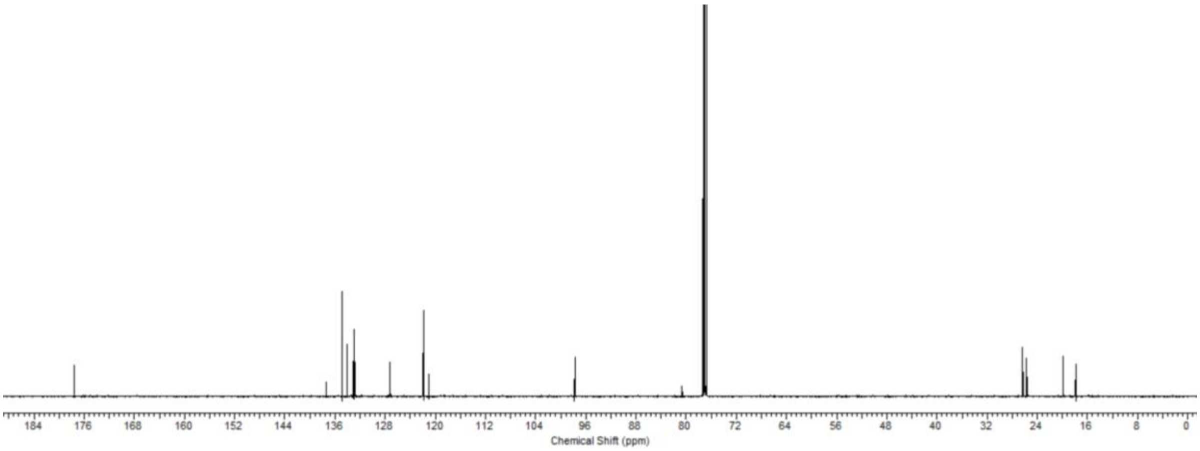
184 176 168 160 152 144 136 128 120 112 104 96 88 80 72 64 56 48 40 32 24 16 8 0
Chemical Shift (ppm)

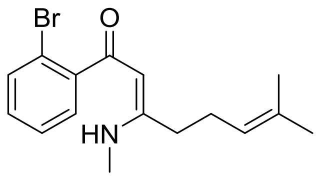
Br
O
HN

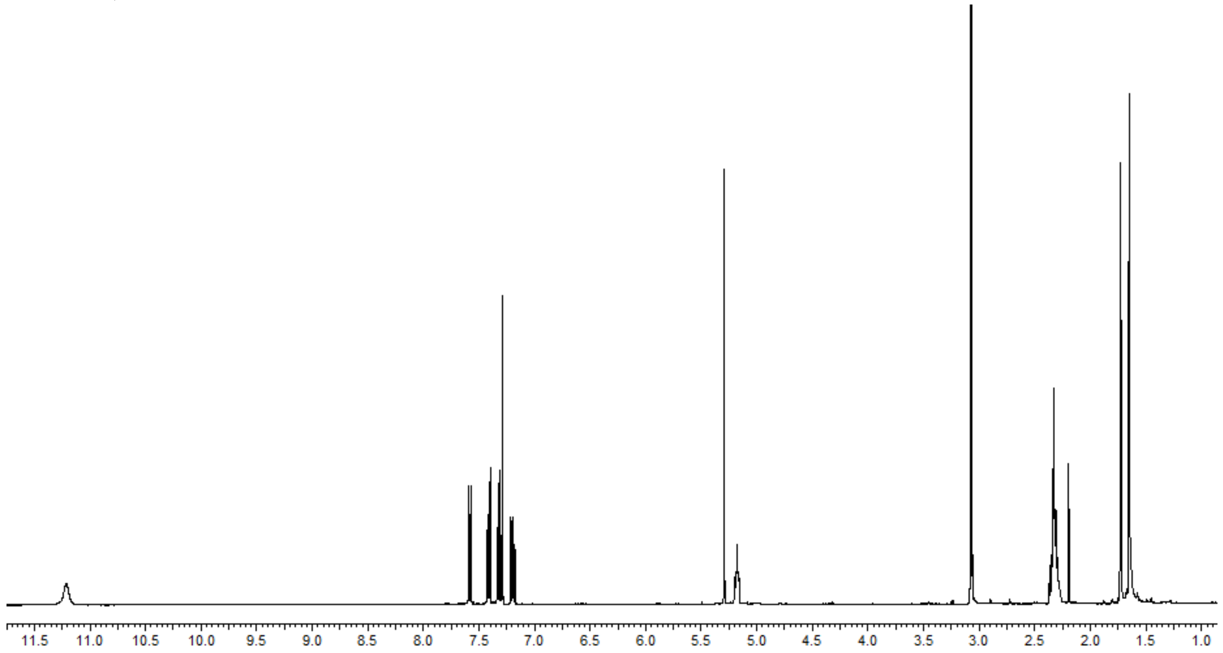
11.5 11.0 10.5 10.0 9.5 9.0 8.5 8.0 7.5 7.0 6.5 6.0 5.5 5.0 4.5 4.0 3.5 3.0 2.5 2.0 1.5 1.0

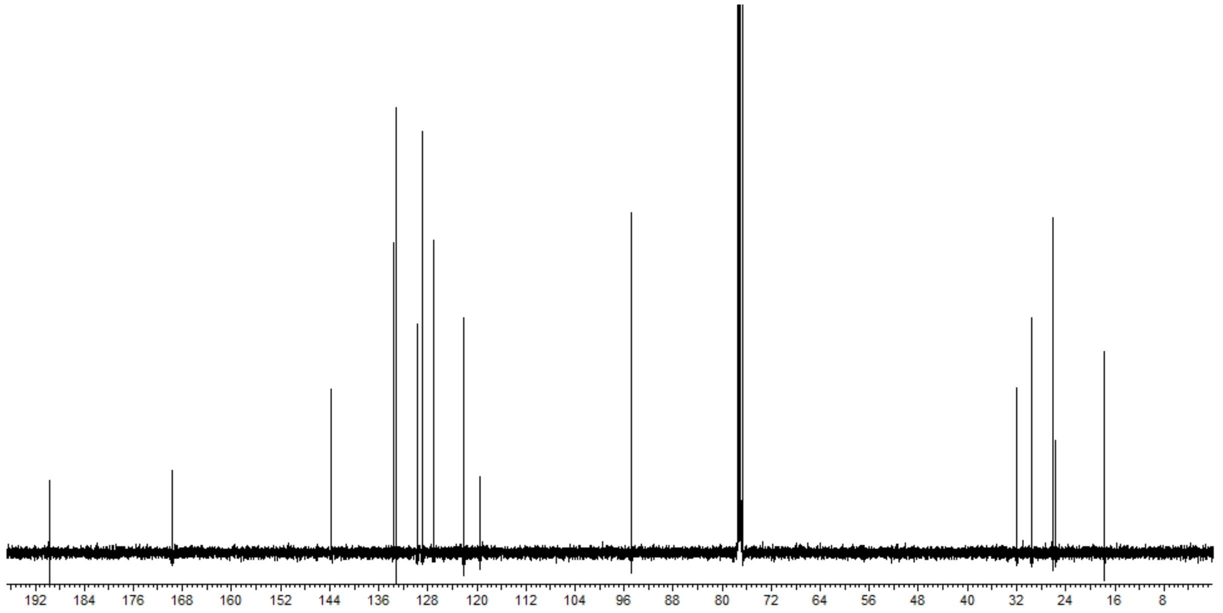
192 184 176 168 160 152 144 136 128 120 112 104 96 88 80 72 64 56 48 40 32 24 16 8

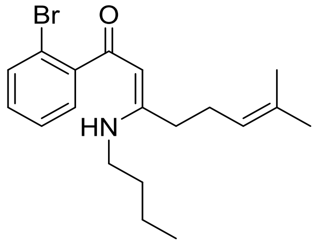
Br
O
HN

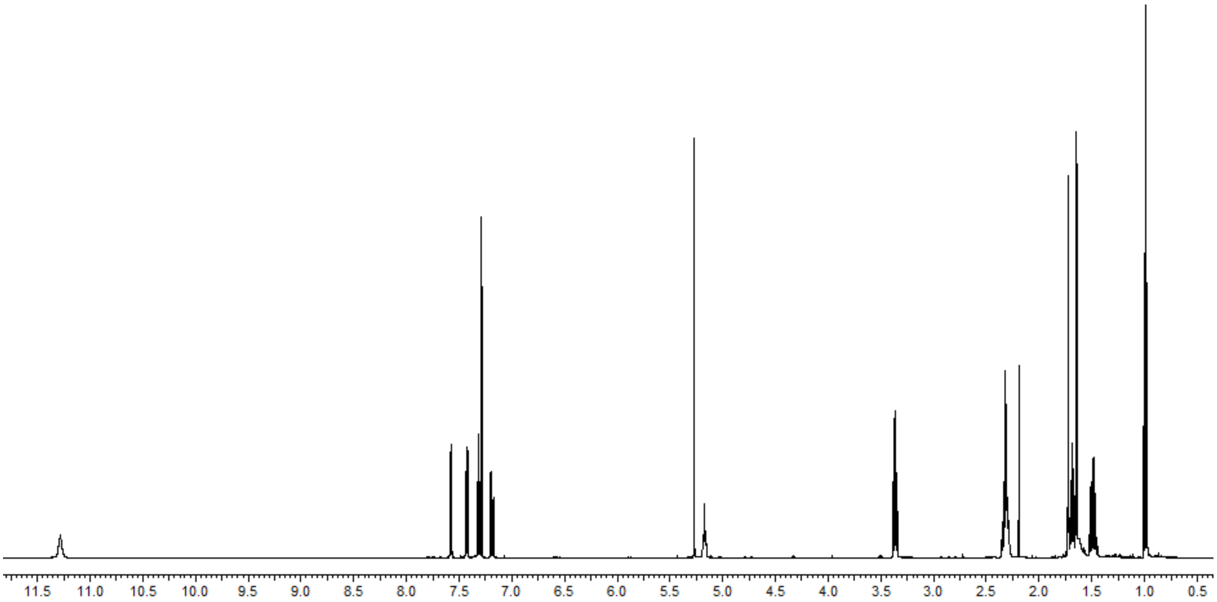
11.5 11.0 10.5 10.0 9.5 9.0 8.5 8.0 7.5 7.0 6.5 6.0 5.5 5.0 4.5 4.0 3.5 3.0 2.5 2.0 1.5 1.0 0.5

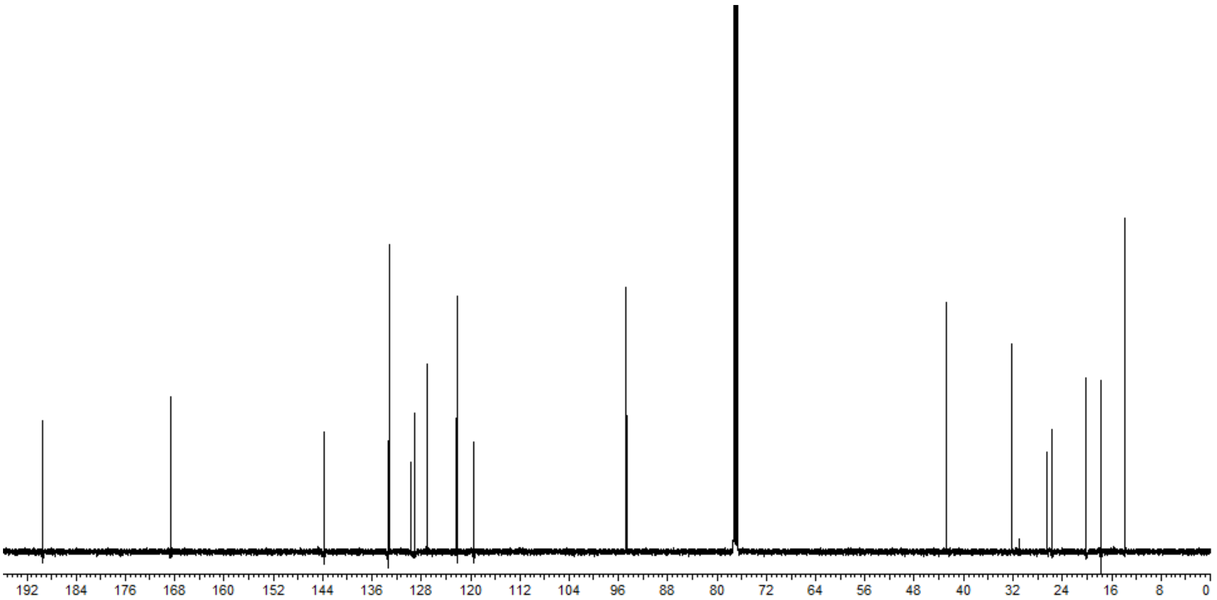
192 184 176 168 160 152 144 136 128 120 112 104 96 88 80 72 64 56 48 40 32 24 16 8 0

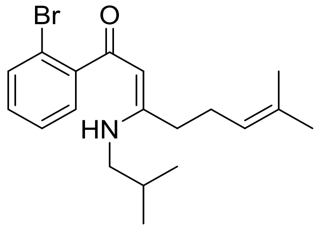
Br
O
HN

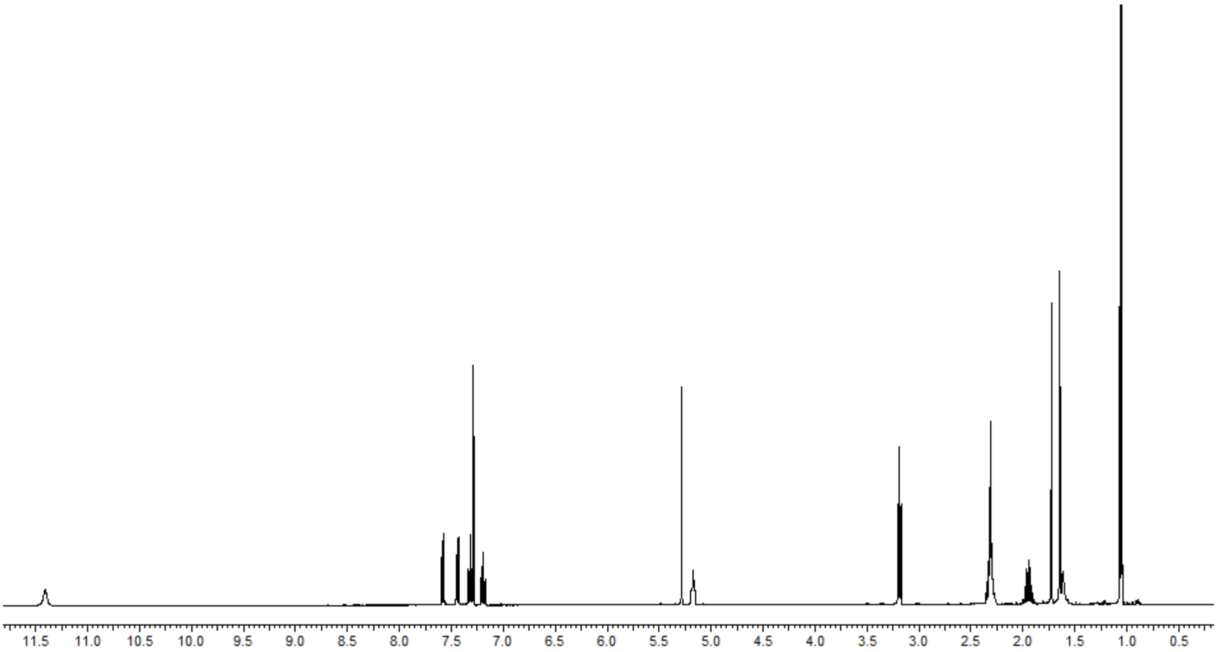
11.5
11.0
10.5
10.0
9.5
9.0
8.5
8.0
7.5
7.0
6.5
6.0
5.5
5.0
4.5
4.0
3.5
3.0
2.5
2.0
1.5
1.0
0.5

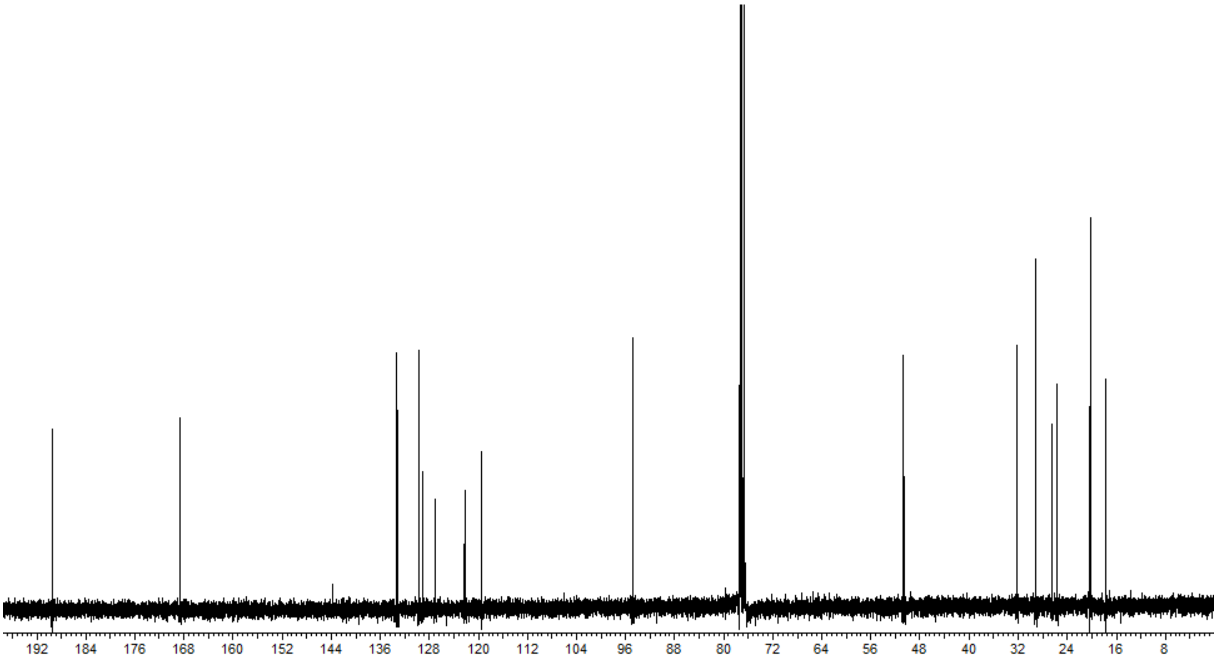
192
184
176
168
160
152
144
136
128
120
112
104
96
88
80
72
64
56
48
40
32
24
16
8

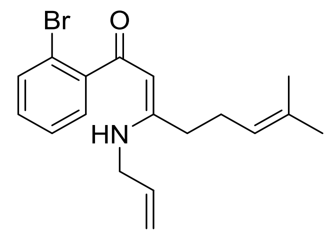
Br
O
HN

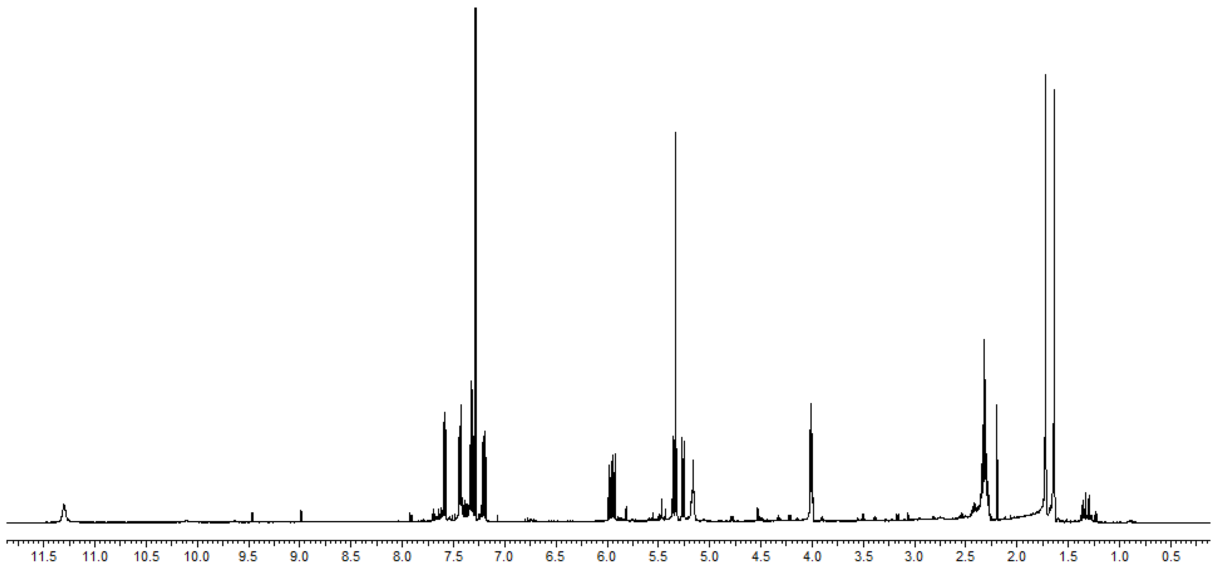

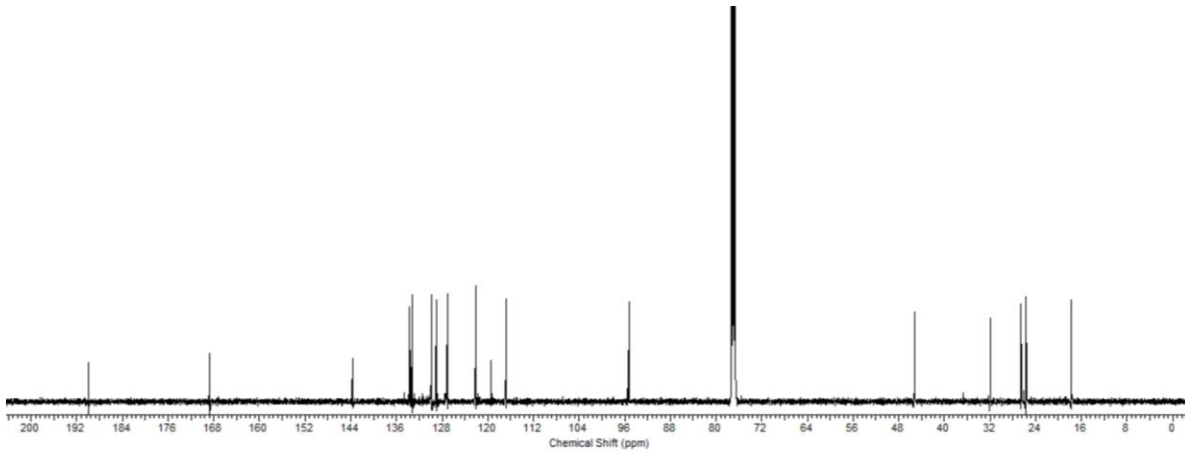
Chemical Shift (ppm)

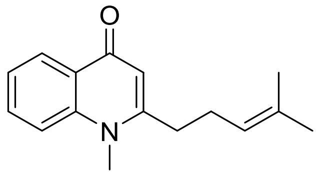
O
N

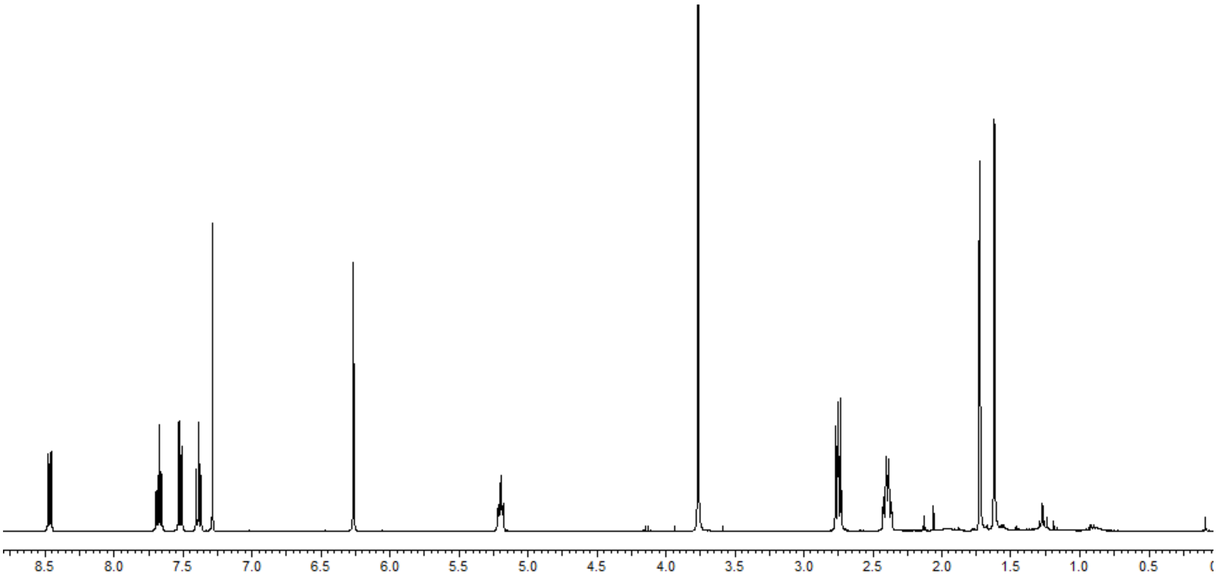
8.5 8.0 7.5 7.0 6.5 6.0 5.5 5.0 4.5 4.0 3.5 3.0 2.5 2.0 1.5 1.0 0.5

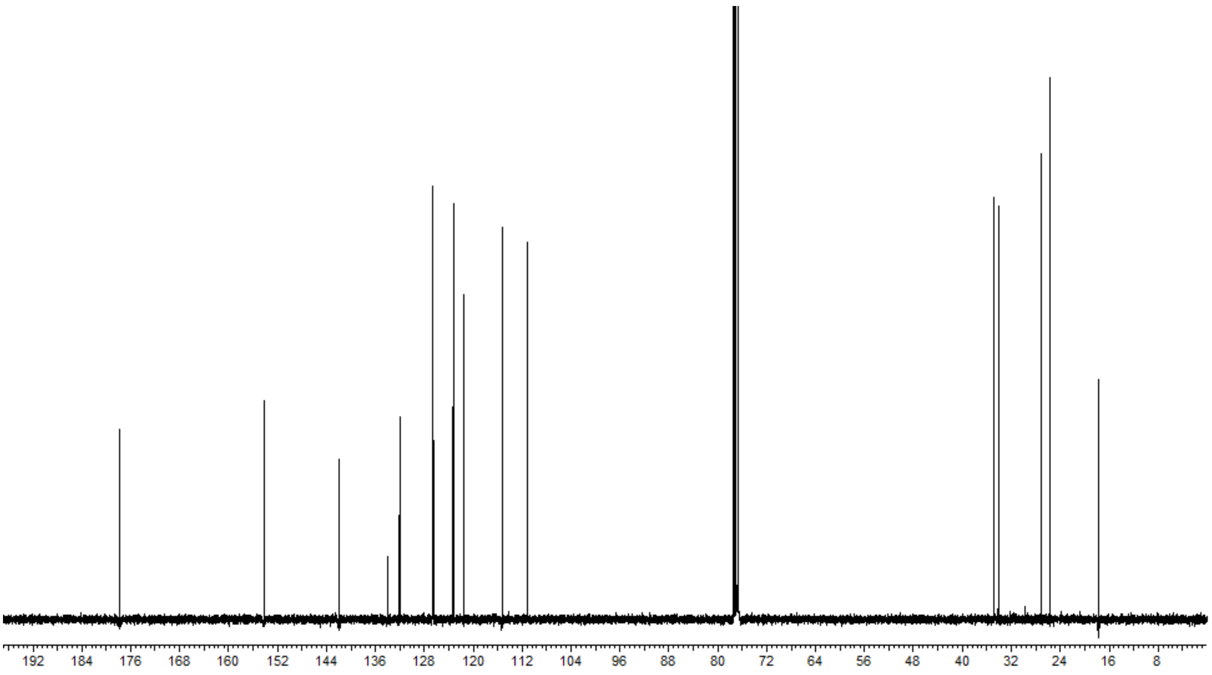
192 184 176 168 160 152 144 136 128 120 112 104 96 88 80 72 64 56 48 40 32 24 16 8

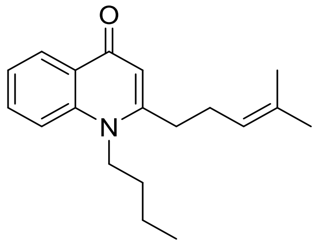
O
N

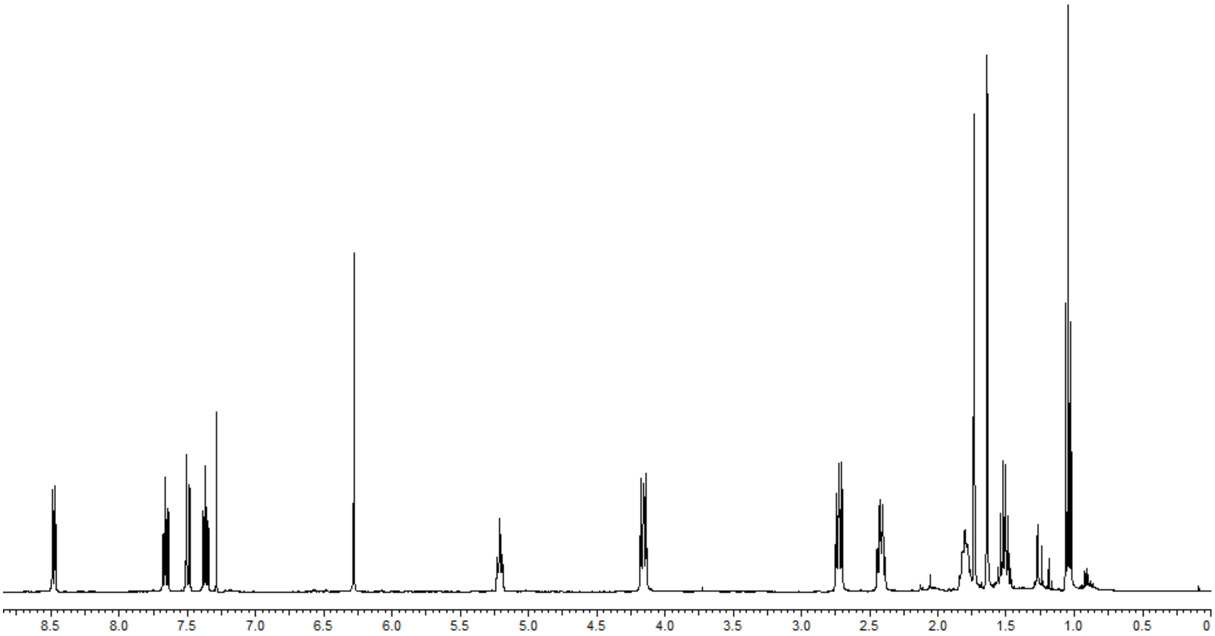
8.5 8.0 7.5 7.0 6.5 6.0 5.5 5.0 4.5 4.0 3.5 3.0 2.5 2.0 1.5 1.0 0.5 0

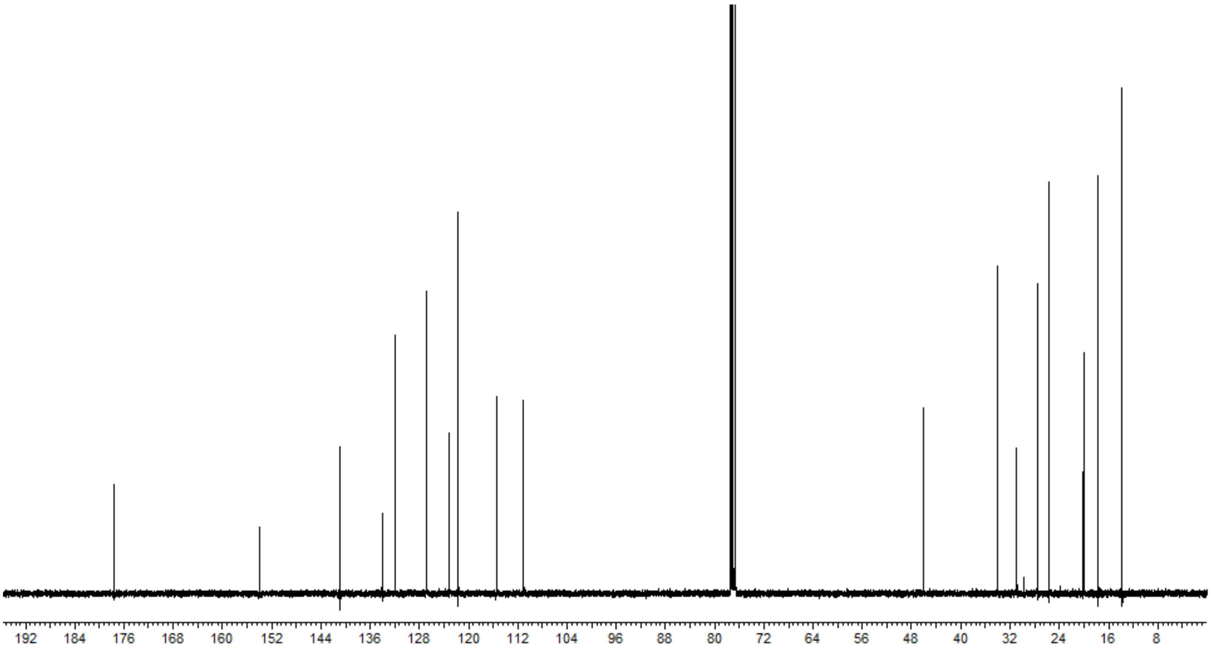
192 184 176 168 160 152 144 136 128 120 112 104 96 88 80 72 64 56 48 40 32 24 16 8

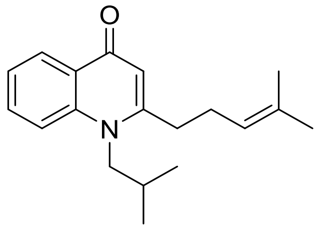
O
N

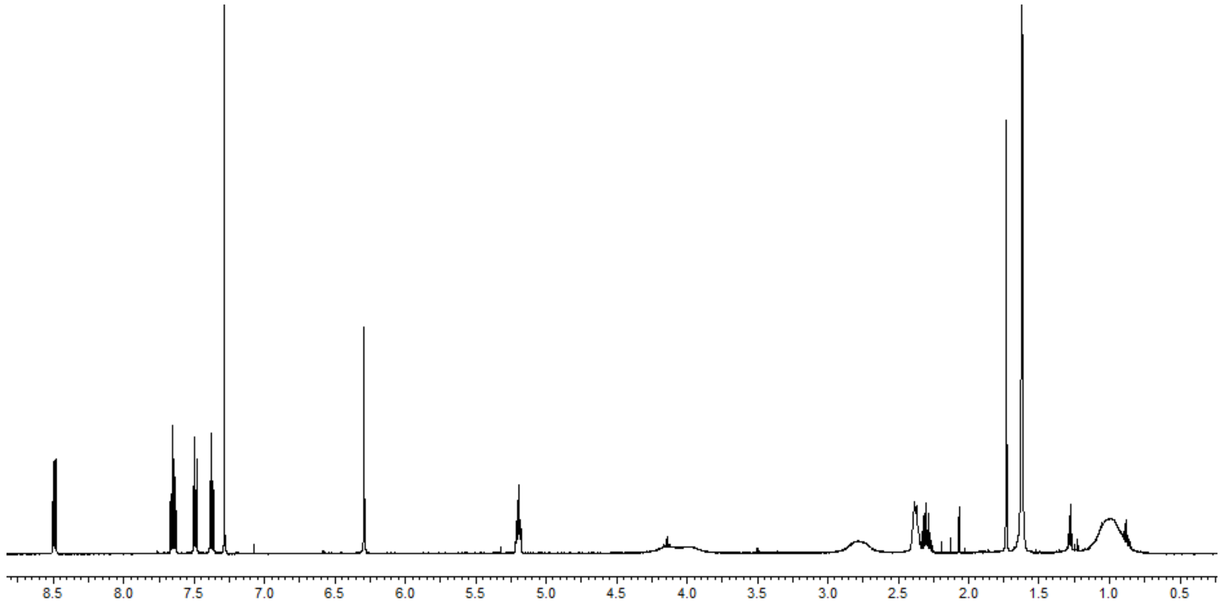
8.5
8.0
7.5
7.0
6.5
6.0
5.5
5.0
4.5
4.0
3.5
3.0
2.5
2.0
1.5
1.0
0.5

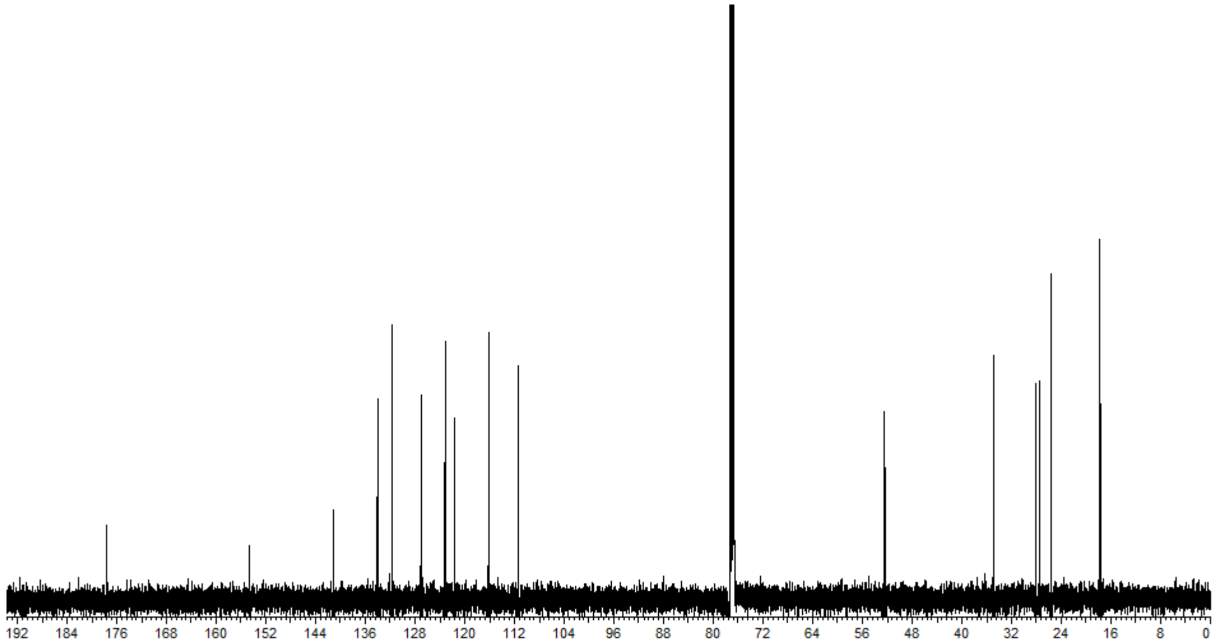
192
184
176
168
160
152
144
136
128
120
112
104
96
88
80
72
64
56
48
40
32
24
16
8
0

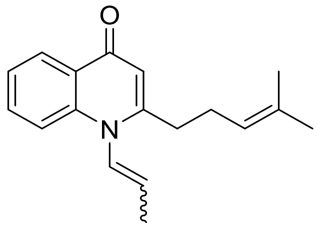
O
N

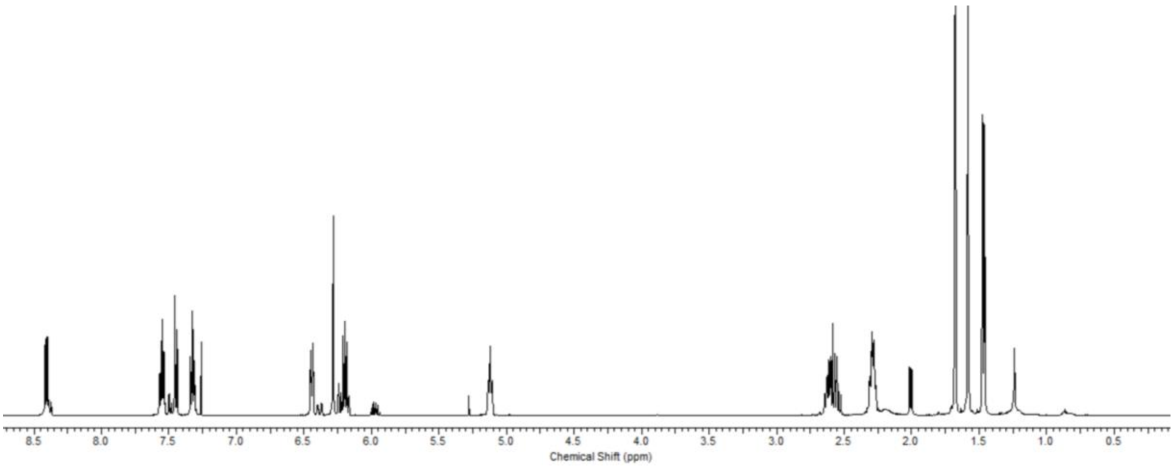
8.5 8.0 7.5 7.0 6.5 6.0 5.5 5.0 4.5 4.0 3.5 3.0 2.5 2.0 1.5 1.0 0.5
Chemical Shift (ppm)

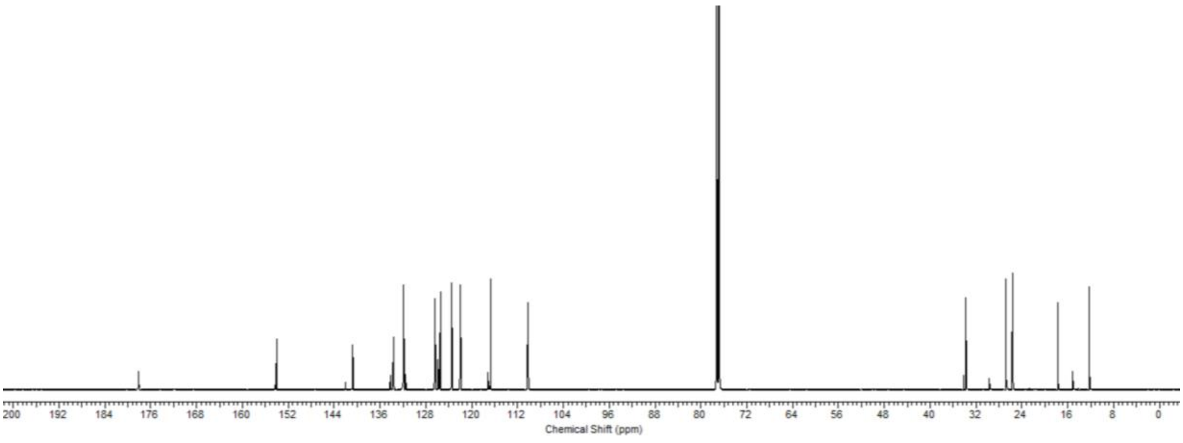
200 192 184 176 168 160 152 144 136 128 120 112 104 96 88 80 72 64 56 48 40 32 24 16 8 0
Chemical Shift (ppm)

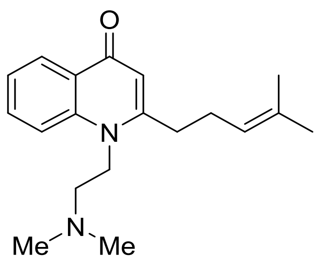
O
N
Me
N
Me

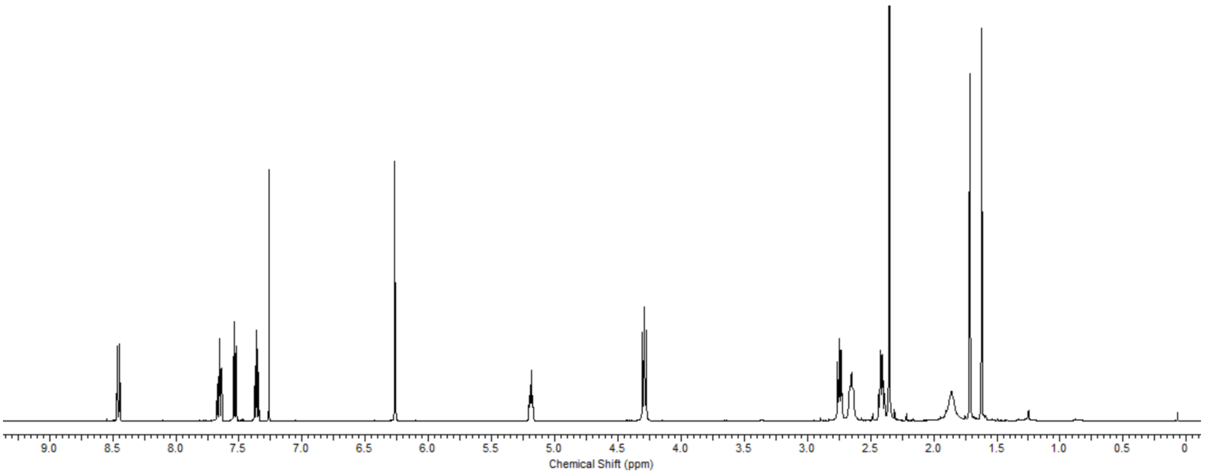
9.0 8.5 8.0 7.5 7.0 6.5 6.0 5.5 5.0 4.5 4.0 3.5 3.0 2.5 2.0 1.5 1.0 0.5 0
Chemical Shift (ppm)

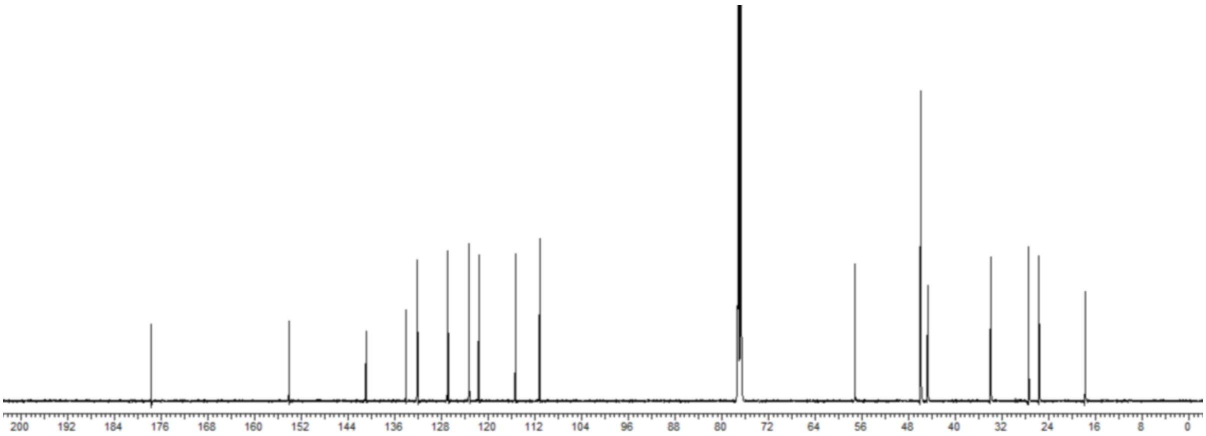
200 192 184 176 168 160 152 144 136 128 120 112 104 96 88 80 72 64 56 48 40 32 24 16 8 0
